# Synthesis and anti-proliferative activity of new E7010 tethered urea congeners as potential tubulin inhibitors and apoptosis inducers

**DOI:** 10.1039/d5ra09372d

**Published:** 2026-01-19

**Authors:** Shaik Taj, Ganga Reddy Velma, Srinivasa Reddy Telukutla, Satyaveni Malasala, Anjali Sharma, Irfan Khan, Mohd Adil Shareef, Suresh K. Bhargava, Magdalena Plebanski, Bathini Nagendra Babu, Ahmed Kamal

**Affiliations:** a Academy of Scientific and Innovative Research (AcSIR), CSIR-Human Resource Development Centre (CSIR-HRDC) Campus Ghaziabad 201 002 Uttar Pradesh India; b Fluoro-Agrochemicals, CSIR-Indian Institute of Chemical Technology (IICT) Hyderabad 500 007 India; c Department of Pharmacology and Toxicology, College of Pharmacy, University of Arizona Tucson 85721 AZ USA velmagangareddy47@gmail.com vgreddy@arizona.edu; d Centre for Advanced Materials & Industrial Chemistry (CAMIC), School of Science, RMIT University GPO Box 2476 Melbourne 3001 Australia; e School of Health and Biomedical Sciences, RMIT University Melbourne 3083 Australia; f Department of Anatomy & Cell Biology, The Brody School of Medicine, East Carolina University Greenville NC USA; g Guru Gobind Singh College of Pharmacy Yamunanagar (135001) Haryana India; h Department of Pharmacy, Birla Institute of Technology and Science-Pilani Hyderabad 500078 India ahmedkamal@hyderabad.bits-pilani.ac.in ahmedkamal915@gmail.com

## Abstract

A series of twenty-four 1-phenyl-3-(2-phenylpyridin-3-yl)urea congeners (6a–x) have been designed and synthesized. All these compounds were evaluated for their anti-cancer activity against four human cancer cell lines, including prostate cancer (DU-145), lung cancer (A549), cervical cancer (HeLa) and breast cancer (MDA-MB-231). Compound 6q emerged as the most potent in the series, showing consistently strong cytotoxicity against all tested cell lines, with IC50 values ranging from 2.03 to 8.14 µM. Further investigation into the mechanism of action of compound 6q revealed it can arrest cell cycle progression at the G2/M phase. Immunocytochemistry studies showed a notable disruption of microtubule structure in cells treated with 6q. Molecular docking studies provided strong evidence that compound 6q works by binding to the colchicine binding site of β-tubulin, with sequential hydrophilic and hydrophobic interactions. Additionally, the effects of 6q on cell migration and its ability to induce apoptosis in A549 cells were examined using nuclear and mitochondrial staining techniques.

## Abstract 2

E7010-derived urea analogues were designed *via* sulfonamide-to-urea replacement and aniline trimming, yielding potent tubulin inhibitors and apoptosis inducers.

## Graphical abstract

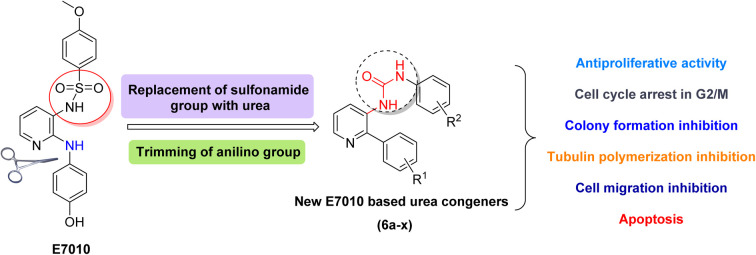

## 1 Introduction

**Fig. 1:**
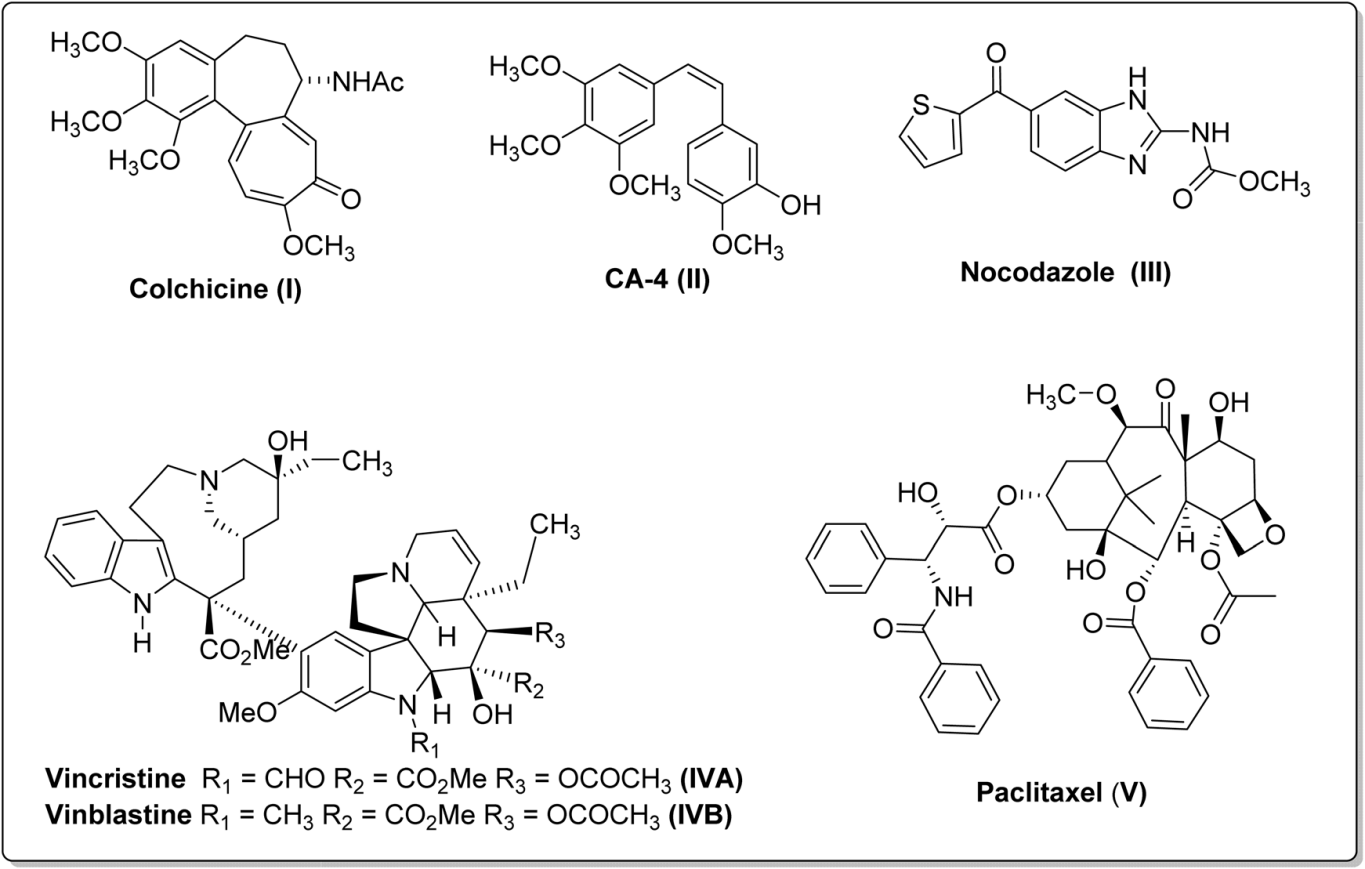
Some well-known examples of tubulin inhibitors.

Despite extensive efforts in the development of anti-cancer drugs, cancer remains a life-threatening disease.^1^ Unfortunately, existing anticancer drugs suffer from multiple drawbacks, including serious adverse effects, drug resistance, non-selectivity, and poor bioavailability, which supports the need for the development of new and better drugs.^2–4^ Microtubules are formed through the polymerization of α–β tubulin heterodimers. They are one of the well-established and validated targets for treating cancers.^5^ By disrupting the dynamic stability of microtubules, anti-tubulin agents function as spindle poisons, leading to G2/M phase cell cycle arrest and apoptotic cell death.^6,7^ A wide range of reports in the literature have highlighted that naturally occurring and synthetic hybrids can be potent tubulin agents inhibitors.^8–10^ Notably, compounds such as Colchicine (I), Combretastatin A-4 (II), Nocodazole (III), and Vinca alkaloids (IVA–IVB) bind to the colchicine binding site on tubulin, inducing depolymerization of microtubule proteins.^11^ Conversely, Epothilones and Taxanes (V) bind to the taxane binding site, functioning as microtubule polymerization agents or stabilizing agents (Fig. 1). Furthermore, anti-tubulin agents often disrupt tumor vasculature, exhibiting antiangiogenic effects.^8^

**Fig. 2:**
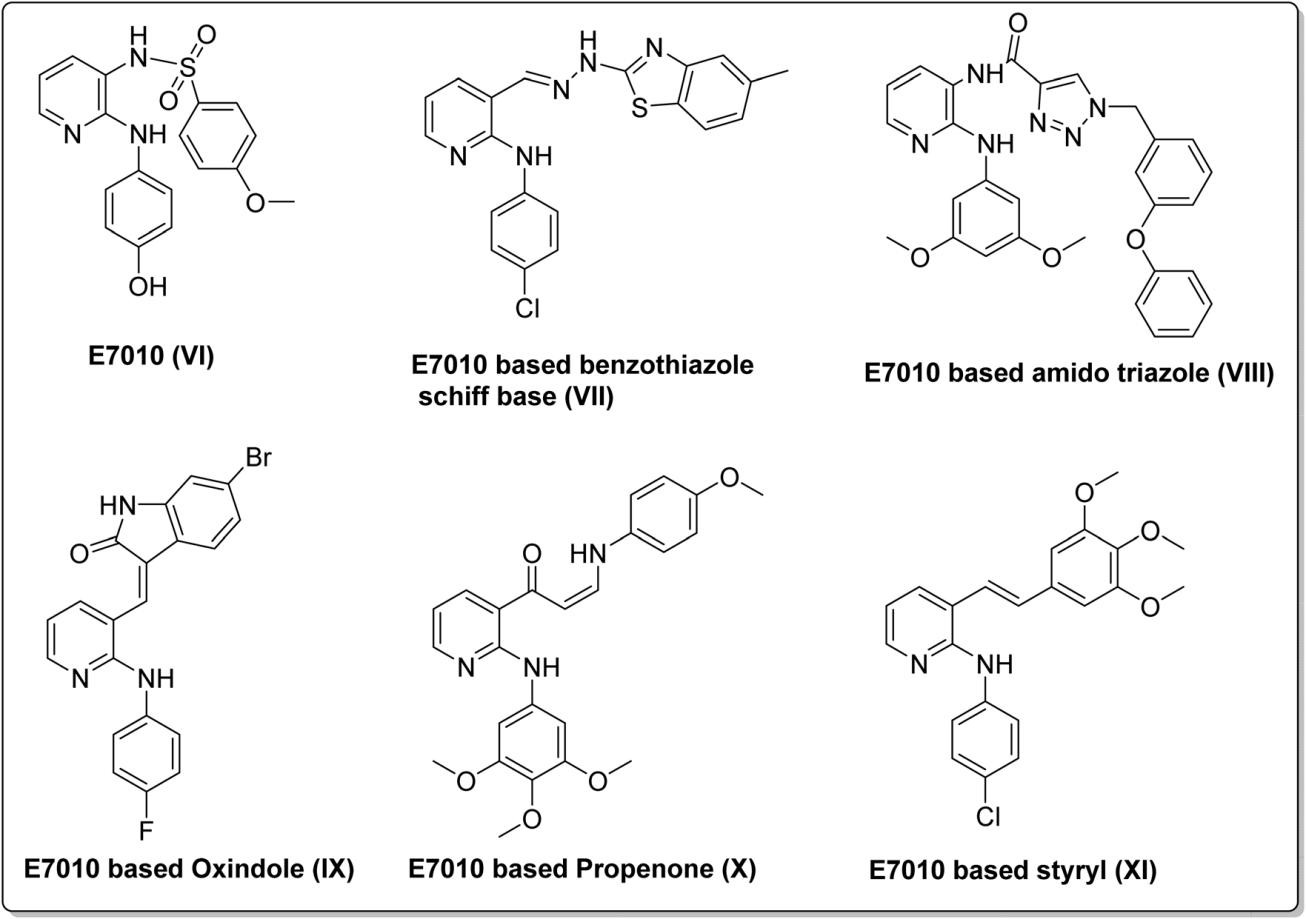
Some examples of promising E7010 leads having anticancer potential.

On the other hand, E7010 (VI), initially synthesized by Hiroshi Yoshino in 1992, stands as a potent orally active inhibitor of tubulin polymerization. This compound exerts its effect by reversibly binding to the colchicine binding site on tubulin, leading to mitotic arrest.^12^ Over the past decade, E7010 has emerged as a highly promising entity, characterized by its broad cytotoxicity range, favorable pharmacokinetic profile, and being less susceptible to the target for the development of multidrug resistance.^13–16^ Furthermore, recent investigations have uncovered the potential of modifying the E7010 compound to yield derivatives with impressive anticancer activity. Our endeavors have been particularly fruitful in harnessing the anti-mitotic capabilities of E7010-based heterocycles for the development of potent antimitotic chemotherapeutics.^17^ Several of the most promising candidates (VII–XI) are illustrated in Fig. 2.

**Fig. 3:**
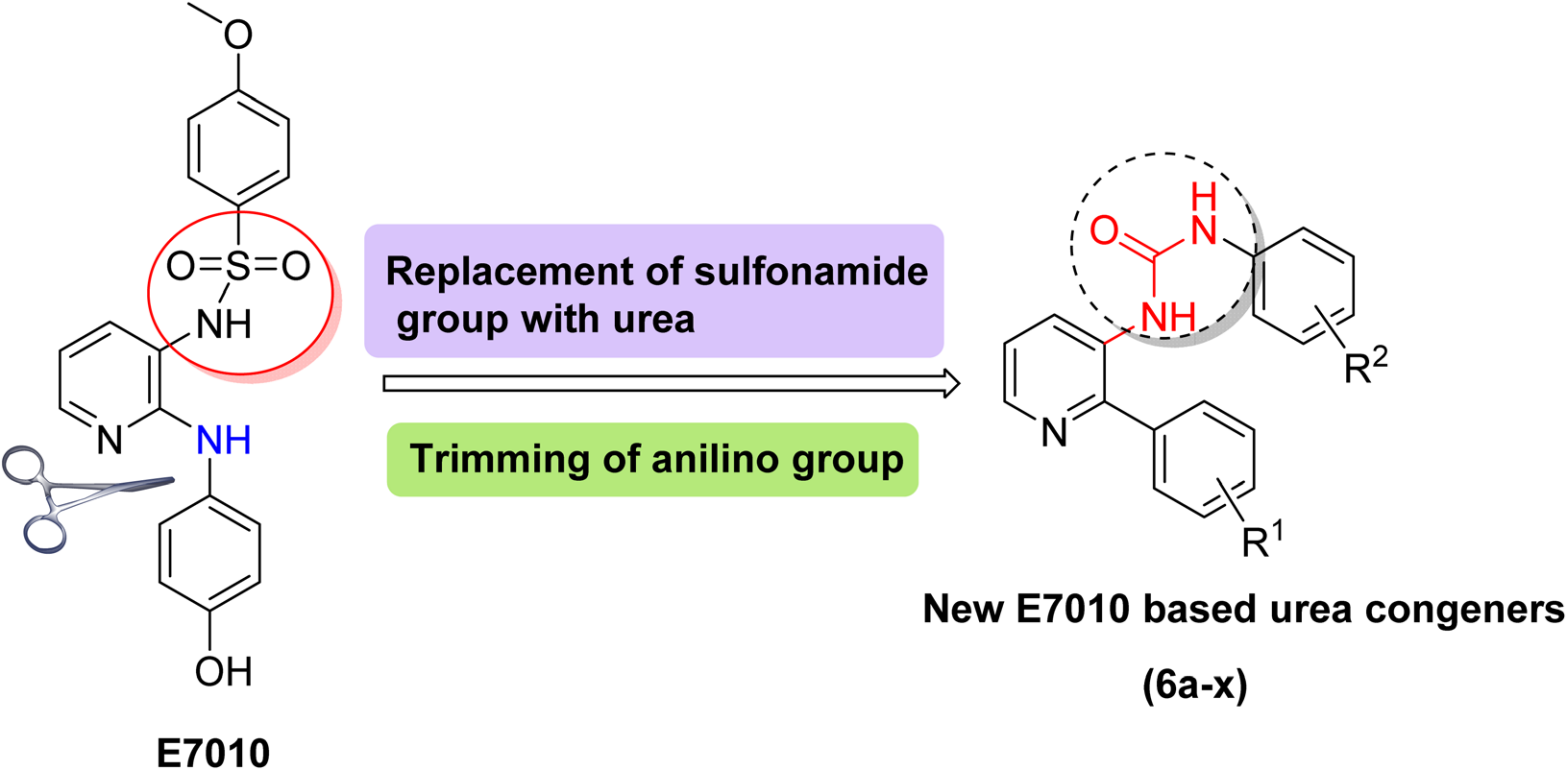
Designed strategy for new E7010 congeners.

Likewise, the potential of including urea functionality into compounds has been explored, and studies have found that they can exhibit significant anticancer activity, potentially *via* tubulin inhibition.^18–21^ Given the remarkable anticancer capabilities observed in E7010 and urea derivatives, and as part of our ongoing efforts to advance the synthesis of novel chemotherapeutic agents,^22^ our focus was directed toward the synthesis of compounds: E7010-linked urea conjugates. This was achieved by replacing the sulphonamide group in E7010 with an aryl urea motif while introducing phenyl substituents in place of the 2-anilino group (as depicted in Fig. 3). Subsequently, these newly designed compounds were evaluated for their anticancer potential against four human cancer cell lines. Furthermore, comprehensive biological investigations were conducted, with particular emphasis on unraveling the mechanistic properties of the potent compound 6q against A549 cells.

## 2 Results and discussion

### 2.1. Chemistry

**Scheme 1:**
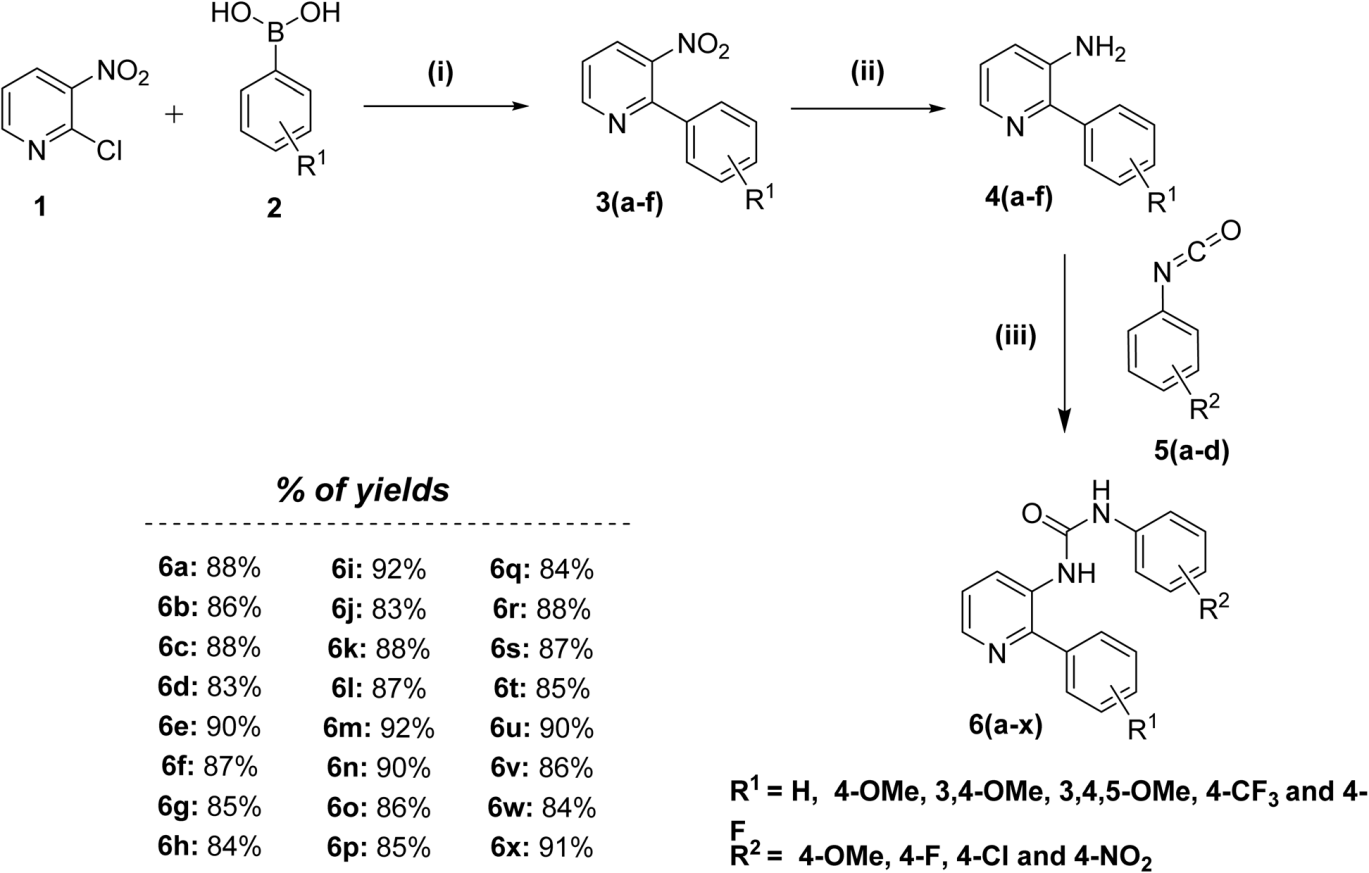
Reagents and conditions: (i) tetrakis(triphenylphosphine)palladium(0), K_2_CO_3_, 1,4-dioxane, 100 °C, 8 h, 80–85%; (ii) H_2_–Pd/C, EtOAc, 0 °C-rt, 8 h, 85–90%; (iii) CHCl_3_, rt, 12 h, 80–92%.

New phenyl-3-(2-phenylpyridin-3-yl)urea congeners (6a–x) were synthesized as described in Scheme 1. The key intermediates were prepared in two sequential steps. Initially, 2-chloro-3-nitropyridine was subjected to Suzuki coupling conditions with substituted boronic acids at 100 °C for 8 h to furnish substituted 3-nitro-2-phenylpyridines (3a–f), which upon reduction in the presence of H_2_–Pd/C in EtOAc at room temperature, yield the pre-final amines (4a–f). Lastly, the obtained pre-final amine (4a–f) intermediates were reacted with substituted phenyl isocyanates (5a–d) to get the desired compounds (6a–x) in good to excellent yields. All the designed and synthesized compounds were appropriately characterized by ^1^H, ^13^C NMR and mass spectrometry.

### 2.2. *In vitro* cell growth inhibition properties

The newly synthesized E7010 congeners (6a–x) were examined for their cytotoxic activity against some cancer cell lines, *viz.*, prostate (DU-145), lung (A549), breast (MDA-MB-231), and cervical (HeLa), by employing the MTT assay.^23^ The IC_50_ values (µM) are represented in Table 1.

**Table 1:** *In vitro* cell growth inhibition properties of (IC_50_^a^ values in µM) of E7010 congeners (6a–x) against human cancer cell lines

Code	R^1^	R^2^	DU-145^b^	A549^c^	MDA-MB-231^d^	HeLa^e^
6a	H	4-OMe	15.13 ± 1.23	9.72 ± 1.21	>25	18.15 ± 2.23
6b	H	4-F	11.5 ± 0.61	14.17 ± 1.84	16.71 ± 2.11	11.37 ± 2.61
6c	H	4-Cl	>25	20.41 ± 3.14	>25	16.81 ± 1.91
6d	H	4-NO_2_	>25	16.74 ± 1.43	12.70 ± 1.83	>25
6e	4-OMe	4-OMe	8.14 ± 0.47	8.82 ± 0.73	9.42 ± 1.12	12.13 ± 1.44
6f	4-OMe	4-F	>25	11.81 ± 0.97	15.62 ± 1.81	18.21 ± 2.51
6g	4-OMe	4-Cl	15.72 ± 1.86	8.46 ± 1.13	>25	16.59 ± 0.42
6h	4-OMe	4-NO_2_	7.47 ± 0.62	9.76 ± 0.63	19.74 ± 2.46	6.95 ± 0.81
6i	3,4- OMe	4-OMe	21.37 ± 3.44	14.36 ± 1.29	11.26 ± 1.79	15.88 ± 2.62
6j	3,4- OMe	4-F	10.41 ± 1.53	12.53 ± 0.89	8.72 ± 1.54	10.92 ± 1.82
6k	3,4- OMe	4-Cl	14.45 ± 1.27	20.28 ± 3.81	>25	17.91 ± 1.57
6l	3,4- OMe	4-NO_2_	6.78 ± 0.84	6.62 ± 0.77	9.34 ± 1.18	12.01 ± 1.78
6m	3,4,5-OMe	4-OMe	>25	19.15 ± 1.67	22.74 ± 3.25	>25
6n	3,4,5-OMe	4-F	15.24 ± 1.62	>25	16.71 ± 2.97	18.32 ± 2.14
6o	3,4,5-OMe	4-Cl	11.93 ± 1.75	>25	>25	>25
6p	3,4,5-OMe	4-NO_2_	13.31 ± 2.11	11.41 ± 0.92	13.40 ± 2.12	15.95 ± 1.33
6q	4-F	4-OMe	3.88 ± 0.57	2.03 ± 0.15	5.79 ± 0.89	8.14 ± 1.12
6r	4-F	4-F	7.32 ± 0.82	8.17 ± 1.02	14.45 ± 2.27	11.45 ± 0.96
6s	4-F	4-Cl	14.47 ± 2.04	15.42 ± 2.11	18.64 ± 2.11	18.16 ± 2.31
6t	4-F	4-NO_2_	6.53 ± 0.73	7.81 ± 1.67	8.91 ± 1.31	10.17 ± 1.36
6u	4-CF_3_	4-OMe	21.17 ± 2.66	>25	21.14 ± 1.79	13.23 ± 1.78
6v	4-CF_3_	4-F	13.67 ± 1.94	18.73 ± 2.54	12.67 ± 2.03	22.06 ± 3.57
6w	4-CF_3_	4-Cl	17.24 ± 1.42	11.42 ± 1.85	19.14 ± 2.74	>25
6x	4-CF_3_	4-NO_2_	8.21 ± 1.13	11.46 ± 2.17	10.51 ± 2.15	19.21 ± 2.71
Nocodazole			2.14 ± 0.19	2.39 ± 0.14	2.13 ± 0.25	3.48 ± 0.52
E7010			2.23 ± 0.17	2.67 ± 0.33	2.51 ± 0.41	2.88 ± 0.24
Cisplatin			1.87 ± 0.21	4.54 ± 0.27	6.35 ± 0.51	3.3 ± 0.1

a IC_50_ values denote the concentrations at which 50% inhibition of the cell growth takes place. Presented data depicts the mean ± standard deviation derived from the three independent studies performed in triplicate.

b DU145 – prostate cancer.

c A549 – lung cancer.

d MDA-MB-231 – breast cancer.

e HeLa – vervical cancer cell lines.

**Fig. 4:**
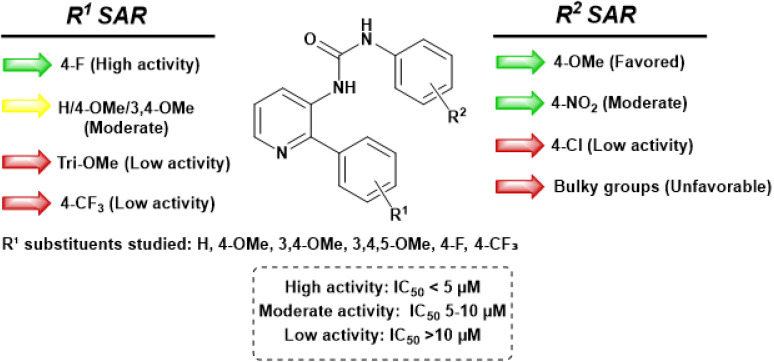
Summarized SAR map showing the influence of R^1^ and R^2^ substituents on anti-proliferative activity. Color coding: green = high (IC_50_ < 5 µM), yellow = moderate (5–10 µM), red = low (>10 µM).

These results suggest that most of the newly prepared molecules exhibited moderate to appreciable cytotoxicity against the screened cell lines. Remarkably, compound 6q (R^1^ = 4-F and R^2^ = 4-OMe) exhibited substantial cancer cell growth inhibition against the A549 lung cancer cell line with an IC_50_ of 2.03 µM, equivalent in potency to the positive controls nocodazole and E7010. Noteworthy inhibitory activities were also observed for compounds 6e, 6h, 6l, 6r, and 6t against the DU-145 and A549 cell lines, with IC_50_ values ranging from 6.5 to 8.2 µM. Furthermore, compounds 6e, 6j, 6l, 6q, and 6t exhibited moderate growth inhibition against the breast cancer cells (MDA-MB-231), having IC_50_ values below 10 µM. It is observed that when the R^1^ substitution is 3,4,5-trimethoxy (6m, 6n, 6o) and 4-trifluoromethyl (6u, 6v, 6w) the cytotoxic activity reduces considerably with most of the R^2^ functional groups. However, they are moderately active, when the R^2^ group is NO_2_ in both the cases (6p and 6x). Thus, to assess selectivity towards cancer cells, the most potent compound, 6q, was tested against non-cancerous HEK-293 cells. Compound 6q, along with the standard E7010, exhibited lower inhibitory effects on HEK-293 cell growth, with IC_50_ values of 10.35 ± 1.12 and 5.18 ± 0.89 µM, respectively, compared to the tested cancer cell lines. These promising preliminary results encouraged us to investigate this compound (6q) for further detailed biological studies (Fig. 4).

### 2.3. Cell cycle analysis

**Fig. 5:**
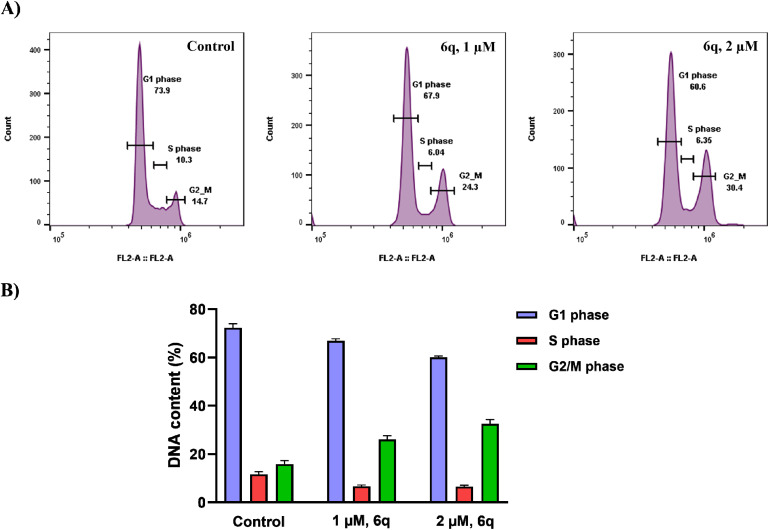
(A) The impact of compound 6q on the cell cycle progression in A549 cells was assessed upon treatment for 48 hours with concentrations of 1 and 2 µM. The cells were then stained with propidium iodide and analyzed by using flow cytometry. (B) Presented data in the bar graph shows the mean ± standard deviation obtained from three different experimental studies.

Most cytotoxic agents have a characteristic feature of arresting specific phases of the cell cycle, inducing apoptosis, or a combination of both, resulting in the exertion of growth-inhibitory effects.^24^ In order to elucidate the mechanism through which compound 6q inhibits cell growth, we conducted a cell cycle analysis on A549 cells. For this purpose, the cells were incubated with the compound 6q at different concentrations, 1 and 2 µM, for 48 h, stained with DNA-binding dye propidium iodide, and were then analyzed by flow cytometry. The results obtained clearly showed the compound's ability to arrest the cell cycle in a concentration-dependent manner (Fig. 5). Notably, treatment with compound 6q led to the accumulation of 24.3% and 30.4% of A549 cells in the G2/M phase after exposure to 1 and 2 µM concentrations, respectively, as compared to the untreated control (14.7%).

### 2.4. Inhibition of tubulin polymerization

**Fig. 6:**
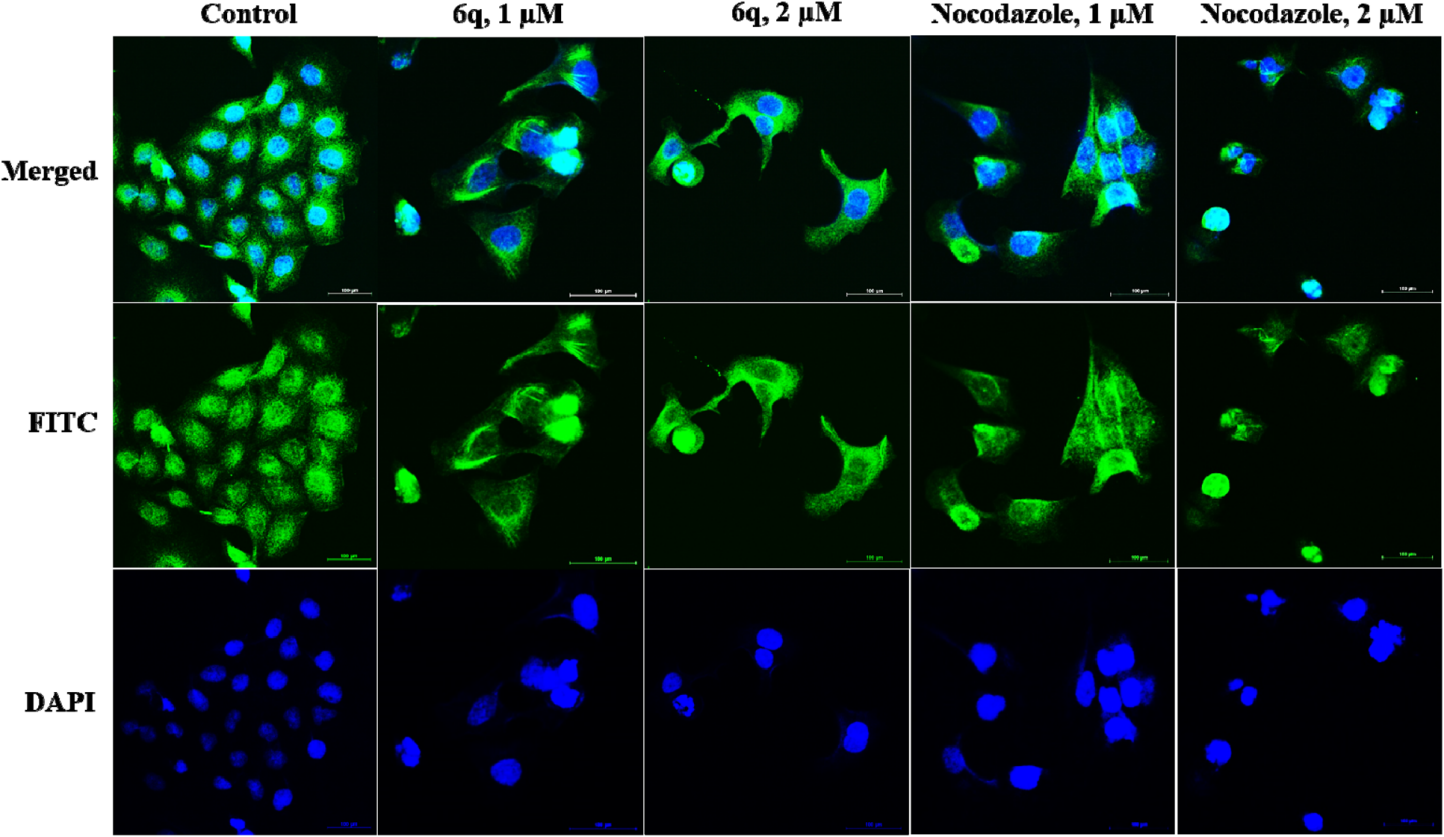
Immunohistochemistry examination of compound effects on microtubule networks. Involved incubating A549 cells with 6q and nocodazole at concentrations of 1 and 2 µM, respectively, for 48 hours. The images were captured with a confocal microscope. The scale bar represents 100 µm.

Tubulin is a central component of the cytoskeletal network and is essential for various cellular functions, including mitosis. Given that compound 6q induced G2/M phase arrest in the cell cycle analysis, it is plausible that it could impede tubulin polymerization, thereby affecting cellular processes.^25^ To validate this effect, the variations in the microtubule network in A549 cells by immunohistochemistry studies were incubated with this compound for 48 h with individual concentrations of 1 and 2 µM, and the data obtained was compared with the untreated control and nocodazole as positive control. The results confirmed a well-organized microtubular network in untreated cells, whereas cells treated with 1 and 2 µM of 6q showed disorganized microtubules (Fig. 6), confirming the inhibition of tubulin polymerization. Additionally, the positive control, nocodazole, exhibits the expected pattern of disrupted microtubule organization.

### 2.5. Molecular docking studies

**Fig. 7:**
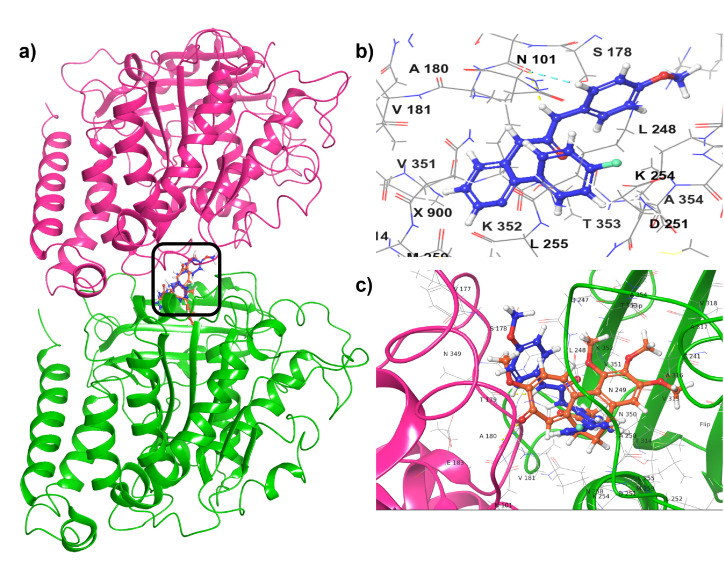
(a) Superimposition of 6q (ball and stick model-blue color) and colchicine (ball and stick model-orange color) at the predicted binding pocket of a/b tubulin. (b) Predicted binding interactions at the active pocket of a/b tubulin with 6q. (c) Superimposition of 6q (blue color) and colchicine (orange color) to the tubulin's colchicine binding site.

Docking studies were conducted to examine the mode of binding and type of interactions between compound 6q and α/β-tubulin employing the Glide docking module within the Schrödinger suite.^26^ The α/β-tubulin's crystal structure coordinates were obtained from the RCSB protein data bank (PDB ID: 3E22). As shown in Fig. 7, the molecular docking results suggest that the best conformation of 6q is accommodated well within the tubulin's active site. This molecule exhibited two hydrogen bond interactions with Thr179 and Asn101, which are the crucial active site residues. Specifically, the nitrogen atom of the urea functional group established an interaction of hydrogen bond with the backbone carboxylic acid functionality of Thr179, with a separation of 3.18 Å. Furthermore, the urea moieties oxygen atom is predicted to be engaged in a hydrogen bond interaction with the amino functionality of Asn101 with a separation of 3.79 Å. In addition to these hydrogen bond interactions, it is also engaged in many interactions of hydrophobic nature with the α/β-tubulin's key amino acid residues, encompassing Val181, Ala180, Leu248, Leu255, Met259, Val315, Ala250, Ala316 and Tyr224. Collectively, these findings provide compelling evidence that this compound exhibits an intense affinity for the colchicine binding site of the tubulin protein.

### 2.6. Colony forming assay

**Fig. 8:**
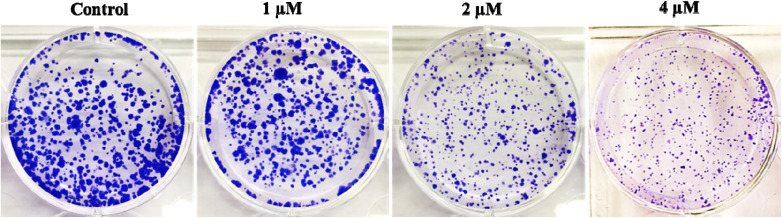
Effect of 6q (1, 2, and 4 µM) on clonogenicity of A549 cancer cells. Representative images from the colony formation assay showing the untreated control and treatment groups. Colonies were stained with crystal violet after 14 days.

The long-term cytotoxic and growth-inhibiting capabilities of compound 6q were investigated using a clonogenic cell survival assay. This assay assesses the ability of individual cells to form colonies over an extended period.^25^ The untreated control displayed colonies, with dense and large, blue-stained clusters, indicating a strong capacity for proliferative growth. Treatment with 1 µM resulted in a relatively similar number of colonies compared to the control, but with visibly smaller colonies, indicating partial suppression of proliferative capacity. A significant decrease in colony density, with primarily small-sized colonies, was observed when the dose was increased to 2 µM. This suggests that higher treatment significantly hinders the ability of surviving cells to divide multiple times. Increasing the dose to 4 µM, A549 cells displayed the fewest colonies, which were very small and faintly stained, indicating a marked cytotoxic or cytostatic effect, Fig. 8. Overall, results indicate that the treatment with this compound reduced clonogenic survival in a dose-dependent manner. This reduction could be attributed to several mechanisms, including induction of apoptosis, cell cycle arrest, or persistent DNA damage that prevents cell proliferation.

### 2.7. Wound healing assay (migration assay)

**Fig. 9:**
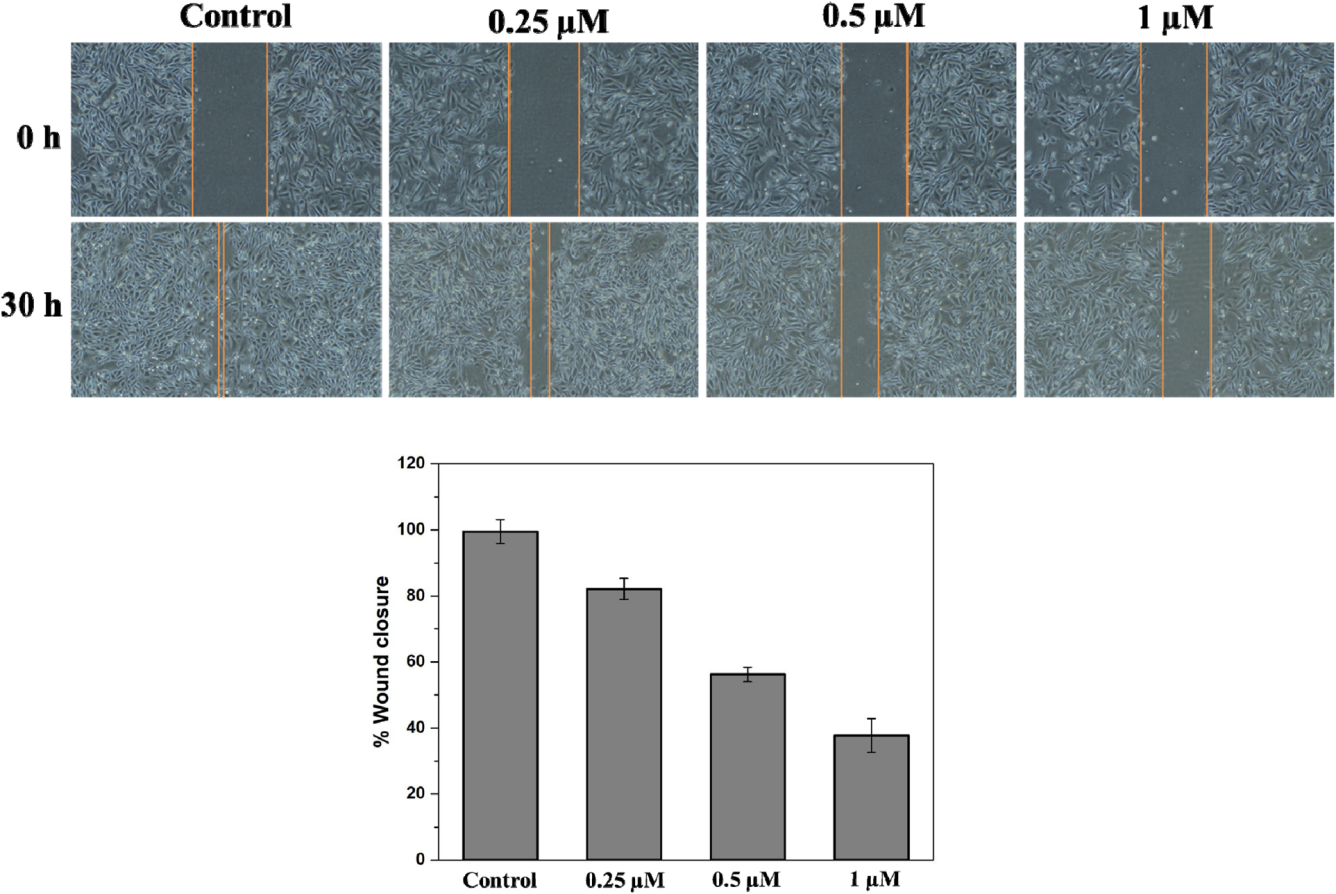
HUVECs cells were treated with compound 6q (0.25, 0.5, and 1 µM), and scratches were created artificially using a sterile pipette (200 µL). Representative images were acquired by employing a phase contrast microscopy at both 0 and 30 hours. The percentage of wound closure was analyzed through ImageJ. The mean ± standard deviation results were obtained from three independent experimental studies, which is illustrated in the bar graph.

Cancer cell migration plays a pivotal role in the metastasis process, while endothelial cell migration is a fundamental requirement for angiogenesis, a crucial step in tumor growth.^25^ Therefore, we evaluated the antimigratory potential of compound 6q using a wound healing assay. In this experiment, wounds were created in confluent monolayers of endothelial HUVEC cells and subsequently treated these cells with various subtoxic concentrations of this molecule. The cells start migrating within this wounded area, which was then monitored employing a phase-contrast microscope. After a 30-hour observation period, the results revealed a concentration-dependent inhibition of HUVEC cell migration induced by this compound. In the untreated control group, the wounds had nearly closed completely (99.5%). In stark contrast, treatment with this molecule at 0.25 µM resulted in a notable slowdown of cell migration, reducing wound closure to 82.15%. Notably, at 1 µM concentration, it significantly inhibited cell migration, with only 37.7% wound closure observed (Fig. 9).

### 2.8. Apoptosis

Apoptosis is the programmed cell death that occurs due to severe, irreversible damage to the cell's prominent organelles.^27^ Most of the cytotoxic agents act by inducing apoptosis. Therefore, it was considered essential to understand the apoptosis-inducing property of this molecule (6q) on A549 (lung cancer) cells.

#### 2.8.1. Hoechst staining

**Fig. 10:**
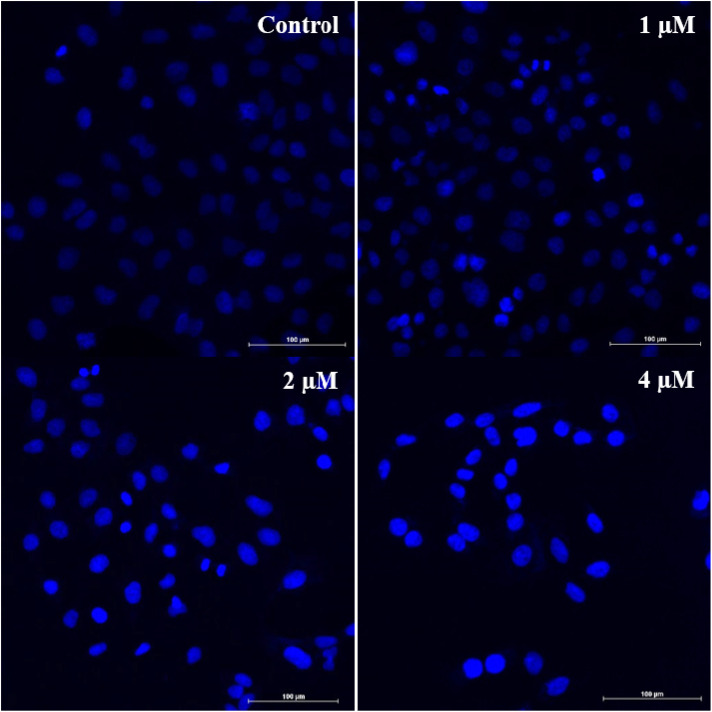
The effect of compounds 6q on nuclear morphological changes in A549 cells was examined following 48 h treatment and subsequent staining with Hoechst. The images were acquired using fluorescence microscope. The scale bar represents 100 µm.

To visualize the various morphological changes that could be induced by this new molecule (6q) in A549 cells, a Hoechst nuclear staining experiment was conducted. A549 cells were subjected to treatment of this compound for 24 hours with varying concentrations (1, 2, and 4 µM) and subsequently Hoechst 33242 was used for its staining.^27^ As depicted in Fig. 10, control cells did not display any notable morphological changes. In contrast, the treated cells exhibited characteristic apoptotic features, including highly condensed nuclei that appeared brightly stained. Notably, these apoptotic morphological changes occurred in a concentration-dependent manner. These findings strongly suggest that it can induce apoptosis in A549 cells.

#### 2.8.2. Effect on mitochondrial membrane potential (Δ*Ψ*_m_)

**Fig. 11:**
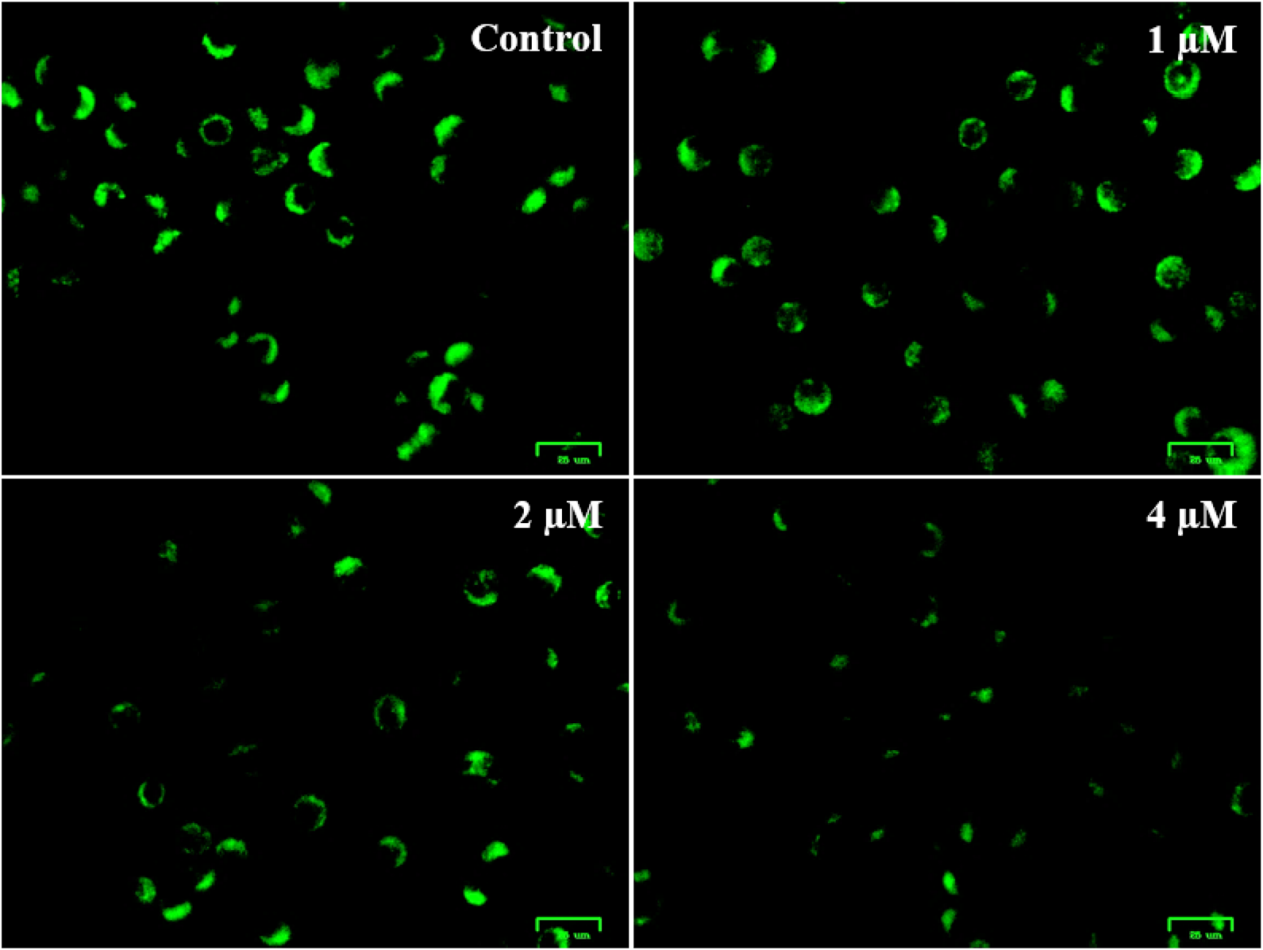
Compound 6q (at concentrations of 1, 2, and 4 µM) induced changes in mitochondrial membrane potential (Δ*Ψ*_m_) in A549 cells, as analyzed through rhodamine 123 staining. Images were acquired using a fluorescence microscope, with the scale bar indicating 25 µm.

In the process of apoptosis, mitochondria are known to play a crucial role, which is commonly linked with the depolarization of the mitochondrial membrane potential (Δ*Ψ*_m_).^28^ To investigate whether depolarization of mitochondria was involved in apoptosis induced by this compound (6q), the changes in Δ*Ψ*_m_ were measured by staining with rhodamine-123. Mitochondria that remain normal can retain the stain rodamine-123 (strong green fluorescence), whereas the disruptions in Δ*Ψ*_m_ lead to reduced fluorescence due to loss of rodamine-123 retention. A549 cells were treated for 48 h with this molecule of different concentrations (1, 2, and 4 µM) and subsequently stained with rodamine-123, and the fluorescence was imaged using fluorescence microscopy. The results from Fig. 11 indicate that the cells treated with the compound showed reduced green fluorescence compared to the control, indicating disruption of mitochondrial membrane potential. This decrease was substantial, particularly at higher concentrations. These results demonstrate that it induces apoptosis through a dose-dependent change in Δ*Ψ*_m_.

#### 2.8.3. Assay of annexin-V FITC

**Fig. 12:**
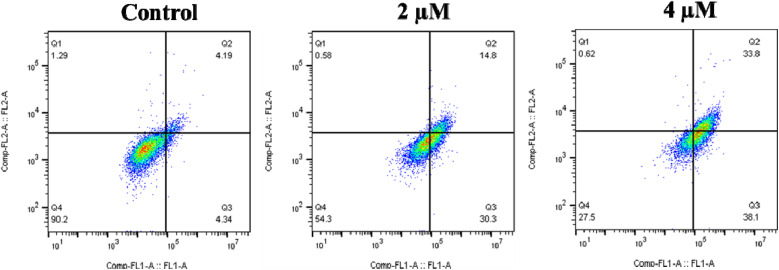
Treatment of A549 cells with compound 6q (1.5 and 3 µM) and Annexin V-FITC/PI staining was carried out, and then analyzed using flow cytometer for apoptosis. The Annexin V-FITC and/or propidium iodide positive cells percentage is shown inside the quadrants. It is seen that the cells in the upper left quadrant (Q1-UL: V−/PI+): are necrotic cells; while the upper right quadrant (Q2-UR: V+/PI+): are late apoptotic cells; whereas the lower right quadrant (Q3-LR: V+/PI−): are early apoptotic cells and the lower left quadrant (Q4-LL: V−/PI−): are live cells.

Annexin V FITC/PI (AV/PI) double staining assay was employed to evaluate the apoptotic effect of compound 6q on A549 cells. This assay determines the percentage of live cells (annexin-V−/PI−), early apoptotic cells (annexin-V+/PI−), late apoptotic cells (annexin-V+/PI+), and necrotic cells (annexin-V−/PI+). In this study, A549 cells were treated with different concentrations of this molecule (2 µM and 4 µM) for 48 h to examine the apoptotic effect.^23^ The results indicate that it affects the cells in a dose-dependent manner and resulted in 45.1% and 71.9% of apoptosis at 2 and 4 µM, respectively (Fig. 12).

## 3 Conclusion

A series of new 1-phenyl-3-(2-phenylpyridin-3-yl)urea congeners were designed, synthesized, and examined for their cytotoxic activities. Among the synthesized compounds, compound 6q showed significant growth inhibition towards some tumor cell lines with IC50 values ranging from 2.03 to 8.14 µM. It has exhibited potent growth inhibition with an IC_50_ of 2.03 µM towards A549 cells. Mechanism studies indicated that it arrested the A549 cells in the G2/M phase of the cell cycle and inhibited tubulin assembly, thereby disrupting the microtubule dynamics. Furthermore, molecular docking of this compound offered supporting insights into binding modes and protein-ligand interactions at the colchicine binding pocket of tubulin. Additionally, the induction of apoptosis was confirmed through experiments involving Hoechst staining, assessment of mitochondrial membrane potential (Δ*Ψ*_m_), and assays on Annexin V-FITC in A549 cells. Based on these studies, it can be concluded that this molecule exhibits significant cytotoxic activity by inhibiting tubulin polymerization and inducing apoptosis. Therefore, it appears promising for its further detailed exploration and could be considered as a potential lead for the development of newer chemotherapeutic agents.

## 4 Experimental section

### 4.1. Chemistry

Starting materials and reagents were purchased from Alfa Aesar and Aldrich and used without further purification. Reactions were monitored by TLC analysis using Merck 9385 aluminium plates pre-coated with silica gel containing F254 indicator, which were visualised using UV light. Column chromatography was performed using Merck silica gel of 60–120 mesh with hexane and ethyl acetate as eluents. Melting points were determined on an electrothermal melting point apparatus and are uncorrected. Nuclear Magnetic Resonance spectra (^1^H and ^13^C) were recorded on 300 MHz Bruker spectrometer, using TMS as an internal reference. The chemical shifts values are expressed in parts per million (ppm) and coupling constants (*J*) in Hertz. Splitting patterns of multiplicities are designated as: s, singlet; d, doublet; t, triplet; q, quartet; dd, doublet of doublet; m, multiplet. High-resolution mass spectra were obtained using an ESI-QTOF mass spectrometer (70 eV).

#### 4.1.1. Synthesis of 3-nitro-2-phenylpyridines (3a–f)

To a solution of 2-chloro-3-nitropyridine (1 equiv.) in 1,4-dioxane (20 mL) and water (2 mL), K_2_CO_3_ (1.5 equiv.) was added, followed by Pd(PPh_3_)_4_ (3 mol%) and appropriate arylboronic acid (1.2 equiv.) under argon in a 50 mL two-necked flask. The mixture was heated to reflux for 10 hours, then cooled to room temperature and diluted with water (20 mL). The aqueous layer was adjusted to a pH of 7–8 using a saturated aqueous K_2_CO_3_ solution and extracted three times with EtOAc. The organic phase was washed with water and brine, dried over sodium sulfate (Na_2_SO_4_), filtered, and then concentrated. The crude products were purified through column chromatography to yield the intermediates, 3a–f (80–85%).

#### 4.1.2. Synthesis of 2-phenylpyridin-3-amines (4a–f)

3-Nitro-2-phenylpyridine (3a–f, 2.70 mmol) and Pd/C 10% (144 mg, 0.13 mmol) were added to ethyl acetate (40 mL) under a nitrogen atmosphere at 0 °C in a dry, sealed tube fitted with a H_2_ balloon. The mixture was stirred for 4 hours at room temperature. After filtration through Celite, the solvent was removed under reduced pressure, and the crude mixture was purified by chromatography (silica gel, PE–EtOAc, 4 : 1) to afford compounds 4a–f with yields of 85–90%. The obtained product's NMR data matched those reported in the literature.^29^

#### 4.1.3. General procedure for the synthesis of 1-phenyl-3-(2-phenylpyridin-3-yl)urea congeners (6a–x)

To a mixture of amines (4a–f, 1 equiv.) in dioxane, substituted aryl isocyanates (5a–d, 1 equiv.) were added at 100 °C and stirred until the complete consumption of the starting materials was confirmed by TLC (up to 12 hours). The reaction mixture was then quenched with water and extracted three times with ethyl acetate (25 mL each). The organic phase was washed with water and brine, dried over sodium sulfate (Na_2_SO_4_), and filtered. The organic layer was concentrated under vacuum, and the residue was chromatographed on silica gel (elution with hexane/EtOAc) to yield the 1-phenyl-3-(2-phenylpyridin-3-yl)urea congeners (6a–x) in excellent yields (75–90%).

##### 4.1.3.1. 1-(4-Methoxyphenyl)-3-(2-phenylpyridin-3-yl)urea (6a)

White solid, yield 88%, Mp: 172–174 °C; ^1^H NMR (300 MHz, CDCl_3_ + DMSO): *δ* 8.62 (s, 1H), 8.49 (d, *J* = 8.4 Hz, 1H), 8.36–8.30 (m, 1H), 7.69–7.63 (m, 3H), 7.55–7.43 (m, 3H), 7.32 (d, *J* = 9.0 Hz, 2H), 7.26 (dd, *J* = 8.3, 4.6 Hz, 1H), 6.82 (d, *J* = 9.0 Hz, 2H), 3.78 (s, 3H). ^13^C NMR (75 MHz, CDCl_3_ + DMSO) *δ* 154.76, 153.03, 148.30, 142.97, 137.85, 132.92, 131.92, 128.96, 128.74, 128.29, 127.92, 122.12, 120.32, 113.60, 54.97. MS (ESI): *m*/*z* 320 [M + H]^+^. HRMS (ESI) calcd for C_19_H_17_N_3_O_2_ [M + H]^+^, 320.1399; found, 320.1392.

##### 4.1.3.2. 1-(4-Fluorophenyl)-3-(2-phenylpyridin-3-yl)urea (6b)

White solid, yield 86%, Mp: 181–183 °C; ^1^H NMR (300 MHz, CDCl_3_ + DMSO): *δ* 8.85 (s, 1H), 8.47 (dd, *J* = 8.4, 1.3 Hz, 1H), 8.35 (d, *J* = 4.0 Hz, 1H), 7.74 (s, 1H), 7.70–7.64 (m, 2H), 7.56–7.44 (m, 3H), 7.40 (dt, *J* = 6.9, 4.0 Hz, 2H), 7.27 (dd, *J* = 8.3, 4.6 Hz, 1H), 6.96 (t, *J* = 8.7 Hz, 2H). ^13^C NMR (101 MHz, CDCl_3_) *δ* 160.06 (d, *J*_CF_ = 245 Hz), 153.30, 149.16, 144.15, 137.36, 133.22, 132.69, 129.12, 128.84 (d, *J*_CF_ = 14.0 Hz), 124.26, 123.17, 116.20 (d, *J*_CF_ = 22.22 Hz). MS (ESI): *m*/*z* 308 [M + H]^+^. HRMS (ESI) calcd for C_18_H_15_FN_3_O [M + H]^+^, 308.1199; found, 308.1193.

##### 4.1.3.3. 1-(4-Chlorophenyl)-3-(2-phenylpyridin-3-yl)urea (6c)

White solid, yield 88%, Mp: 199–201 °C; ^1^H NMR (300 MHz, CDCl_3_ + DMSO): *δ* 8.94 (s, 1H), 8.46 (dd, *J* = 8.3, 1.3 Hz, 1H), 8.35 (dd, *J* = 4.6, 1.3 Hz, 1H), 7.79 (s, 1H), 7.70–7.64 (m, 2H), 7.52 (dd, *J* = 14.8, 7.1 Hz, 3H), 7.40 (d, *J* = 8.8 Hz, 2H), 7.30–7.24 (m, 1H), 7.21 (d, *J* = 8.8 Hz, 2H). ^13^C NMR (75 MHz, CDCl_3_ + DMSO) *δ* 152.53, 148.51, 143.29, 137.79, 132.50, 129.21, 128.72, 128.25, 128.11, 127.96, 126.12, 122.03, 119.33. MS (ESI): *m*/*z* 324 [M + H]^+^. HRMS (ESI) calcd for C_18_H_15_ClN_3_O [M + H]^+^, 324.0903; found, 324.0898.

##### 4.1.3.4. 1-(4-Nitrophenyl)-3-(2-phenylpyridin-3-yl)urea (6d)

Yellow solid, yield 83%, Mp: 280–282 °C; ^1^H NMR (300 MHz, CDCl_3_ + DMSO): *δ* 9.51 (s, 1H), 8.45 (d, *J* = 8.3 Hz, 1H), 8.39 (dd, *J* = 4.6, 1.3 Hz, 1H), 8.14 (d, *J* = 9.2 Hz, 2H), 7.98 (s, 1H), 7.70–7.66 (m, 2H), 7.64–7.60 (m, 2H), 7.58–7.44 (m, 3H), 7.31 (dd, *J* = 8.3, 4.6 Hz, 1H). MS (ESI): *m*/*z* 335 [M + H]^+^. HRMS (ESI) calcd for C_18_H_15_N_4_O_3_ [M + H]^+^, 335.1144; found, 335.1138.

##### 4.1.3.5. 1-(4-Methoxyphenyl)-3-(2-(4-methoxyphenyl)pyridin-3-yl)urea (6e)

White solid, yield 90%, Mp: 195–197 °C; ^1^H NMR (300 MHz, CDCl_3_ + DMSO): *δ* 8.64 (s, 1H), 8.47 (d, *J* = 7.0 Hz, 1H), 8.30 (s, 1H), 7.60 (dd, *J* = 20.2, 13.1 Hz, 4H), 7.32 (d, *J* = 7.4 Hz, 2H), 6.93 (dd, *J* = 64.8, 7.1 Hz, 4H), 3.87 (s, 3H), 3.78 (s, 3H). MS (ESI): *m*/*z* 350 [M + H]^+^. HRMS (ESI) calcd for C_20_H_20_N_3_O_3_ [M + H]^+^, 350.1505; found, 350.1499.

##### 4.1.3.6. 1-(4-Fluorophenyl)-3-(2-(4-methoxyphenyl)pyridin-3-yl)urea (6f)

White solid, yield 87%, Mp: 201–203 °C; ^1^H NMR (300 MHz, CDCl_3_ + DMSO): *δ* 8.81 (s, 1H), 8.47 (dd, *J* = 8.3, 1.4 Hz, 1H), 8.34 (dd, *J* = 4.6, 1.4 Hz, 1H), 7.70 (s, 1H), 7.66–7.60 (m, 2H), 7.44–7.37 (m, 2H), 7.24 (dd, *J* = 8.3, 4.6 Hz, 1H), 7.06 (d, *J* = 8.8 Hz, 2H), 7.02–6.92 (m, 2H), 3.89 (s, 3H). ^13^C NMR (75 MHz, CDCl_3_ + DMSO) *δ* 157.81 (d, *J*_CF_ = 235.5 Hz), 156.25, 152.99, 148.33, 143.20, 135.15, 132.71, 130.20, 128.97, 121.87, 120.01 (d, *J*_CF_ = 7.5 Hz), 114.91 (d, *J*_CF_ = 22.5 Hz), 113.81, 55.00. MS (ESI): *m*/*z* 338 [M + H]^+^. HRMS (ESI) calcd for C_19_H_17_FN_3_O_2_ [M + H]^+^, 338.1305; found, 338.1129.

##### 4.1.3.7. 1-(4-Chlorophenyl)-3-(2-(4-methoxyphenyl)pyridin-3-yl)urea (6g)

White solid, yield 85%, Mp: 217–219 °C; ^1^H NMR (300 MHz, CDCl_3_ + DMSO): *δ* 8.94 (s, 1H), 8.44 (dd, *J* = 8.3, 1.3 Hz, 1H), 8.35–8.30 (m, 1H), 7.76 (s, 1H), 7.61 (d, *J* = 8.7 Hz, 2H), 7.41 (d, *J* = 8.9 Hz, 2H), 7.26–7.18 (m, 3H), 7.05 (d, *J* = 8.7 Hz, 2H), 3.87 (s, 3H). ^13^C NMR (75 MHz, CDCl_3_ + DMSO): *δ* 159.24, 152.61, 148.22, 143.15, 137.74, 132.42, 130.06, 128.90, 128.14, 126.32, 121.71, 119.36, 113.67, 54.85. MS (ESI): *m*/*z* 354 [M + H]^+^. HRMS (ESI) calcd for C_19_H_17_ClN_3_O_2_ [M + H]^+^, 354.1009; found, 354.1003.

##### 4.1.3.8. 1-(2-(4-Methoxyphenyl)pyridin-3-yl)-3-(4-nitrophenyl)urea (6h)

Yellow solid, yield 84%, Mp: 238–240 °C; ^1^H NMR (300 MHz, CDCl_3_ + DMSO): *δ* 9.51 (s, 1H), 8.42 (d, *J* = 8.2 Hz, 1H), 8.37 (d, *J* = 4.4 Hz, 1H), 8.15 (d, *J* = 9.1 Hz, 2H), 7.95 (s, 1H), 7.65 (dd, *J* = 16.1, 9.1 Hz, 5H), 7.26 (dd, *J* = 8.2, 4.6 Hz, 1H), 7.06 (d, *J* = 8.6 Hz, 2H), 3.88 (s, 3H). ^13^C NMR (75 MHz, CDCl_3_ + DMSO): *δ* 159.32, 152.03, 148.65, 145.72, 143.69, 141.11, 131.84, 130.08, 129.31, 125.75, 124.46, 121.68, 117.04, 113.69, 112.22, 54.87. MS (ESI): *m*/*z* 365 [M + H]^+^. HRMS (ESI) calcd for C_19_H_17_N_4_O_4_ [M + H]^+^, 365.1250; found, 365.1244.

##### 4.1.3.9. 1-(2-(3,4-Dimethoxyphenyl)pyridin-3-yl)-3-(4-methoxyphenyl)urea (6i)

White solid, yield 90%, Mp: 198–200 °C; ^1^H NMR (300 MHz, CDCl_3_ + DMSO): *δ* 8.57 (s, 1H), 8.52 (d, *J* = 8.3 Hz, 1H), 8.31 (d, *J* = 3.4 Hz, 1H), 7.61 (s, 1H), 7.31 (d, *J* = 8.9 Hz, 2H), 7.26–7.17 (m, 3H), 7.00 (d, *J* = 8.2 Hz, 1H), 6.82 (d, *J* = 8.9 Hz, 2H), 3.94 (s, 3H), 3.91 (s, 3H), 3.78 (s, 3H). ^13^C NMR (101 MHz, CDCl_3_): *δ* 156.96, 153.56, 148.89, 148.77, 147.96, 143.09, 133.39, 130.38, 130.19, 127.24, 123.82, 122.92, 121.07, 114.54, 111.63, 110.93, 55.66, 55.61, 55.48. MS (ESI): *m*/*z* 380 [M + H]^+^. HRMS (ESI) calcd for C_21_H_22_N_3_O_4_ [M + H]^+^, 380.1610; found, 380.1610.

##### 4.1.3.10. 1-(2-(3,4-Dimethoxyphenyl)pyridin-3-yl)-3-(4-fluorophenyl)urea (6j)

White solid, yield 83%, Mp: 192–194 °C; ^1^H NMR (300 MHz, CDCl_3_ + DMSO): *δ* 8.90 (s, 1H), 8.48 (d, *J* = 8.3 Hz, 1H), 8.32 (d, *J* = 3.4 Hz, 1H), 7.71 (s, 1H), 7.40 (dd, *J* = 8.9, 4.8 Hz, 2H), 7.27–7.18 (m, 3H), 7.07–7.01 (m, 1H), 6.97 (t, *J* = 8.7 Hz, 2H), 3.94 (d, *J* = 5.2 Hz, 3H), 3.92 (s, 3H). ^13^C NMR (75 MHz, CDCl_3_ + DMSO): *δ* 157.72 (d, *J*_CF_ = 238.5 Hz), 152.83, 148.62, 148.42, 147.99, 142.91, 134.99, 132.65, 130.25, 128.74, 121.84, 121.32, 119.88 (d, *J*_CF_ = 7.5 Hz), 114.76 (d, *J*_CF_ = 21.75 Hz), 111.95, 110.95, 55.48, 55.36. MS (ESI): *m*/*z* 368 [M + H]^+^. HRMS (ESI) calcd for C_20_H_19_FN_3_O_3_ [M + H]^+^, 368.1410; found, 368.1405.

##### 4.1.3.11. 1-(4-Chlorophenyl)-3-(2-(3,4-dimethoxyphenyl)pyridin-3-yl)urea (6k)

White solid, yield 88%, Mp: 208–210 °C; ^1^H NMR (300 MHz, CDCl_3_ + DMSO): *δ* 8.92 (s, 1H), 8.50 (d, *J* = 8.2 Hz, 1H), 8.34 (d, *J* = 3.6 Hz, 1H), 7.72 (s, 1H), 7.40 (d, *J* = 8.7 Hz, 2H), 7.29–7.16 (m, 5H), 7.03 (d, *J* = 8.1 Hz, 1H), 3.94 (s, 3H), 3.92 (s, 3H). ^13^C NMR (75 MHz, CDCl_3_ + DMSO): *δ* 172.55, 152.61, 148.67, 148.43, 148.10, 143.02, 137.78, 132.55, 130.17, 128.90, 128.17, 126.32, 121.84, 121.36, 119.39, 112.00, 110.99, 55.50, 55.38. MS (ESI): *m*/*z* 384 [M + H]^+^. HRMS (ESI) calcd for C_20_H_19_ClN_3_O_3_ [M + H]^+^, 384.1115; found, 384.1109.

##### 4.1.3.12. 1-(2-(3,4-Dimethoxyphenyl)pyridin-3-yl)-3-(4-nitrophenyl)urea (6l)

Yellow solid, yield 87%, Mp: 259–261 °C; ^1^H NMR (300 MHz, CDCl_3_ + DMSO): *δ* 9.54 (s, 1H), 8.52–8.44 (m, 1H), 8.38 (d, *J* = 3.6 Hz, 1H), 8.15 (d, *J* = 9.2 Hz, 2H), 7.93 (s, 1H), 7.62 (d, *J* = 9.2 Hz, 2H), 7.31–7.20 (m, 3H), 7.05 (d, *J* = 8.2 Hz, 1H), 3.95 (s, 3H), 3.92 (s, 3H). ^13^C NMR (75 MHz, CDCl_3_ + DMSO): *δ* 152.09, 148.75, 148.48, 148.30, 145.56, 143.59, 141.29, 131.97, 130.06, 129.02, 124.52, 121.89, 121.41, 117.03, 111.97, 110.97, 55.52, 55.41. MS (ESI): *m*/*z* 395 [M + H]^+^. HRMS (ESI) calcd for C_20_H_19_N_4_O_5_ [M + H]^+^, 395.1355; found, 395.1352.

##### 4.1.3.13. 1-(4-Methoxyphenyl)-3-(2-(3,4,5-trimethoxyphenyl)pyridin-3-yl)urea (6m)

Light brown solid, yield 92%, Mp: 209–211 °C; ^1^H NMR (300 MHz, DMSO): *δ* 8.79 (s, 1H), 8.54 (d, *J* = 8.3 Hz, 1H), 8.31 (d, *J* = 4.5 Hz, 1H), 7.68 (s, 1H), 7.53 (d, *J* = 2.8 Hz, 1H), 7.31 (t, *J* = 8.1 Hz, 2H), 7.26 (dd, *J* = 8.2, 4.7 Hz, 1H), 6.87 (s, 2H), 6.82 (d, *J* = 8.7 Hz, 2H), 3.89 (d, *J* = 2.3 Hz, 6H), 3.89 (s, 3H), 3.78 (s, 3H). ^13^C NMR (75 MHz, CDCl_3_ + DMSO) *δ* 154.89, 153.08, 152.84, 147.82, 142.58, 137.35, 133.01, 131.76, 128.54, 122.21, 120.51, 113.62, 105.69, 60.30, 55.58, 54.96. MS (ESI): *m*/*z* 410 [M + H]^+^. HRMS (ESI) calcd for C_22_H_24_N_3_O_5_ [M + H]^+^, 410.1716; found, 410.1710.

##### 4.1.3.14. 1-(4-Fluorophenyl)-3-(2-(3,4,5-trimethoxyphenyl)pyridin-3-yl)urea (6n)

Light brown solid, yield 90%, Mp: 222–224 °C; ^1^H NMR (300 MHz, CDCl_3_ + DMSO): *δ* 9.00 (s, 1H), 8.54 (d, *J* = 8.3 Hz, 1H), 8.33 (d, *J* = 4.6 Hz, 1H), 7.74 (s, 1H), 7.40 (dd, *J* = 8.9, 4.8 Hz, 2H), 7.27 (dd, *J* = 8.3, 4.7 Hz, 1H), 6.97 (t, *J* = 8.7 Hz, 2H), 6.88 (s, 2H), 3.90 (s, 6H), 3.89 (s, 3H). ^13^C NMR (75 MHz, CDCl_3_ + DMSO): *δ* 172.84, 157.86 (d, *J*_CF_ = 239.25 Hz), 152.91, 147.86, 142.78, 137.44, 134.94, 132.93 (d, *J*_CF_ = 4.2 Hz), 128.73, 122.29, 120.00 (d, *J*_CF_ = 7.6 Hz), 114.88 (d, *J*_CF_ = 22.6 Hz), 105.80, 60.37, 55.64. MS (ESI): *m*/*z* 398 [M + H]^+^. HRMS (ESI) calcd for C_21_H_21_FN_3_O_4_ [M + H]^+^, 398.1516; found, 398.1510.

##### 4.1.3.15. 1-(4-Chlorophenyl)-3-(2-(3,4,5-trimethoxyphenyl)pyridin-3-yl)urea (6o)

Light brown solid, yield 86%, Mp: 218–220 °C; ^1^H NMR (300 MHz, CDCl_3_ + DMSO): *δ* 9.06 (s, 1H), 8.54 (d, *J* = 8.3 Hz, 1H), 8.33 (d, *J* = 4.6 Hz, 1H), 7.74 (s, 1H), 7.47 (s, 1H), 7.40 (d, *J* = 8.8 Hz, 2H), 7.27 (dd, *J* = 7.8, 4.1 Hz, 1H), 7.22 (d, *J* = 8.8 Hz, 2H), 6.88 (s, 2H), 3.90 (d, *J* = 2.3 Hz, 6H), 3.89 (s, 3H). MS (ESI): *m*/*z* 414 [M + H]^+^. HRMS (ESI) calcd for C_21_H_21_ClN_3_O_4_ [M + H]^+^, 414.1221; found, 414.1215.

##### 4.1.3.16. 1-(4-Nitrophenyl)-3-(2-(3,4,5-trimethoxyphenyl)pyridin-3-yl)urea (6p)

Yellow solid, yield 85%, Mp: 261–263 °C; ^1^H NMR (300 MHz, CDCl_3_ + DMSO): *δ* 9.66 (s, 1H), 8.55 (d, *J* = 8.3 Hz, 1H), 8.40–8.34 (m, 1H), 8.15 (d, *J* = 9.2 Hz, 2H), 7.94 (s, 1H), 7.62 (t, *J* = 8.3 Hz, 2H), 7.57 (d, *J* = 3.7 Hz, 1H), 7.31 (dd, *J* = 8.3, 4.7 Hz, 1H), 6.88 (s, 2H), 3.90 (s, 6H), 3.89 (s, 3H). ^13^C NMR (75 MHz, CDCl_3_ + DMSO): *δ* 152.85, 152.06, 148.19, 145.58, 143.39, 141.26, 137.50, 132.72, 132.12, 128.87, 124.50, 122.18, 117.05, 105.79, 60.23, 55.60. MS (ESI): *m*/*z* 424 [M + H]^+^. HRMS (ESI) calcd for C_21_H_21_N_4_O_6_ [M + H]^+^, 425.1461; found, 425.1455.

##### 4.1.3.17. 1-(2-(4-Fluorophenyl)pyridin-3-yl)-3-(4-methoxyphenyl)urea (6q)

White solid, yield 84%, Mp: 191–193 °C; ^1^H NMR (300 MHz, CDCl_3_ + DMSO): *δ* 8.52 (s, 1H), 8.48 (d, *J* = 4.9 Hz, 1H), 8.33 (s, 1H), 7.69–7.61 (m, 2H), 7.58 (s, 1H), 7.41 (s, 1H), 7.31 (d, *J* = 8.8 Hz, 2H), 7.19 (d, *J* = 8.6 Hz, 2H), 6.82 (d, *J* = 8.8 Hz, 2H), 3.78 (s, 3H). ^13^C NMR (75 MHz, CDCl_3_ + DMSO): *δ* 162.24 (d, *J*_CF_ = 245.25 Hz), 154.88, 153.05, 147.57, 143.04, 133.52 (d, *J*_CF_ = 3.8 Hz), 131.87, 130.79 (d, *J*_CF_ = 8.1 Hz), 129.26, 122.40, 120.49, 115.2 (d, *J*_CF_ = 21.4 Hz), 113.68, 55.03. MS (ESI): *m*/*z* 338 [M + H]^+^. HRMS (ESI) calcd for C_19_H_17_FN_3_O_2_ [M + H]^+^, 338.1305; found, 338.1299.

##### 4.1.3.18. 1-(4-Fluorophenyl)-3-(2-(4-fluorophenyl)pyridin-3-yl)urea (6r)

White solid, yield 88%, Mp: 202–204 °C; ^1^H NMR (300 MHz, CDCl_3_ + DMSO): *δ* 8.75 (s, 1H), 8.48 (d, *J* = 8.0 Hz, 1H), 8.35 (d, *J* = 1.7 Hz, 1H), 7.67 (d, *J* = 9.0 Hz, 3H), 7.43–7.35 (m, 2H), 7.31–7.17 (m, 3H), 7.03–6.92 (m, 2H). ^13^C NMR (75 MHz, CDCl_3_ + DMSO): *δ* 162.22 (d, *J*_CF_ = 246 Hz), 157.8 (d, *J*_CF_ = 240 Hz), 152.78, 147.47, 143.25, 134.84, 133.93, 132.71, 130.74 (d, *J*_CF_ = 7.5 Hz), 129.19, 122.28, 119.92 (d, *J*_CF_ = 7.5 Hz), 115.13 (d, *J*_CF_ = 21.75 Hz), 114.67. MS (ESI): *m*/*z* 326 [M + H]^+^. HRMS (ESI) calcd for C_18_H_14_F_2_N_3_O [M + H]^+^, 326.1105; found, 326.1099.

##### 4.1.3.19. 1-(4-Chlorophenyl)-3-(2-(4-fluorophenyl)pyridin-3-yl)urea (6s)

White solid, yield 87%, Mp: 216–2018 °C; ^1^H NMR (300 MHz, CDCl_3_ + DMSO): *δ* 8.83 (s, 1H), 8.46 (d, *J* = 8.3 Hz, 1H), 8.35 (d, *J* = 4.0 Hz, 1H), 7.72 (d, *J* = 6.7 Hz, 1H), 7.67 (dd, *J* = 8.5, 5.5 Hz, 2H), 7.40 (d, *J* = 8.8 Hz, 2H), 7.24 (qd, *J* = 8.4, 5.3 Hz, 5H). ^13^C NMR (75 MHz, CDCl_3_ + DMSO): *δ* 162.14 (d, *J*_CF_ = 246 Hz), 152.45, 147.62, 143.29, 137.75, 133.87, 132.56, 130.74 (d, *J*_CF_ = 8.2 Hz), 129.36, 128.13, 126.19, 122.21, 119.33, 115.11 (d, *J*_CF_ = 21 Hz). MS (ESI): *m*/*z* 342 [M + H]^+^. HRMS (ESI) calcd for C_18_H_14_ClFN_3_O [M + H]^+^, 342.0809; found, 342.0803.

##### 4.1.3.20. 1-(2-(4-Fluorophenyl)pyridin-3-yl)-3-(4-nitrophenyl)urea (6t)

Yellow solid, yield 85%, Mp: 203–205 °C; ^1^H NMR (300 MHz, CDCl_3_ + DMSO): *δ* 9.43 (s, 1H), 8.49–8.35 (m, 2H), 8.15 (dd, *J* = 9.2, 2.8 Hz, 2H), 7.95 (s, 1H), 7.68 (dd, *J* = 7.2, 4.3 Hz, 2H), 7.62 (dd, *J* = 9.3, 2.5 Hz, 2H), 7.35–7.18 (m, 3H). ^13^C NMR (75 MHz, CDCl_3_ + DMSO): *δ* 162.27 (d, *J*_CF_ = 246.3 Hz), 152.04, 147.96, 145.55, 143.91, 141.29, 133.74, 132.04, 130.80 (d, *J*_CF_ = 8.1 Hz), 129.69, 124.51, 122.31, 117.39, 117.10, 115.22 (d, *J*_CF_ = 21.3 Hz). MS (ESI): *m*/*z* 353 [M + H]^+^. HRMS (ESI) calcd for C_18_H_14_FN_4_O_3_ [M + H]^+^, 353.1050; found, 353.1044.

##### 4.1.3.21. 1-(4-Methoxyphenyl)-3-(2-(4-(trifluoromethyl)phenyl)pyridin-3-yl)urea (6u)

White solid, yield 90%, Mp: 211–213 °C; ^1^H NMR (300 MHz, CDCl_3_ + DMSO): *δ* 8.59 (s, 1H), 8.50 (d, *J* = 8.1 Hz, 1H), 8.36 (d, *J* = 4.4 Hz, 1H), 7.89–7.78 (m, 4H), 7.65–7.57 (m, 1H), 7.32 (d, *J* = 8.8 Hz, 3H), 6.82 (d, *J* = 8.9 Hz, 2H), 3.77 (s, 3H). ^13^C NMR (75 MHz, CDCl_3_ + DMSO): *δ* 154.73, 152.79, 146.83, 143.27, 141.71, 133.14, 131.79, 129.48, 129.33, 125.02 (q, *J*_CF_3__ = 3.3 Hz), 122.76, 121.85, 120.18, 113.55, 54.90. MS (ESI): *m*/*z* 388 [M + H]^+^. HRMS (ESI) calcd for C_20_H_17_F_3_N_3_O_2_ [M + H]^+^, 388.1273; found, 388.1267.

##### 4.1.3.22. 1-(4-Fluorophenyl)-3-(2-(4-(trifluoromethyl)phenyl)pyridin-3-yl)urea (6v)

White solid, yield 86%, Mp: 244–246 °C; ^1^H NMR (300 MHz, CDCl_3_ + DMSO): *δ* 8.74 (s, 1H), 8.46 (d, *J* = 12.9 Hz, 1H), 8.38 (s, 1H), 7.83 (d, *J* = 10.9 Hz, 5H), 7.53 (s, 1H), 7.39 (s, 2H), 7.32 (d, *J* = 3.4 Hz, 1H), 7.00 (d, *J* = 8.5 Hz, 2H). ^13^C NMR (75 MHz, DMSO): *δ* 159.36, 152.70, 146.96, 143.54, 141.66, 134.82, 132.91, 129.67, 129.35, 125.03 (d, *J*_CF_3__ = 3.6 Hz), 122.80, 119.88 (d, *J*_CF_ = 7.7 Hz), 114.95, 114.66. MS (ESI): *m*/*z* 376 [M + H]^+^. HRMS (ESI) calcd for C_19_H_14_F_4_N_3_O [M + H]^+^, 376.1073; found, 376.1067.

##### 4.1.3.23. 1-(4-Chlorophenyl)-3-(2-(4-(trifluoromethyl)phenyl)pyridin-3-yl)urea (6w)

White solid, yield 84%, Mp: 216–218 °C; ^1^H NMR (300 MHz, CDCl_3_ + DMSO): *δ* 8.86 (s, 1H), 8.46 (d, *J* = 8.4 Hz, 1H), 8.38 (d, *J* = 4.5 Hz, 1H), 7.86 (d, *J* = 10.8 Hz, 3H), 7.79 (d, *J* = 8.4 Hz, 2H), 7.40 (d, *J* = 8.8 Hz, 2H), 7.33 (dd, *J* = 8.4, 4.6 Hz, 1H), 7.22 (d, *J* = 8.8 Hz, 2H). MS (ESI): *m*/*z* 392 [M + H]^+^. HRMS (ESI) calcd for C_19_H_14_ClF_3_N_3_O [M + H]^+^, 392.0777; found, 392.0772.

##### 4.1.3.24. 1-(4-Nitrophenyl)-3-(2-(4-(trifluoromethyl)phenyl)pyridin-3-yl)urea (6x)

Yellow solid, yield 91%, Mp: 234–236 °C; ^1^H NMR (300 MHz, CDCl_3_ + DMSO): *δ* 9.39 (s, 1H), 8.48–8.40 (m, 2H), 8.15 (d, *J* = 9.2 Hz, 2H), 8.04 (s, 1H), 7.86–7.80 (m, 4H), 7.61 (d, *J* = 9.4 Hz, 2H), 7.36 (dd, *J* = 8.3, 4.7 Hz, 1H). ^13^C NMR (75 MHz, CDCl_3_ + DMSO): *δ* 151.98, 147.76, 145.63, 144.28, 141.61, 141.24, 132.30, 130.37, 129.47, 125.09 (d, *J*_CF_3__ = 3.2 Hz), 124.53, 122.89, 117.21. MS (ESI): *m*/*z* 403 [M + H]^+^. HRMS (ESI) calcd for C_19_H_14_F_3_N_4_O_4_ [M + H]^+^, 403.1018; found, 403.1012.

### 4.2. Biology

#### 4.2.1. Cell culture

Prostate (DU145) and cervical (HeLa) cancer cells were generously provided by Prof. Roger Daly (Peter MacCallum Cancer Centre, Melbourne). Human breast (MDA-MB-231) and human lung (A549) cells were purchased from American Type Culture Collection (MD, USA). The cell lines were maintained in RPMI medium supplemented with 10% fetal bovine serum, 100 U per mL penicillin, and 100 µg per mL streptomycin at 37 °C in a humidified atmosphere with 5% CO_2_. Stock solutions of the gold complexes (10.0 M) were prepared in DMSO, and working concentrations were prepared with a final DMSO concentration of less than 1%.

#### 4.2.2. MTT assay

In this assay, prostate (DU-145), lung (A549), breast (MDA-MB-231), and cervical (HeLa) cancer cells were seeded in 96-well plates according to their doubling times and were grown overnight. The cells were exposed to different concentrations of target molecules 6a–x (100, 10, 1, 0.1, and 0.01 µM) for 72 h. Then, the medium containing compounds was removed and replaced with 100 µL of MTT solution (5 mg mL^−1^), and the cells were further incubated for 4 h in the dark at 37 °C. The unreacted MTT solution was removed, and 100 µL DMSO was added to each well to solubilize the produced formazan crystals. The absorbance of the purple formazan solution was recorded using a plate reader (SpectraMax) at 570 nm, and the IC_50_ values for each compound were calculated. All the experiments were repeated three times, and the standard deviations are reported in Table 1.^23^

#### 4.2.3. Cell cycle

A549 cells in 6-well plates were seeded at a density of 1 × 10^6^ per well and were grown overnight in an incubator. The cells were then incubated with 1 and 2 µM concentrations of compound 6q, and after 48 h, collected using 0.25% trypsin–EDTA. The obtained cell pellets were washed and resuspended in PBS. The cells were fixed by pipetting the resuspended cell suspension into 9 mL of 70% ethanol. After 30 minutes of fixation at 4 °C, the ethanol was removed by centrifugation, and the cells were washed with PBS. After centrifugation, the cells were incubated with a propidium iodide staining solution for 15 minutes in the dark at room temperature. 10 000 cells from each sample were analyzed for DNA content (propidium iodide fluorescence) using a BD Accuri C6 flow cytometer. The final flow cytometry data was analyzed by using FlowJo software.^24^

#### 4.2.4. Inhibition of tubulin polymerization

For the morphological analysis of the nucleus and tubulin network, the protocol was followed as mentioned. A549 cells were seeded on a glass coverslip and incubated for 48 h in the presence or absence of test compounds 6q and nocodazole at concentrations of 1 and 2 µM. Cells grown on coverslips were fixed in 4% formaldehyde in phosphate-buffered saline (PBS), pH 7.4, for 10 min at room temperature. Cells were permeabilized for 6 minutes in PBS containing 0.5% Triton X-100 (Sigma) and 0.05% Tween 20 (Sigma). The permeabilized cells were blocked with 2% BSA (Sigma) in PBS for 1 h. Later, the cells were incubated with the primary antibody for tubulin from Sigma at a 1 : 200 dilution in blocking solution for 4 h at room temperature. Subsequently, the antibodies were removed, and the cells were washed three times with PBS. Cells were then incubated with the FITC-labeled anti-mouse secondary antibody (1 : 500) for 1 h at room temperature. Cells were washed three times with PBS and then mounted in a medium containing DAPI. Images were captured using a confocal microscope.^25^

#### 4.2.5. Molecular docking studies

The molecular docking studies were performed at the binding site of Colchicine.^26^ The coordinates of the crystal structure were obtained from the RCSB Protein Data Bank, and suitable corrections were made using the Protein Preparation Wizard from the Schrödinger package. Regarding the ligands, molecules were constructed using ChemBio3D Ultra 16.0, and their geometries were optimized using molecular mechanics. Finally, docking studies were performed according to the standard protocol implemented in Maestro software, version 2023-1, for the most active molecules.^30^ The ligand–protein complex was analyzed for interactions, and the 3D pose of the most active compounds was imaged using Schrödinger software.

#### 4.2.6. Colony-forming assay

A549 cells in the exponential growth phase were seeded into 6-well plates at a density of 4000 cells per well. After 24 h incubation, the culture medium was replaced with medium containing increasing concentrations (1, 2, and 3 µM) of compound 6q and 1% DMSO (control). The cells were incubated for 7 days, and the drug-containing medium was replenished after 3 days. Each treatment was performed in triplicate. After incubation, the cells were washed twice with PBS, fixed with 4% paraformaldehyde for 20 minutes, and stained with crystal violet for an additional 15 minutes.^25^

#### 4.2.7. Wound healing assay (migration assay)

Confluent A549 monolayers in 30 mm Petri dishes were wounded with 200 µL pipette tips, giving rise to 1 mm wide lanes per well. The cell debris was removed by washing with PBS, and cells were supplied with 2 mL of complete medium (controls) or complete medium containing different concentrations of compound 6q (1, 2, and 3 µM). The wounds were observed by phase contrast microscopy immediately and after 30 h of incubation.^25^

#### 4.2.8. Identification of apoptotic cells (Hoechst staining)

Changes in the nuclear morphology of A549 cells were determined using Hoechst 33342. In this assay, A549 cells were grown on cover slips in a 6-well plate at a density of 1 × 10^6^ cells per well and were incubated with different concentrations (1, 2, and 3 µM) of compound 6q for 48 h. The cells were washed with PBS, and 4% paraformaldehyde solution was added. The cells were incubated with 2 µg mL^−1^ Hoechst 33242 for 20 minutes, followed by three washes with PBS to remove excess dye. The morphological changes in the nuclei were observed using a ZOE™ Fluorescent Cell Imager (BIO-RAD).^27^

#### 4.2.9. Effect on mitochondrial membrane potential

The A549 cells were plated at 1 × 10^5^ cells per mL in 24-well culture plates and allowed to adhere overnight. Cells were treated with 1, 2, and 3 µM concentrations of the compound 6q for 48 h. After washing twice with PBS, cells were incubated with rhodamine 123 (Sigma Aldrich) (5 µg mL^−1^) for 30 min in the dark at 37 °C, and cells were washed twice with PBS to remove the excess dye. The decrease in intensity of fluorescence because of mitochondrial membrane potential loss was analyzed using a ZOE™ Fluorescent Cell Imager (BIO-RAD).^28^

#### 4.2.10. Quantification of apoptotic cells (annexin-V FITC assay)

A549 cells (1 × 10^6^ per well) were grown in 6 well plate and treated with 1.5 and 3 µM concentrations of compound 6q for 48 h. After incubation, the cells were trypsinized and washed with PBS. The obtained cell pellet was resuspended in 1× annexin binding buffer. 5 µL of annexin V and 1 µL of PI were added to the resuspended cells, and the mixture was incubated at room temperature for 15 minutes. 10 000 cells from each sample were used for analysis, where the obtained flow cytometry data was analyzed by using FlowJo software.^23^

## Conflicts of interest

There are no conflicts of interest to declare.

## Supplementary Material

RA-016-D5RA09372D-s001

## Data Availability

All data supporting the findings of this study are available within the main article and its supplementary information (SI). Supplementary information: NMR, LC-MS and HRMS spectra. See DOI: https://doi.org/10.1039/d5ra09372d.
