# Supplementary material for: Synthesis and anti-proliferative activity of new E7010 tethered urea congeners as potential tubulin inhibitors and apoptosis inducers
Source: RSC Adv. 2026 Jan 19;16(5):4141–56. doi: 10.1039/d5ra09372d (PMC12814937; doi:10.1039/d5ra09372d)

Supplementary Information (SI) for RSC Advances.
This journal is © The Royal Society of Chemistry 2026

Supporting Information

Synthesis and anti-proliferative activity of new E7010 tethered urea congeners as potential tubulin inhibitors and apoptosis inducers

Shaik Taj,[a,b] Ganga Reddy Velma,*[c,d] Srinivasa Reddy Telukutla,[d,e] Satyaveni Malasala,[f] Anjali Sharma,[g] Irfan Khan,[a,c] Mohd Adil Shareef,[a,b] Suresh K. Bhargava,[d] Magdalena Plebanski,[e] Bathini Nagendra Babu,[a,b] Ahmed Kamal* [a,h]

[a]*Academy of Scientific and Innovative Research (AcSIR), CSIR-Human Resource Development Centre (CSIR-HRDC) Campus, Ghaziabad 201 002, Uttar Pradesh, India*

[b]*Fluoro-Agrochemicals, CSIR-Indian Institute of Chemical Technology (IICT), Hyderabad 500 007, India*

[c]*Department of Pharmacology and Toxicology, College of Pharmacy, University of Arizona, Tucson 85721, AZ, USA*

[d]*Centre for Advanced Materials & Industrial Chemistry (CAMIC), School of Science,*

RMIT University, GPO Box 2476, Melbourne 3001, Australia

[e]*School of Health and Biomedical Sciences, RMIT University, Melbourne, 3083, Australia*

[f]*Department of Anatomy & Cell Biology, The Brody School of Medicine, East Carolina University, Greenville, NC, USA*

[g]*Guru Gobind Singh College of Pharmacy, Yamunanagar (135001), Haryana, India.*

[h]*Department of Pharmacy, Birla Institute of Technology and Science-Pilani, Hyderabad 500078, India*

Corresponding authors:

Prof. Ahmed Kamal, ahmedkamal@hyderabad.bits-pilani.ac.in, ahmedkamal915@gmail.com

Dr. Ganga Reddy Velma, E-mail: velmagangareddy47@gmail.com; vgreddy@arizona.edu

Contents | Page No

1. ^{1}H NMR and ^{13}C NMR spectra — S2-S23
2. LC-MS spectra — S24-35
3. HRMS Reports — S36-47

^{1}H and ^{13}C NMR spectra

Compound **6a**

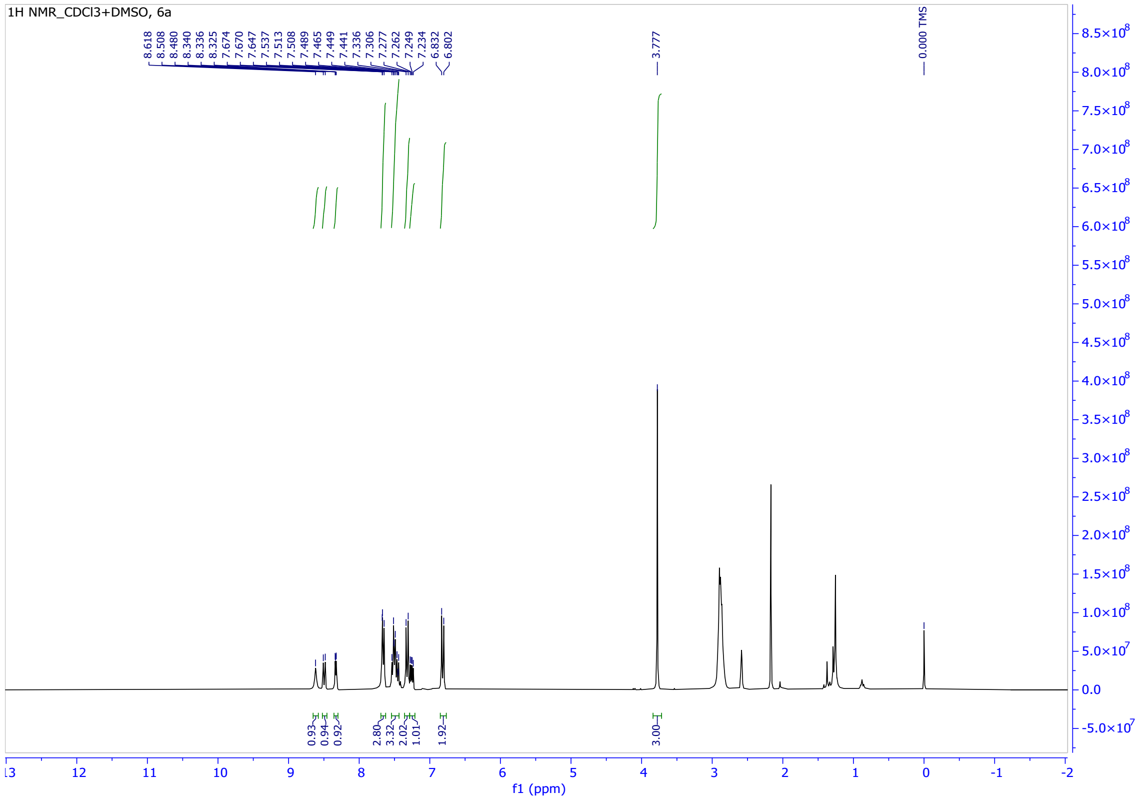

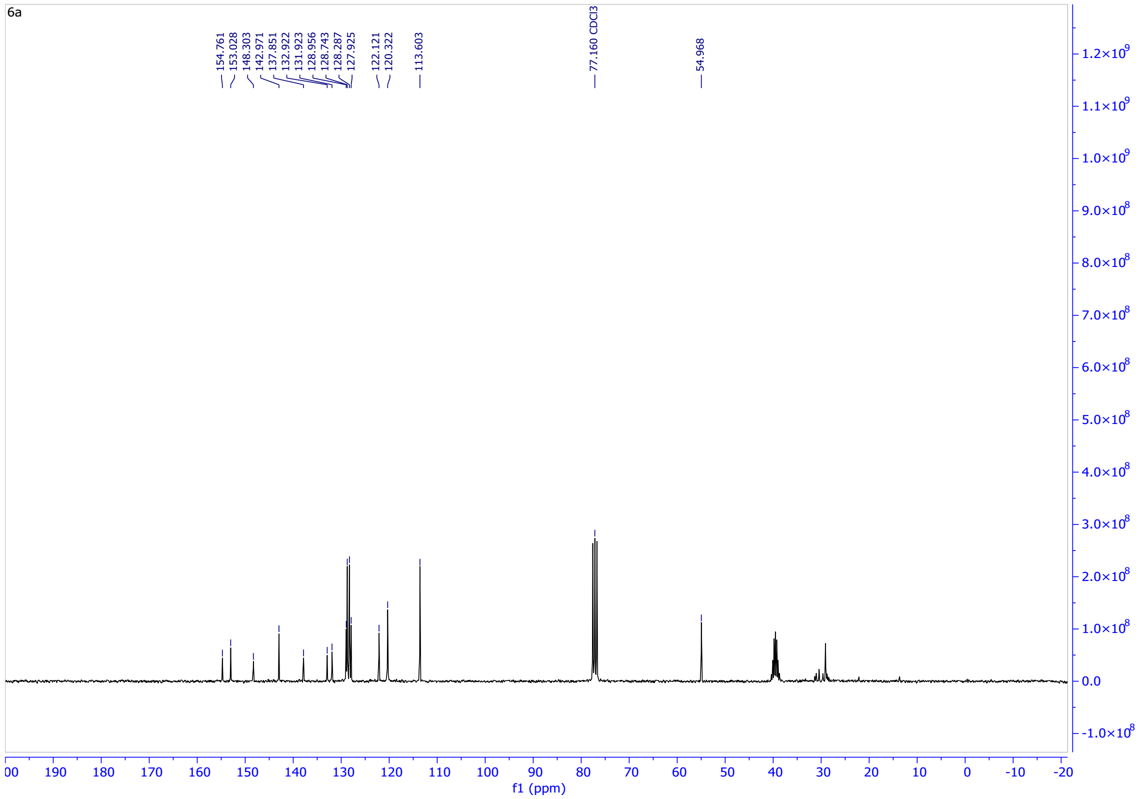

Compound **6b**

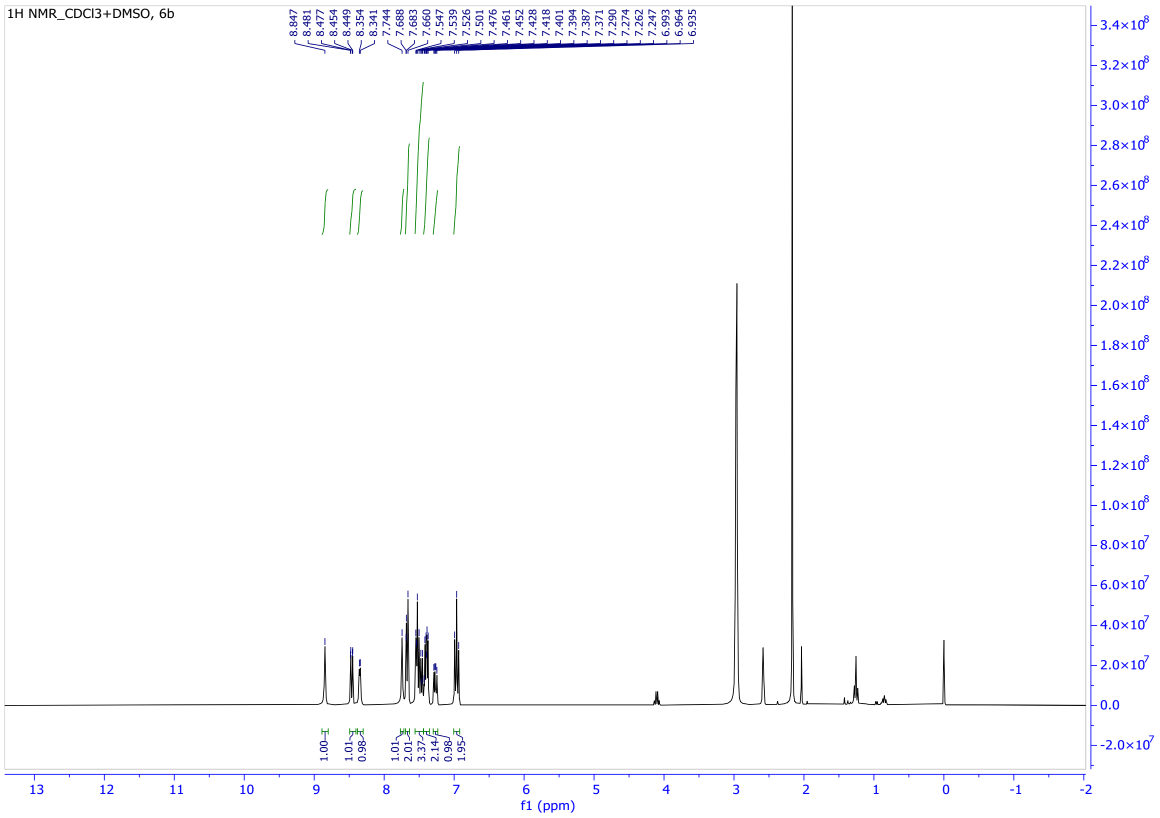

1H NMR_CDCl3+DMSO, 6b
8.847
8.481
8.477
8.454
8.449
8.354
8.341
7.744
7.688
7.683
7.660
7.547
7.539
7.526
7.501
7.476
7.461
7.452
7.428
7.418
7.401
7.394
7.387
7.371
7.290
7.274
7.262
7.247
6.993
6.964
6.935
1.00
1.01
0.98
1.01
2.01
3.37
2.14
0.98
1.95
f1 (ppm)

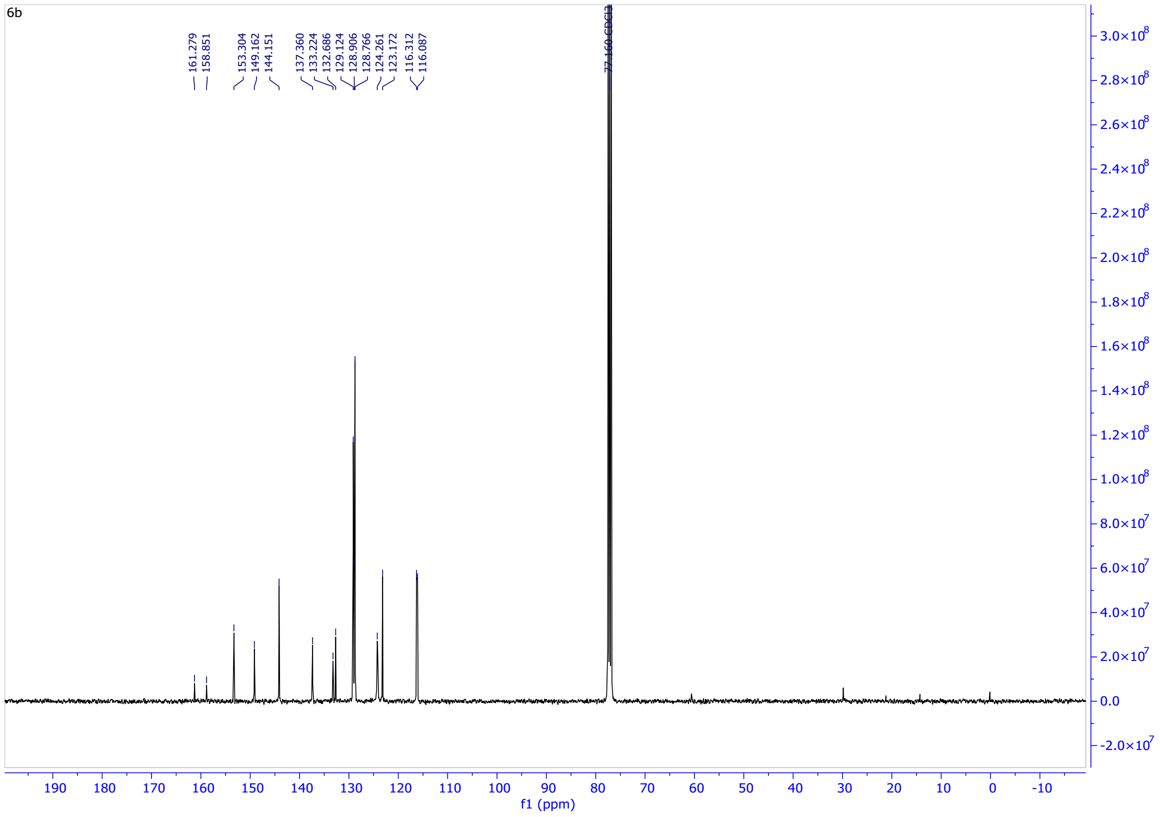

6b
161.279
158.851
153.304
149.162
144.151
137.360
133.224
132.686
129.124
128.906
128.766
124.261
123.172
116.312
116.087
77.160 CDCl3
f1 (ppm)

Compound **6c**

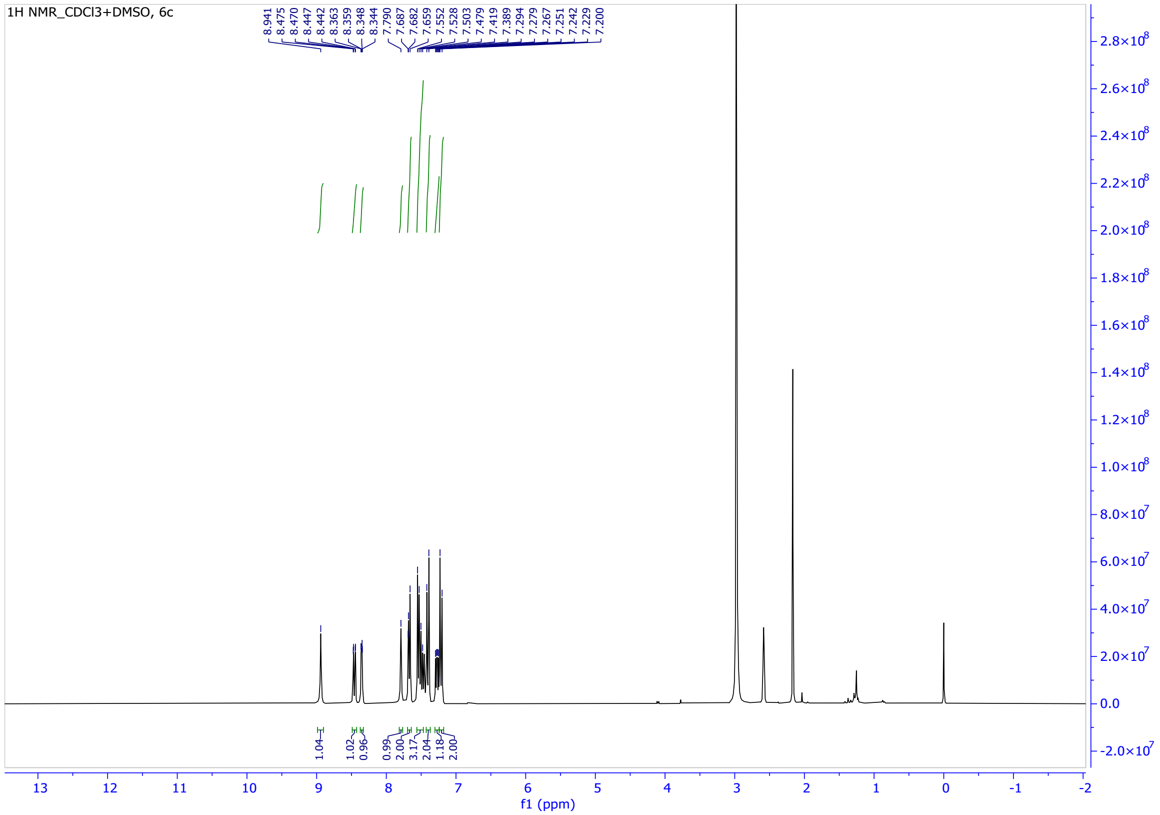
1H NMR_CDCl3+DMSO, 6c
8.941
8.475
8.470
8.447
8.442
8.363
8.359
8.348
8.344
7.790
7.687
7.682
7.659
7.552
7.528
7.503
7.479
7.419
7.389
7.294
7.279
7.267
7.251
7.242
7.229
7.200
1.04
1.02
0.96
0.99
2.00
3.17
2.04
1.18
2.00
f1 (ppm)

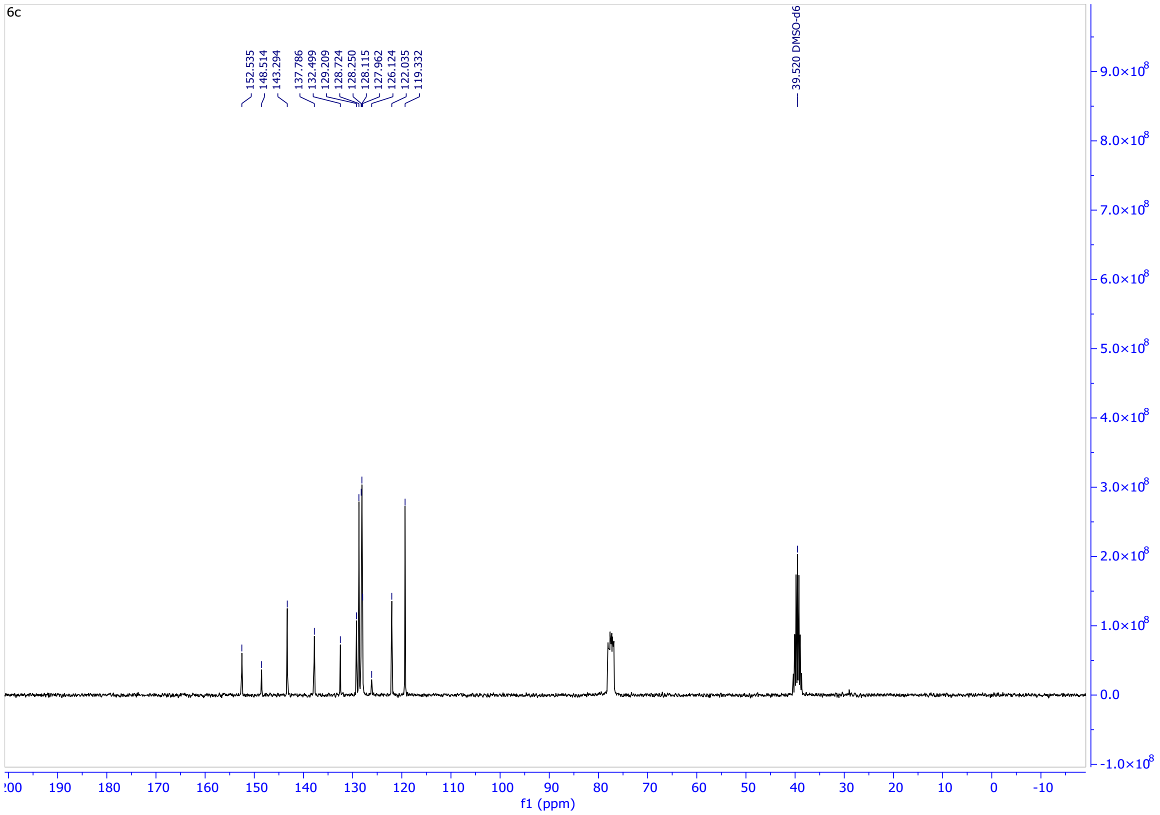
6c
152.535
148.514
143.294
137.786
132.499
129.209
128.724
128.250
128.115
127.962
126.124
122.035
119.332
39.520 DMSO-d6
f1 (ppm)

Compound **6d**

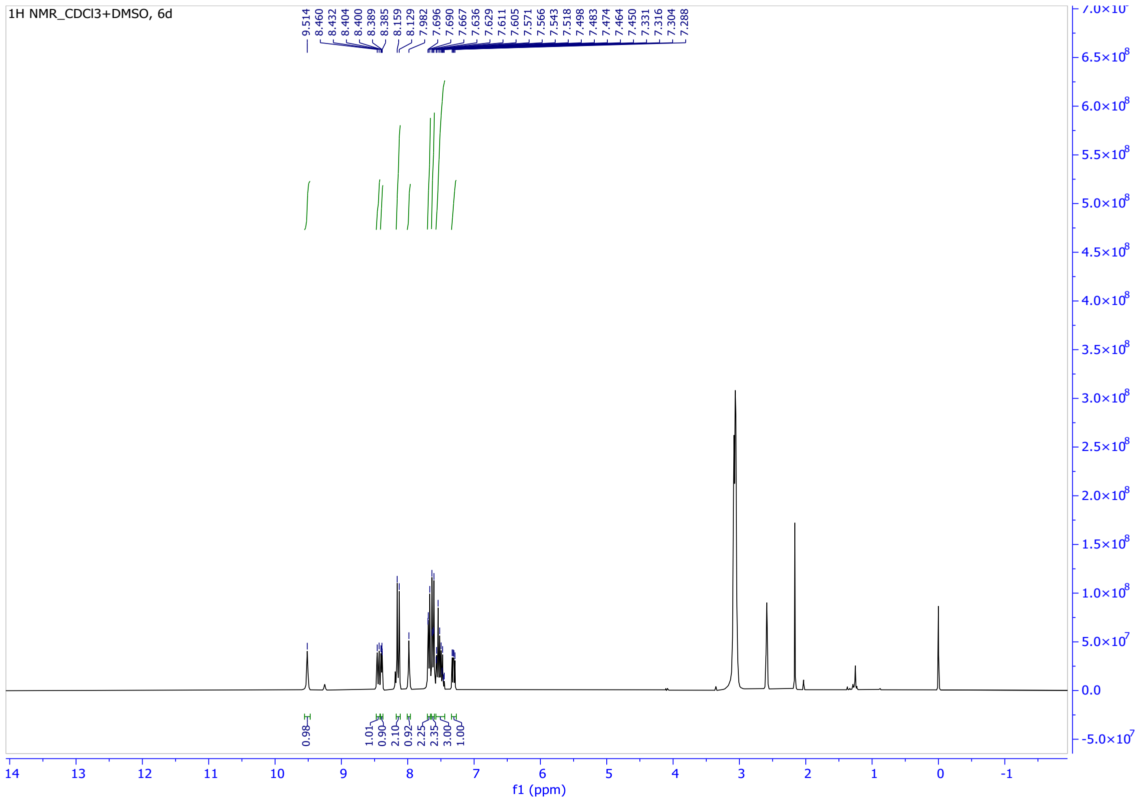

Compound **6e**

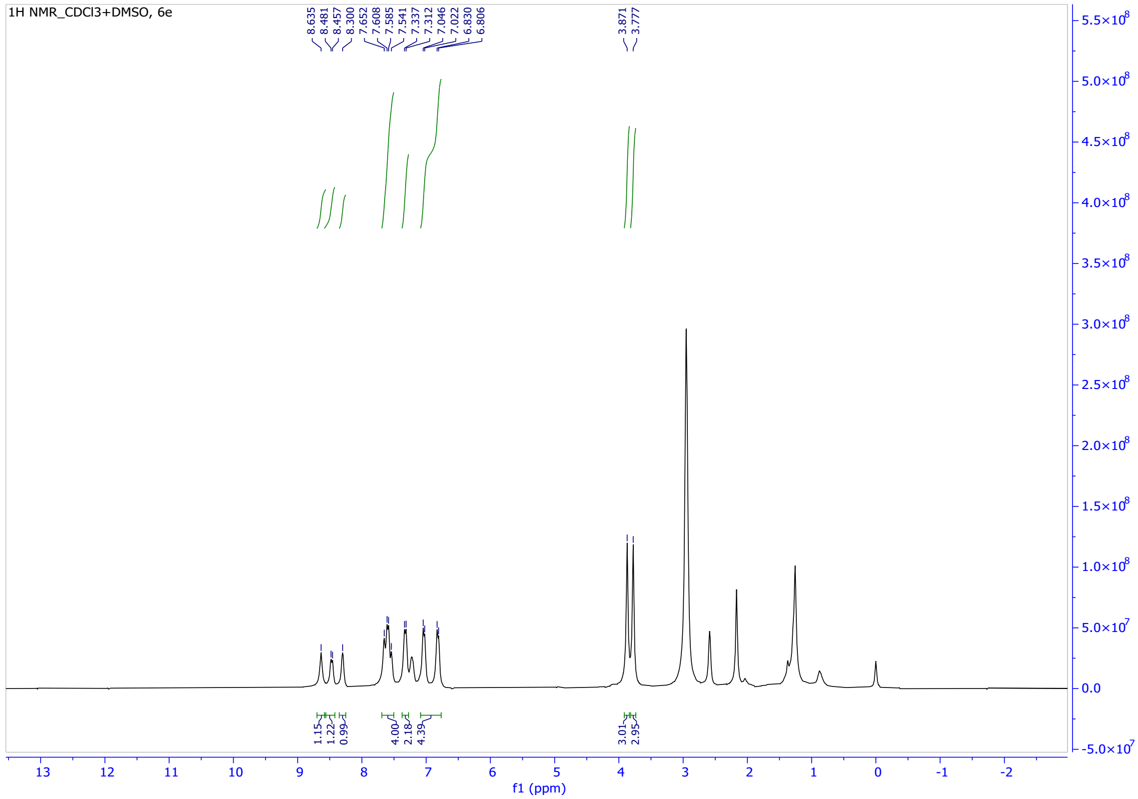

Compound **6f**

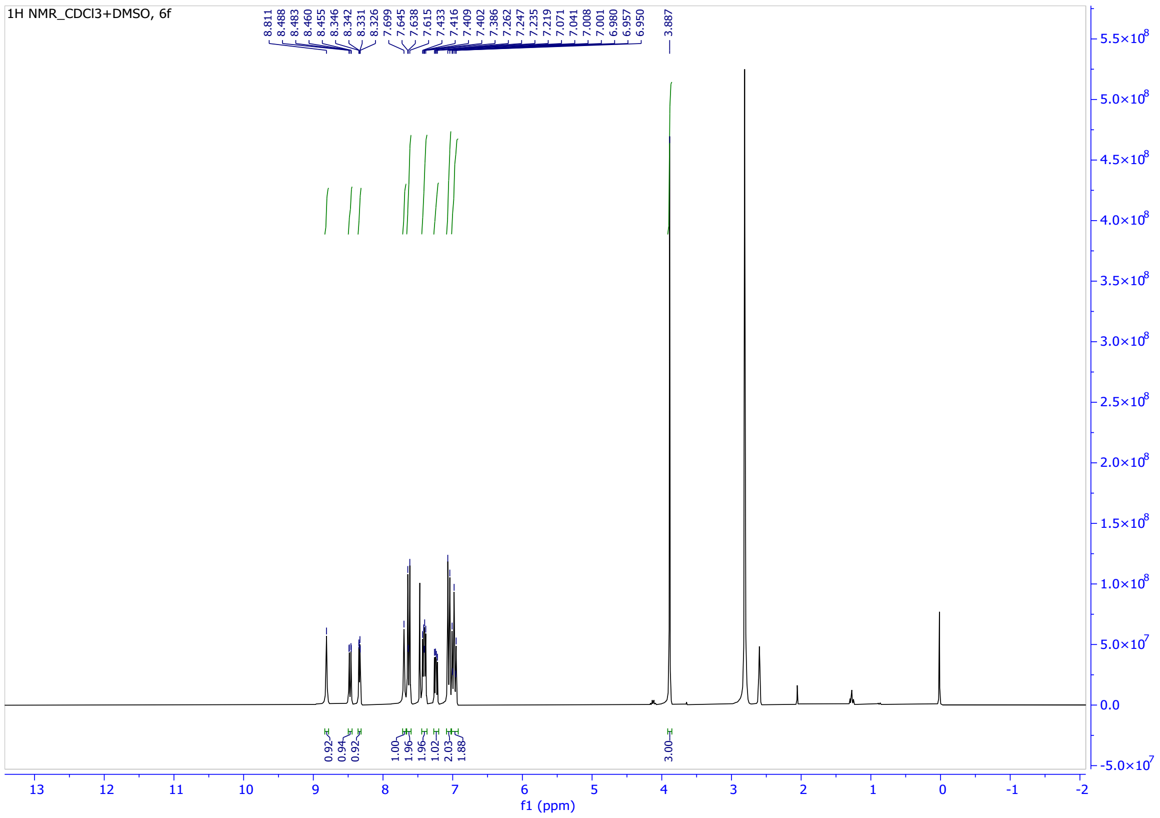
1H NMR_CDCl3+DMSO, 6f
8.811
8.488
8.483
8.460
8.455
8.346
8.342
8.331
8.326
7.699
7.645
7.638
7.615
7.433
7.416
7.409
7.402
7.386
7.262
7.247
7.235
7.219
7.071
7.041
7.008
7.001
6.980
6.957
6.950
3.887
0.92
0.94
0.92
1.00
1.96
1.96
1.02
2.03
1.88
3.00
f1 (ppm)

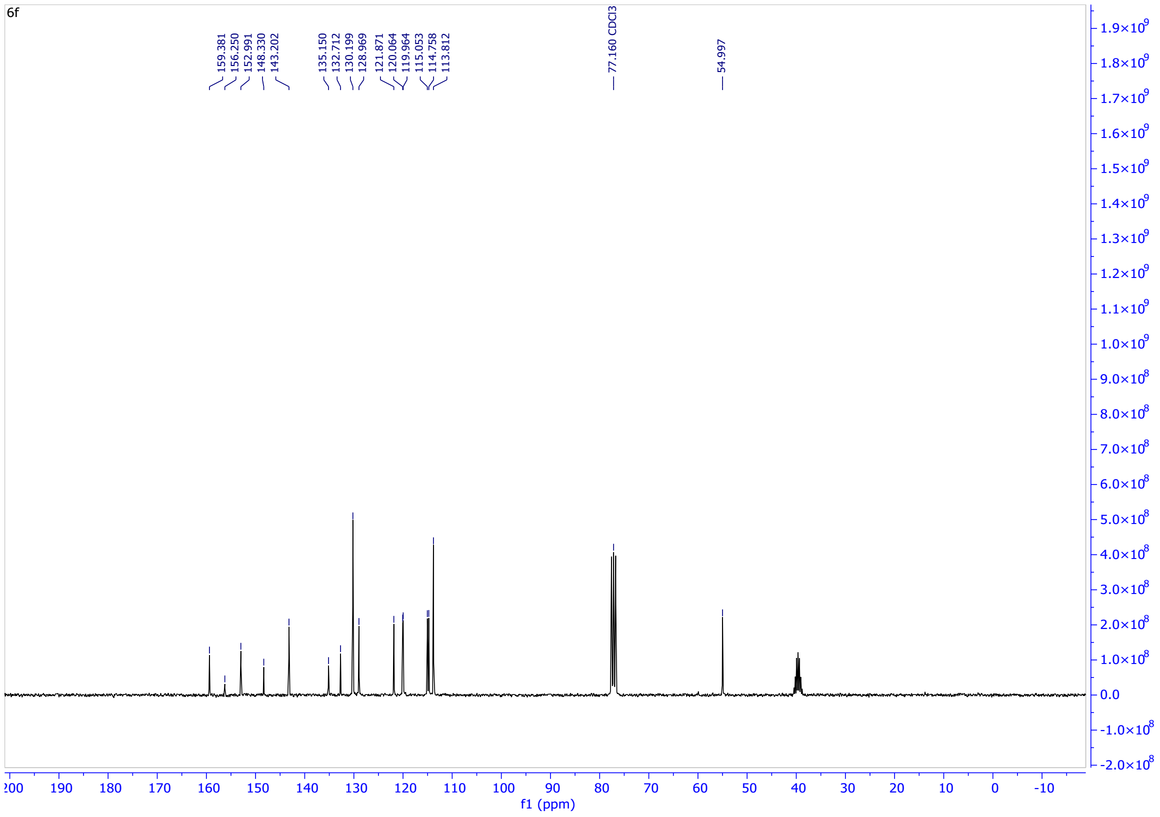
6f
159.381
156.250
152.991
148.330
143.202
135.150
132.712
130.199
128.969
121.871
120.064
119.964
115.053
114.758
113.812
77.160 CDCl3
54.997
f1 (ppm)

Compound **6g**

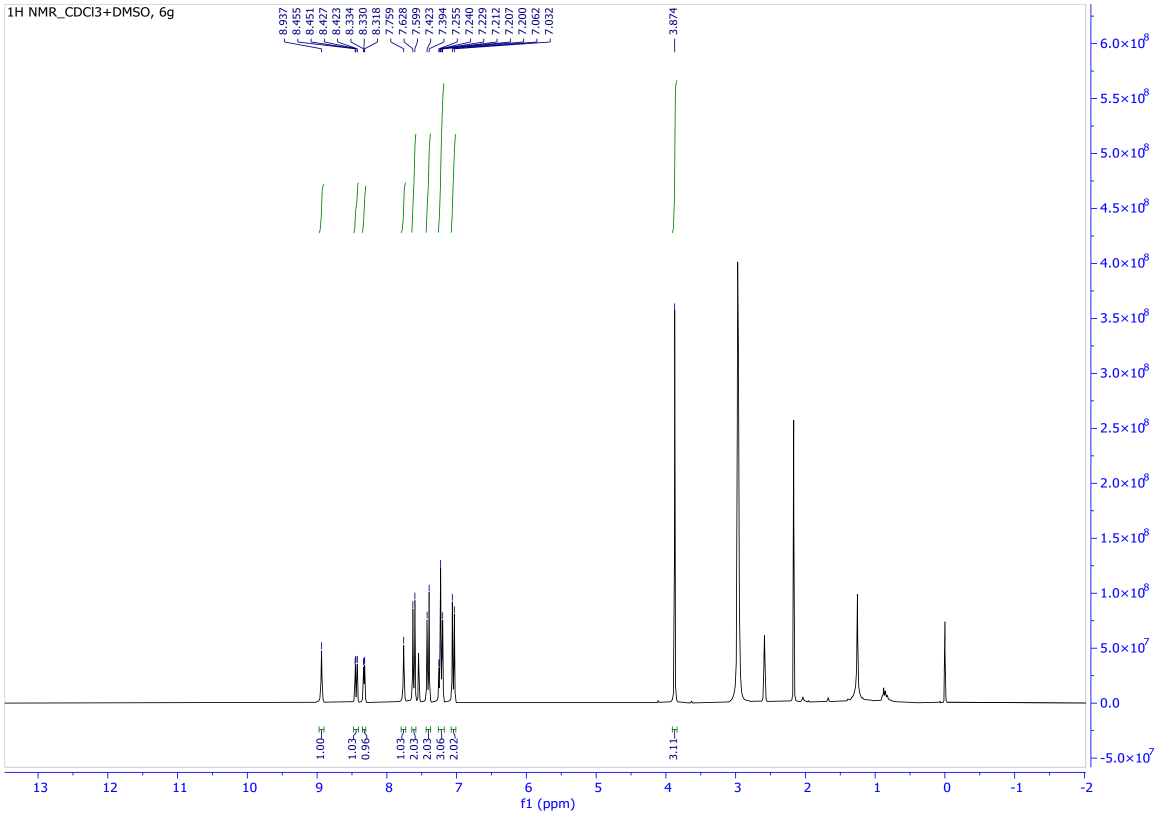

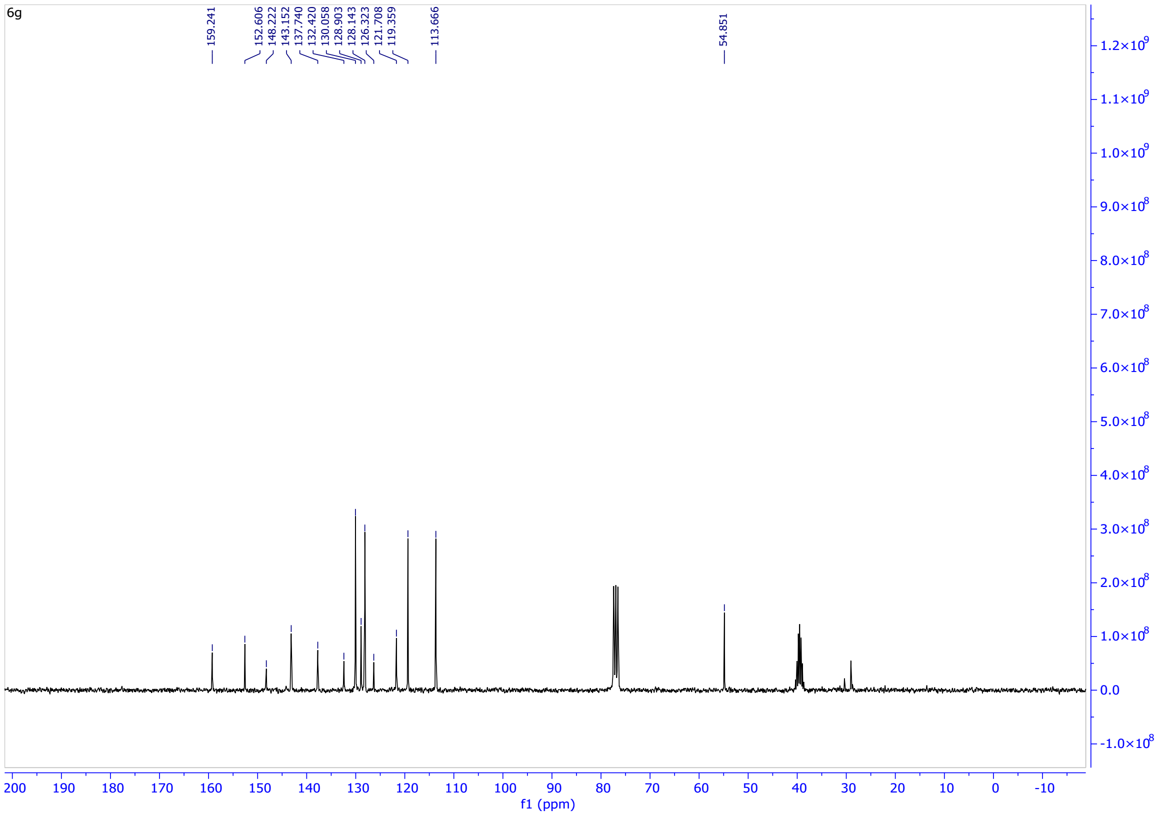

Compound **6h**

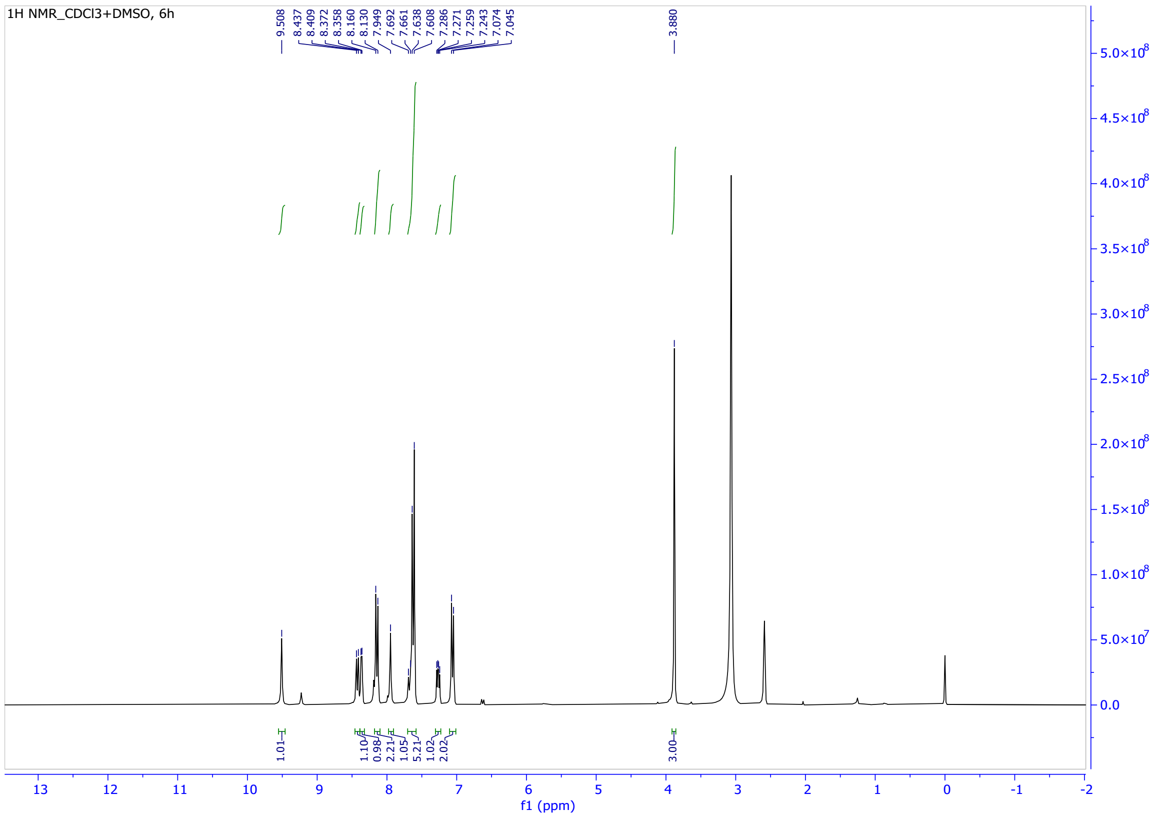

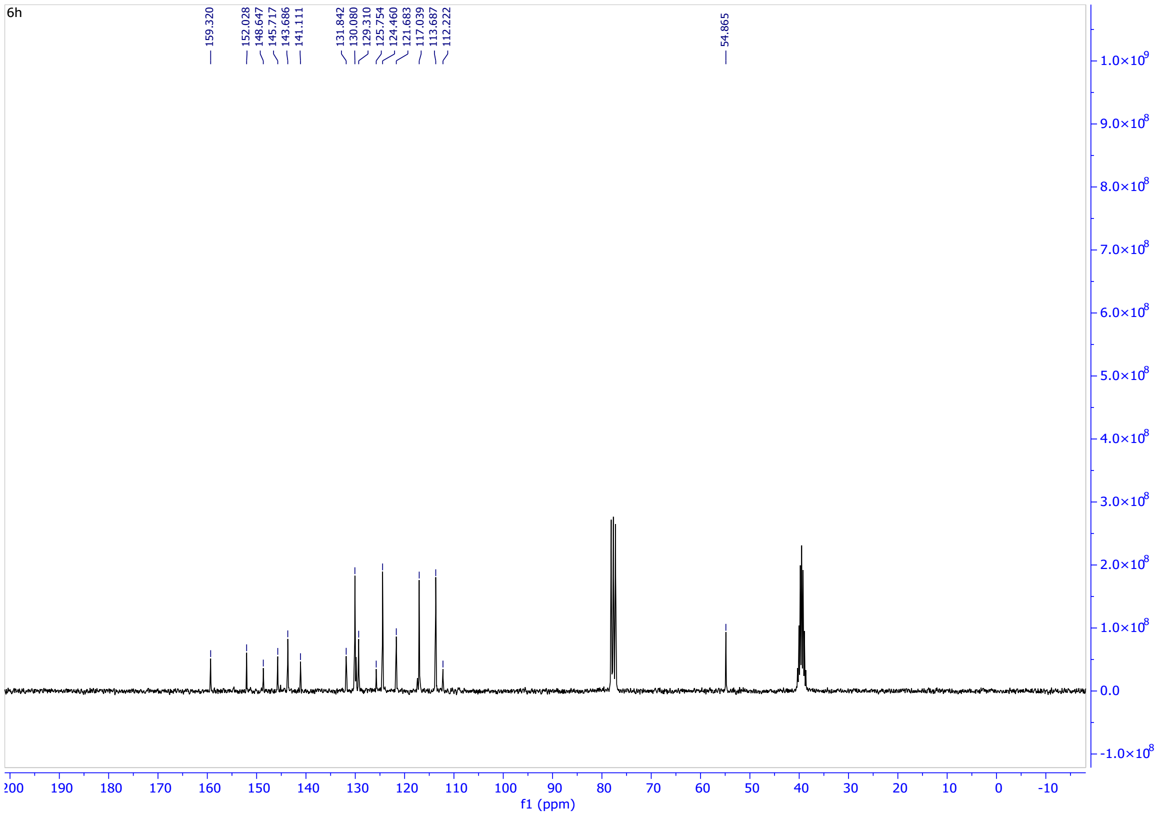

Compound **6i**

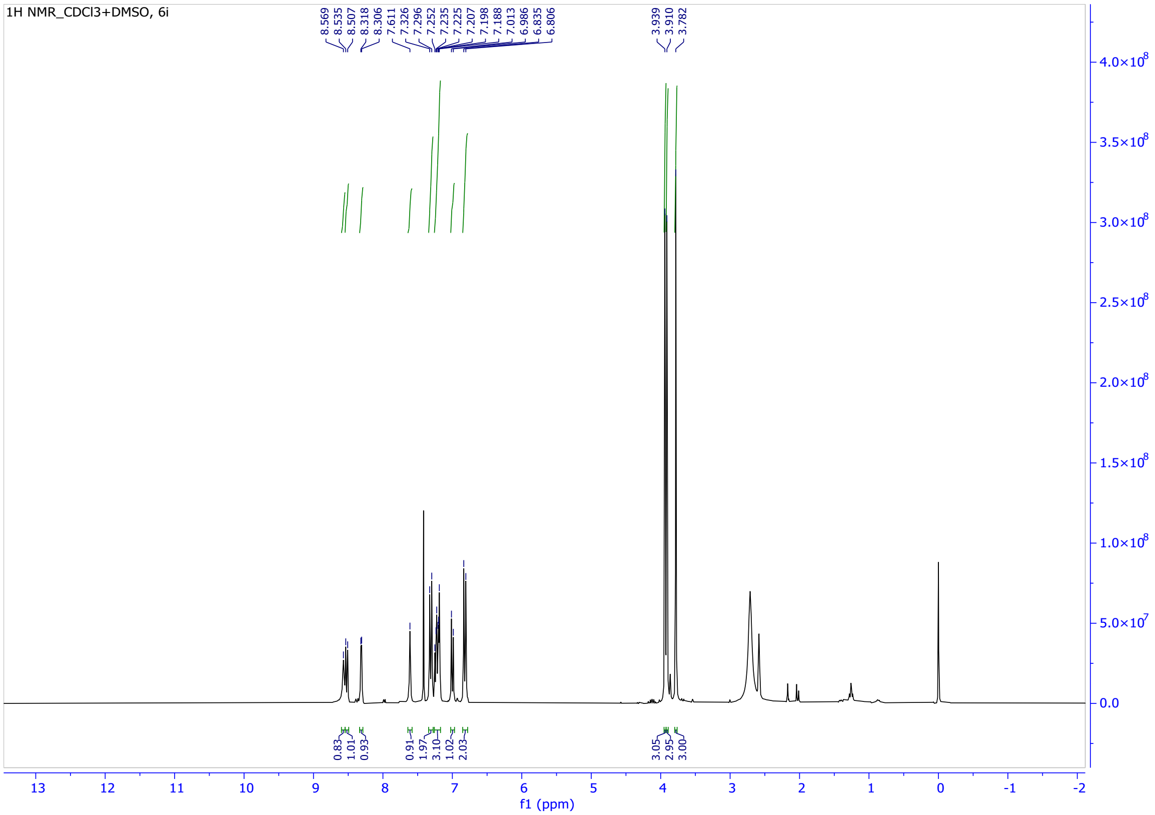

1H NMR_CDCl3+DMSO, 6i
8.569
8.535
8.507
8.318
8.306
7.611
7.326
7.296
7.252
7.235
7.225
7.207
7.198
7.188
7.013
6.986
6.835
6.806
3.939
3.910
3.782
0.83
1.01
0.93
0.91
1.97
3.10
1.02
2.03
3.05
2.95
3.00
f1 (ppm)

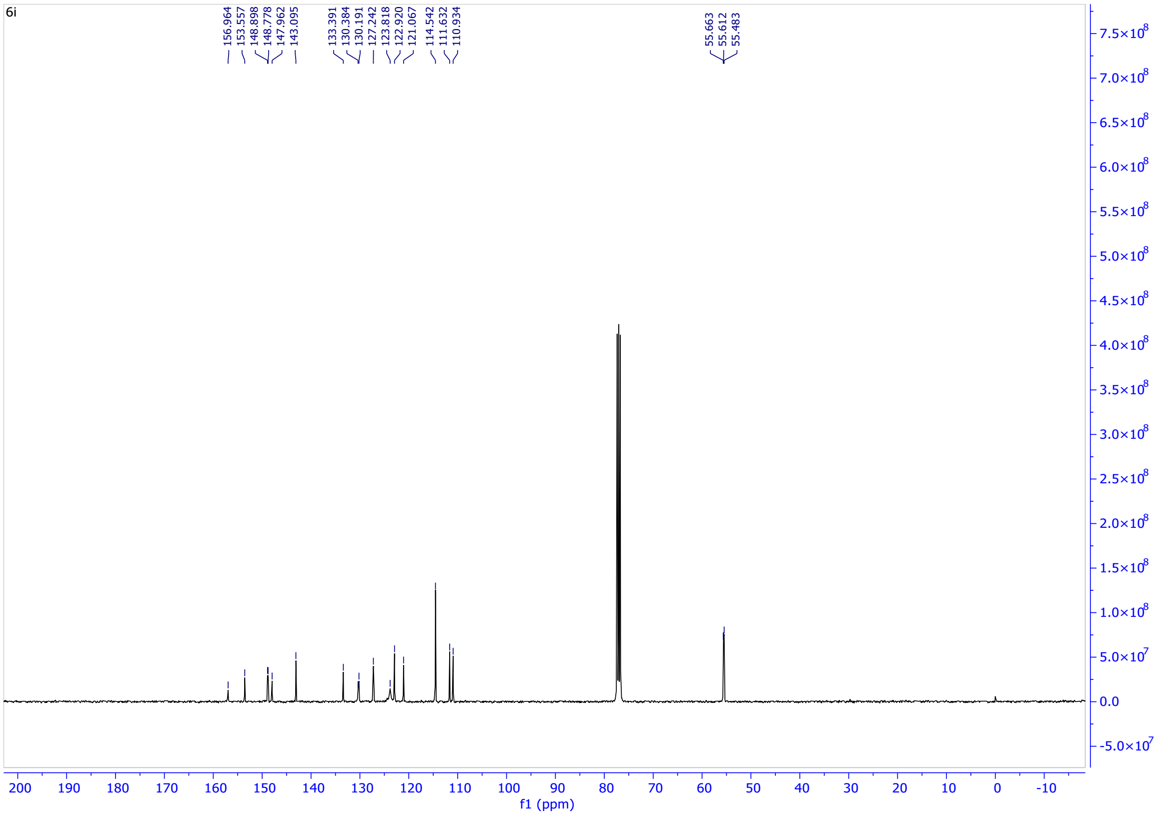

6i
156.964
153.557
148.898
148.778
147.962
143.095
133.391
130.384
130.191
127.242
123.818
122.920
121.067
114.542
111.632
110.934
55.663
55.612
55.483
f1 (ppm)

Compound **6j**

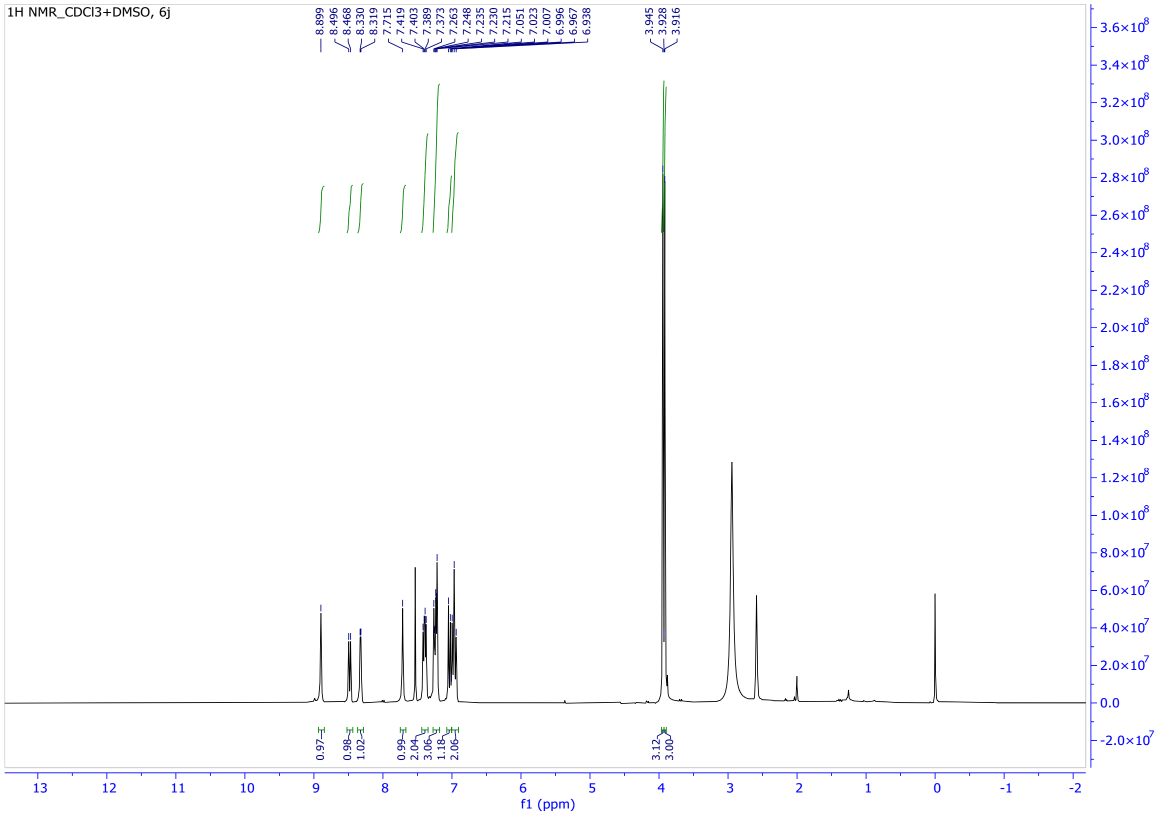
1H NMR_CDCl3+DMSO, 6j
8.899
8.496
8.468
8.330
8.319
7.715
7.419
7.403
7.389
7.373
7.263
7.248
7.235
7.230
7.215
7.051
7.023
7.007
6.996
6.967
6.938
3.945
3.928
3.916
0.97
0.98
1.02
0.99
2.04
3.06
1.18
2.06
3.12
3.00
f1 (ppm)

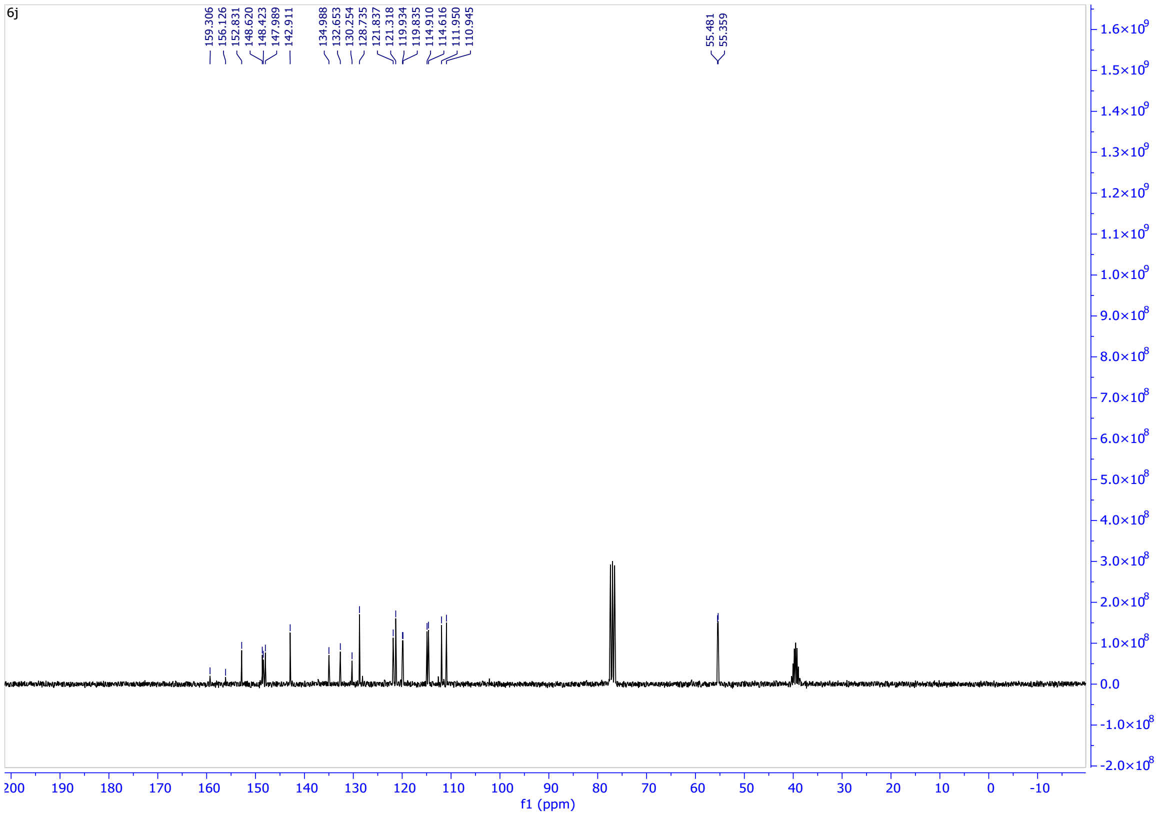
6j
159.306
156.126
152.831
148.620
148.423
147.989
142.911
134.988
132.653
130.254
128.735
121.837
121.318
119.934
119.835
114.910
114.616
111.950
110.945
55.481
55.359
f1 (ppm)

Compound **6k**

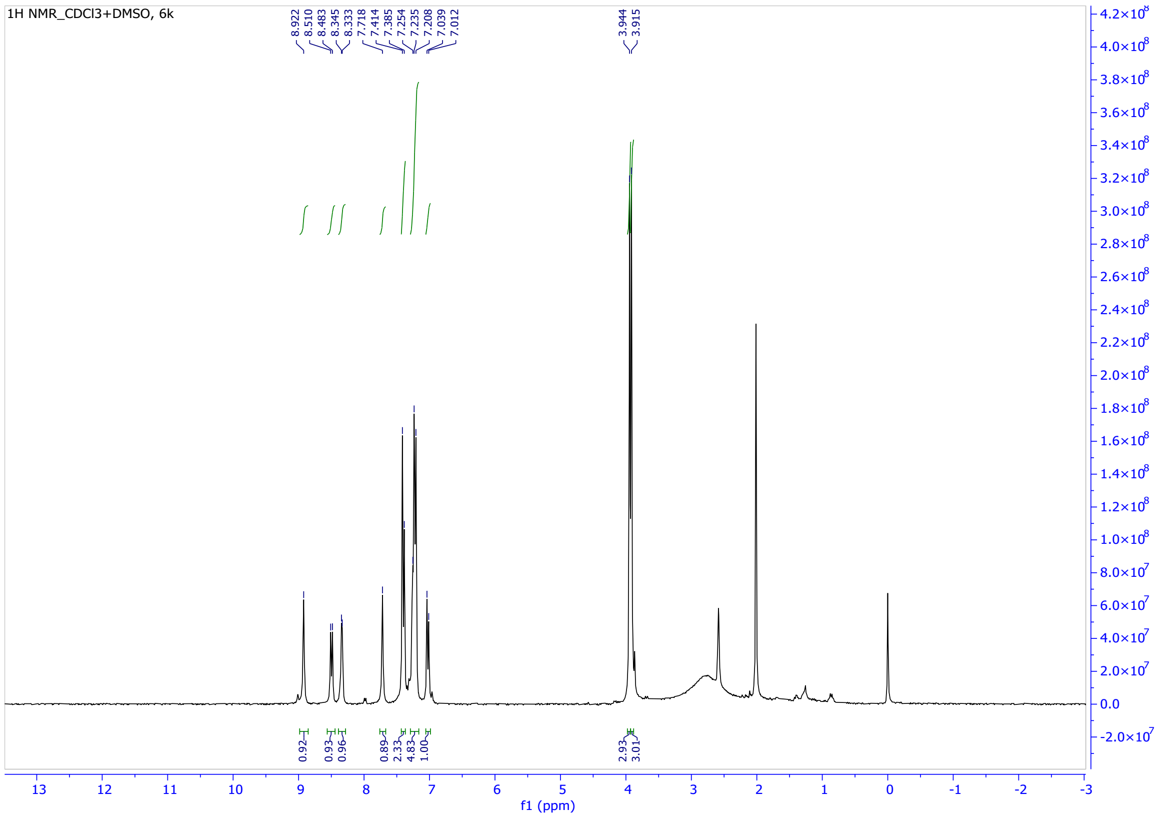

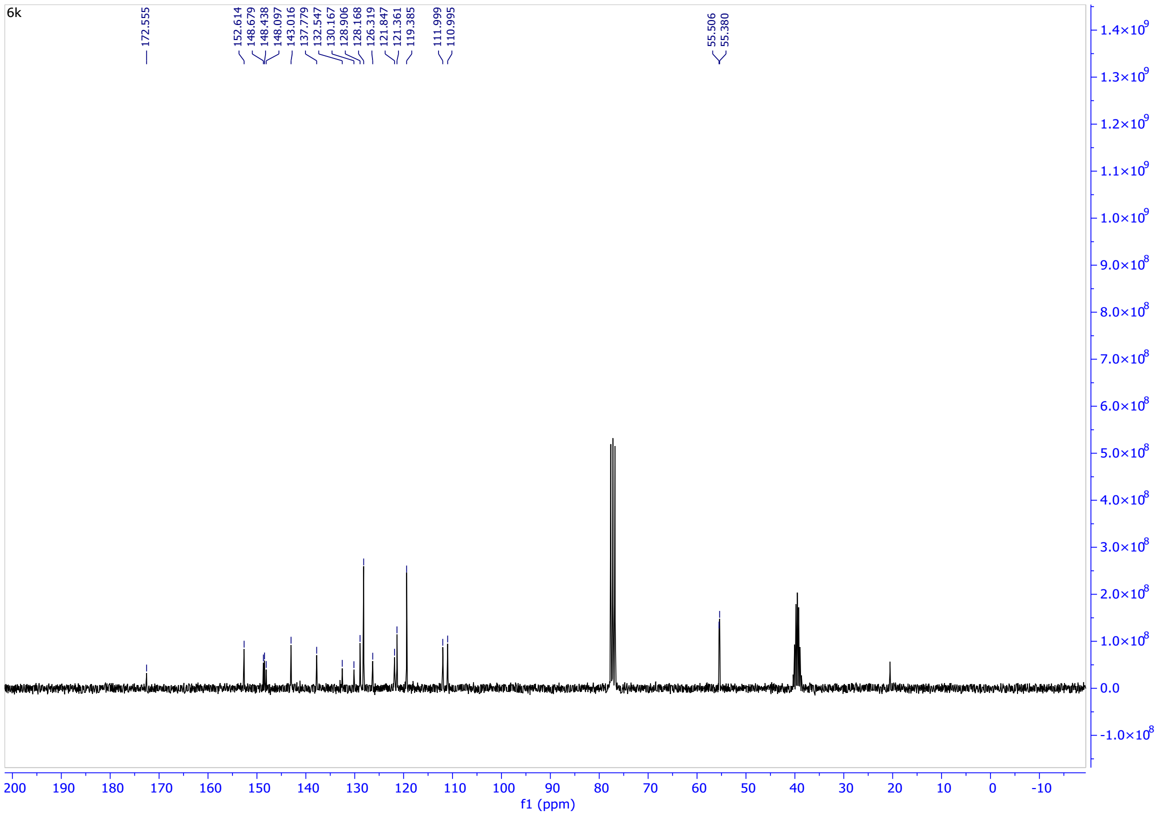

Compound **6l**

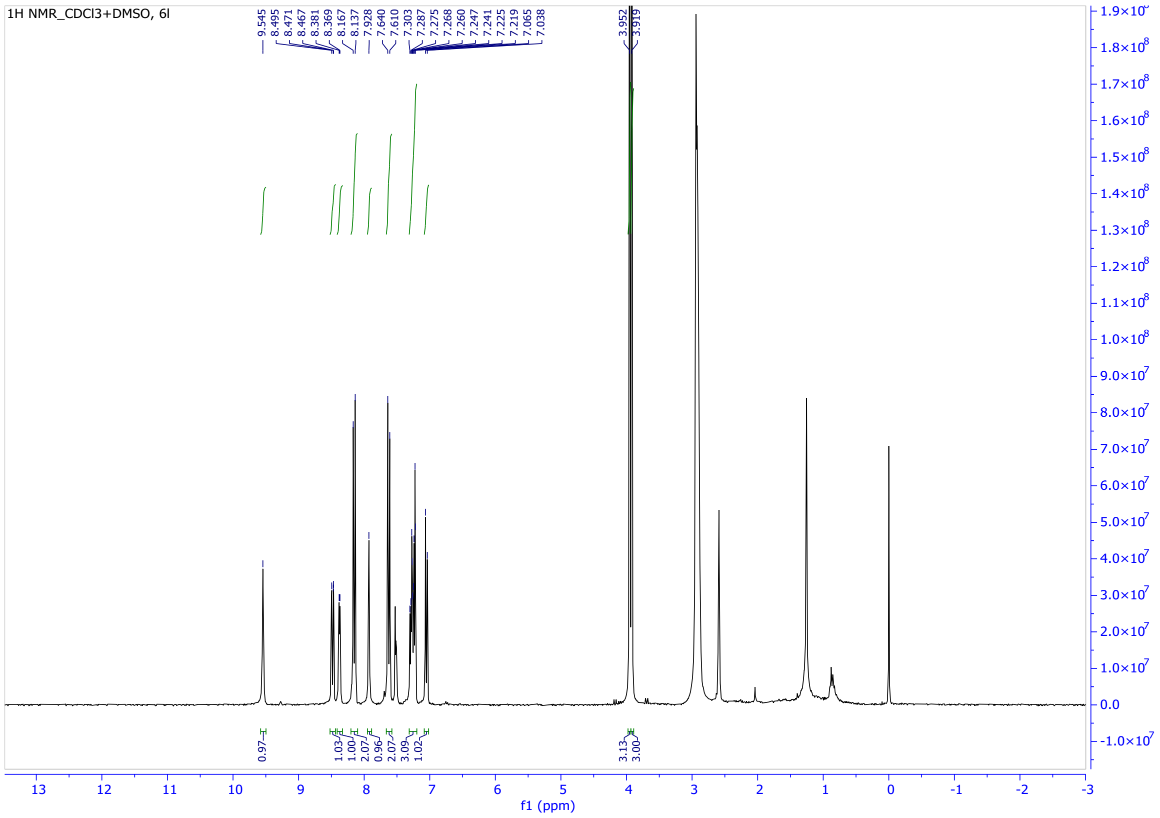
1H NMR_CDCl3+DMSO, 6l
9.545
8.495
8.471
8.467
8.381
8.369
8.167
8.137
7.928
7.640
7.610
7.303
7.287
7.275
7.268
7.260
7.247
7.241
7.225
7.219
7.065
7.038
3.952
3.919
0.97
1.03
1.00
2.07
0.96
2.07
3.09
1.02
3.13
3.00
f1 (ppm)

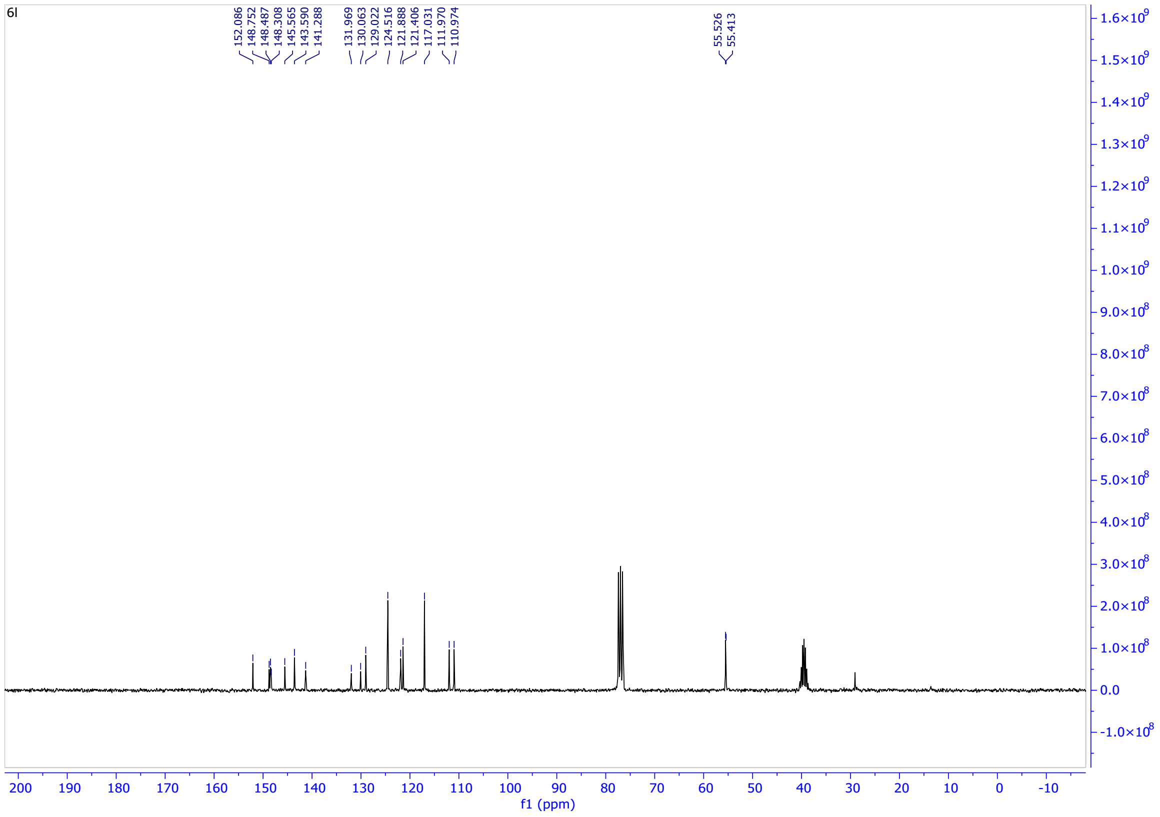
6l
152.086
148.752
148.487
148.308
145.565
143.590
141.288
131.969
130.063
129.022
124.516
121.888
121.406
117.031
111.970
110.974
55.526
55.413
f1 (ppm)

Compound **6m**

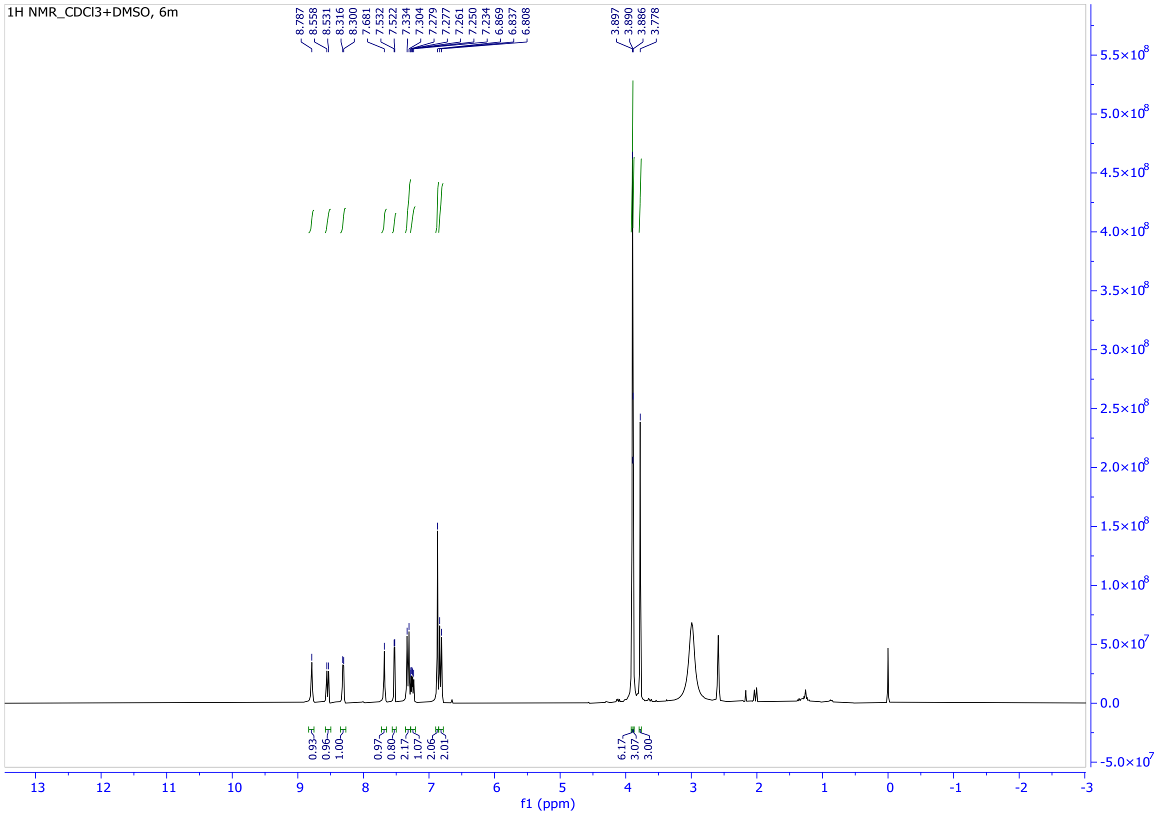
1H NMR_CDCl3+DMSO, 6m
8.787
8.558
8.531
8.316
8.300
7.681
7.532
7.522
7.334
7.304
7.279
7.277
7.261
7.250
7.234
6.869
6.837
6.808
3.897
3.890
3.886
3.778
0.93
0.96
1.00
0.97
0.80
2.17
1.07
2.06
2.01
6.17
3.07
3.00
f1 (ppm)

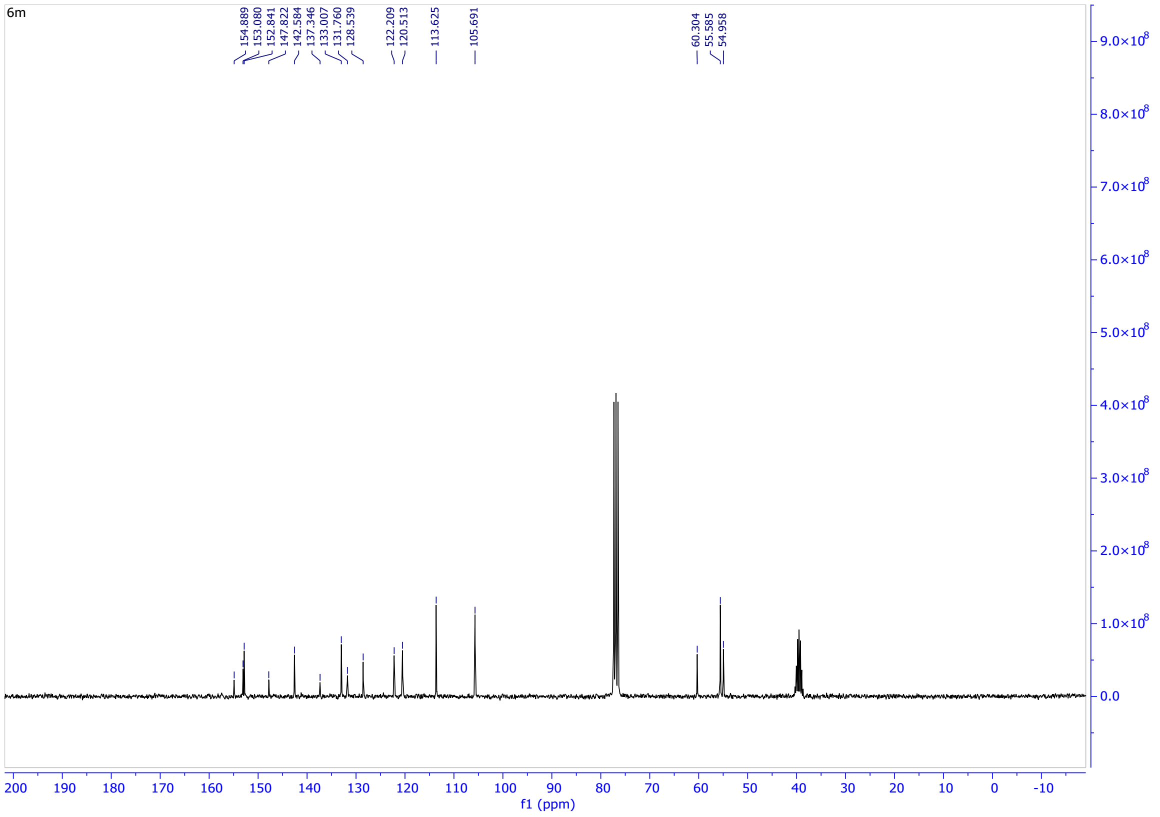
6m
154.889
153.080
152.841
147.822
142.584
137.346
133.007
131.760
128.539
122.209
120.513
113.625
105.691
60.304
55.585
54.958
f1 (ppm)

Compound **6n**

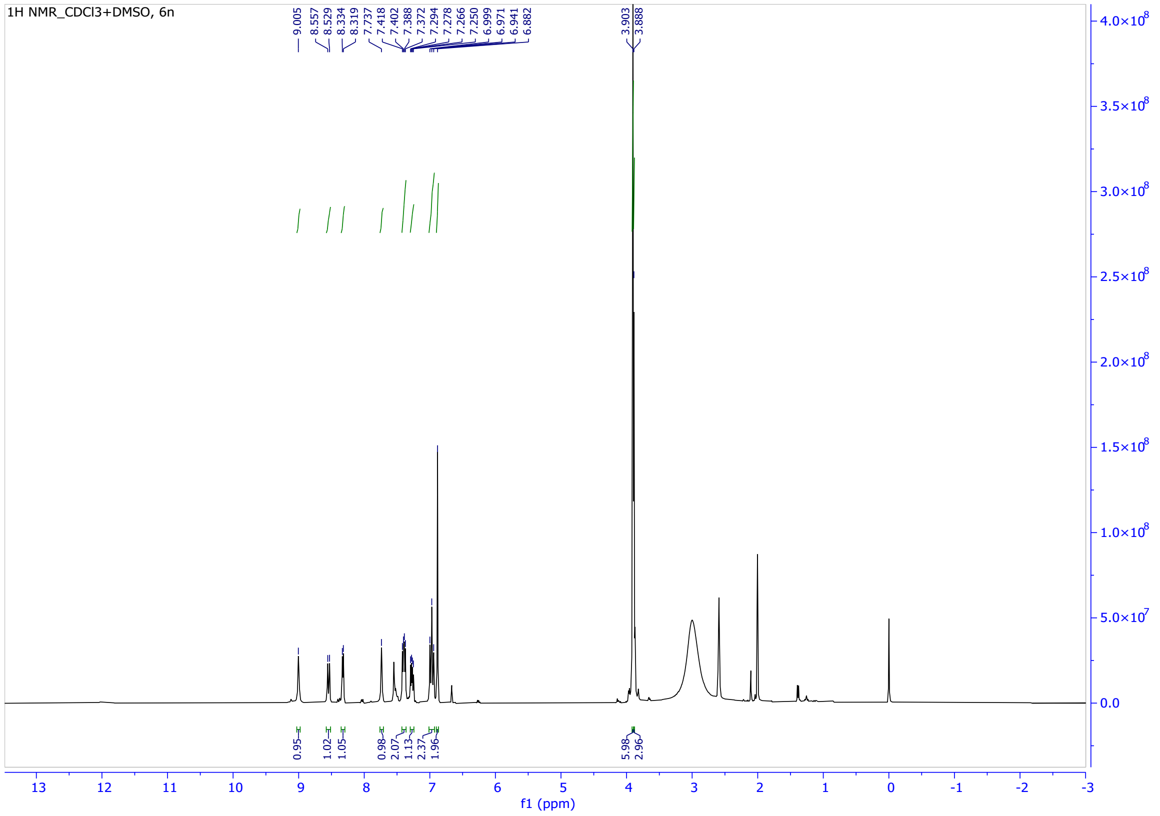
1H NMR_CDCl3+DMSO, 6n
9.005
8.557
8.529
8.334
8.319
7.737
7.418
7.402
7.388
7.372
7.294
7.278
7.266
7.250
6.999
6.971
6.941
6.882
3.903
3.888
0.95
1.02
1.05
0.98
2.07
1.13
2.37
1.96
5.98
2.96
f1 (ppm)

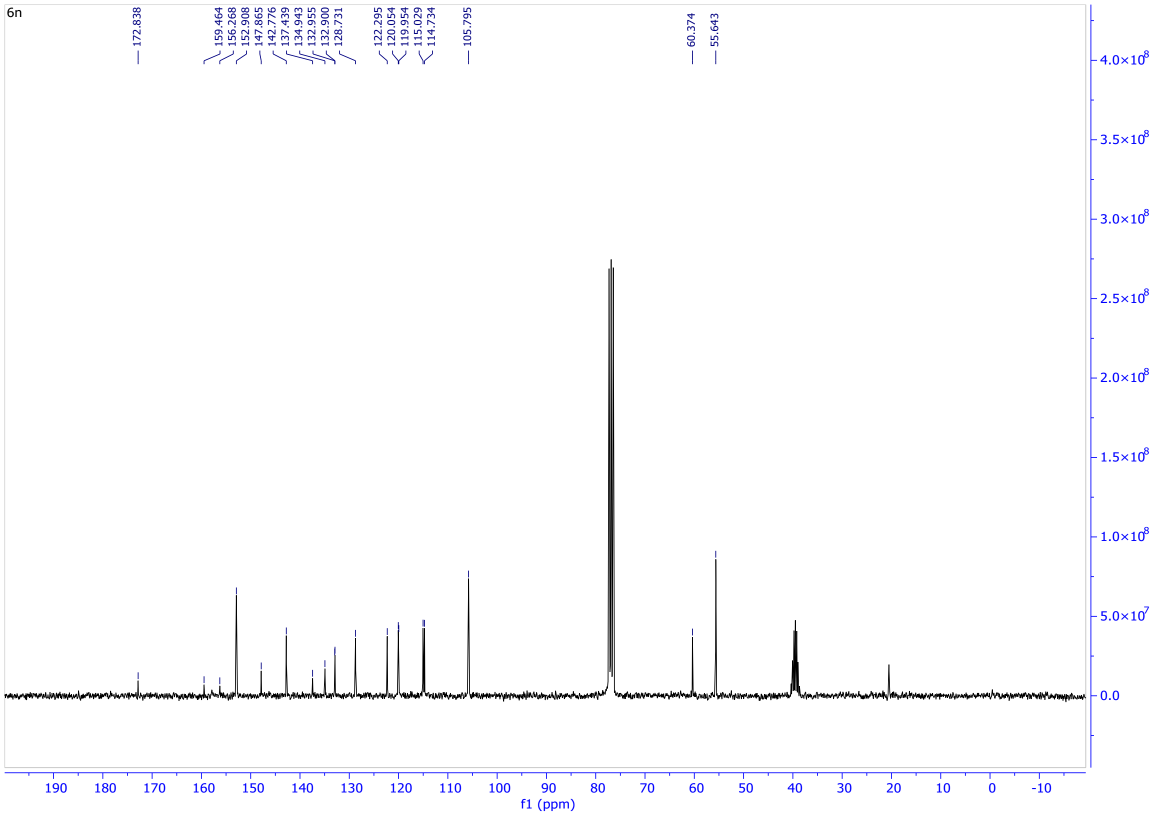
6n
172.838
159.464
156.268
152.908
147.865
142.776
137.439
134.943
132.955
132.900
128.731
122.295
120.054
119.954
115.029
114.734
105.795
60.374
55.643
f1 (ppm)

Compound **6p**

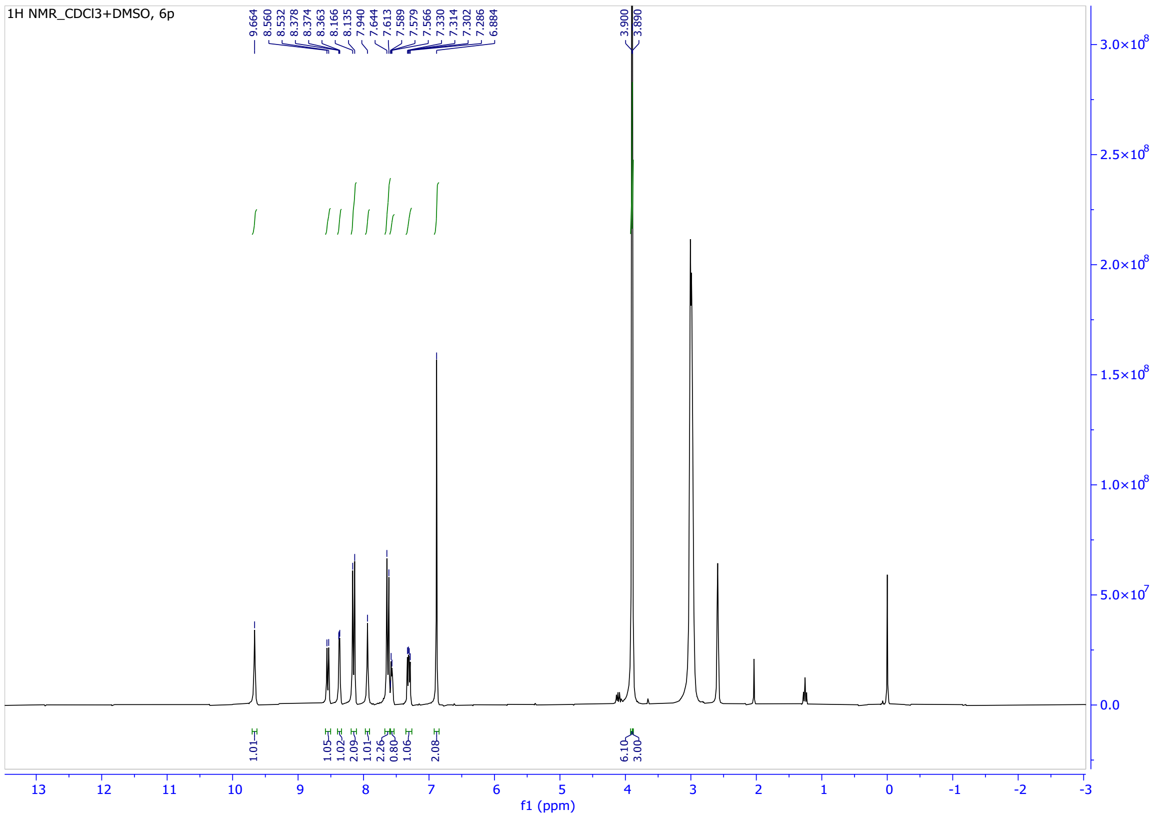
1H NMR_CDCl3+DMSO, 6p
9.664
8.560
8.532
8.378
8.374
8.363
8.166
8.135
7.940
7.644
7.613
7.589
7.579
7.566
7.330
7.314
7.302
7.286
6.884
3.900
3.890
1.01
1.05
1.02
2.09
1.01
2.26
0.80
1.06
2.08
6.10
3.00
f1 (ppm)

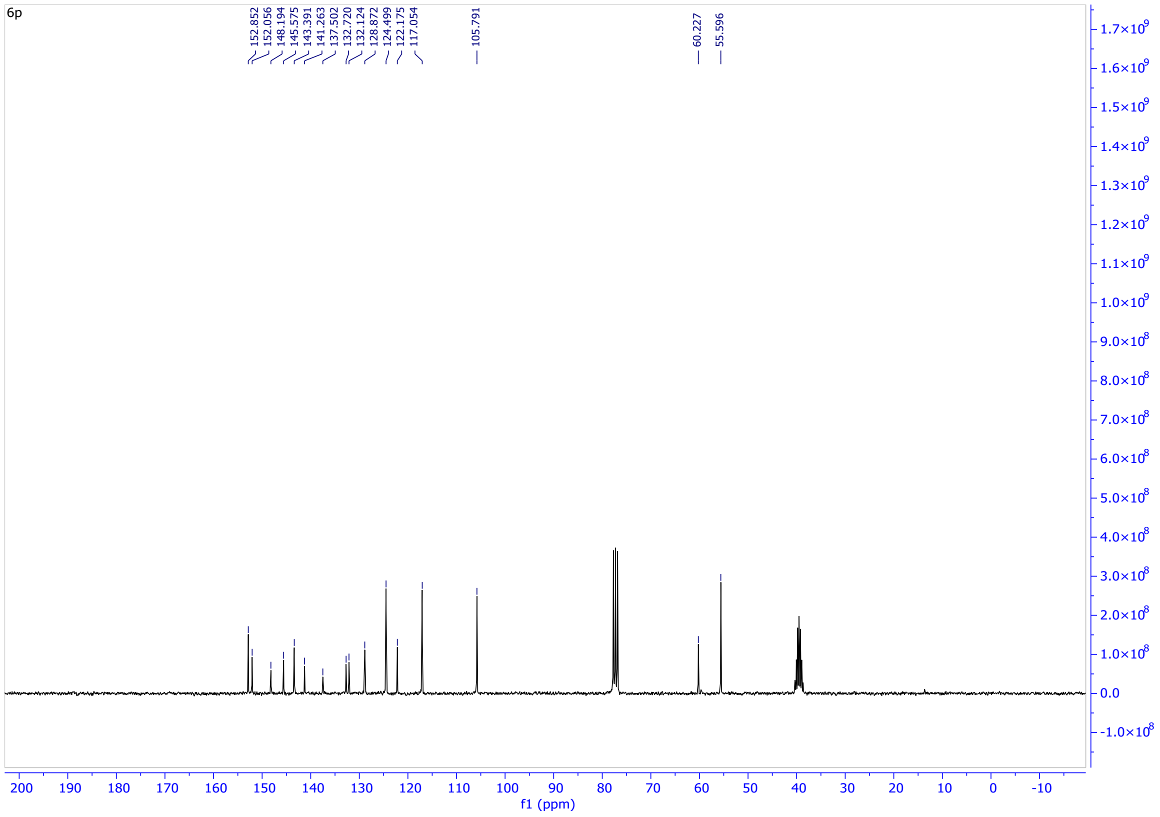
6p
152.852
152.056
148.194
145.575
143.391
141.263
137.502
132.720
132.124
128.872
124.499
122.175
117.054
105.791
60.227
55.596
f1 (ppm)

Compound **6q**

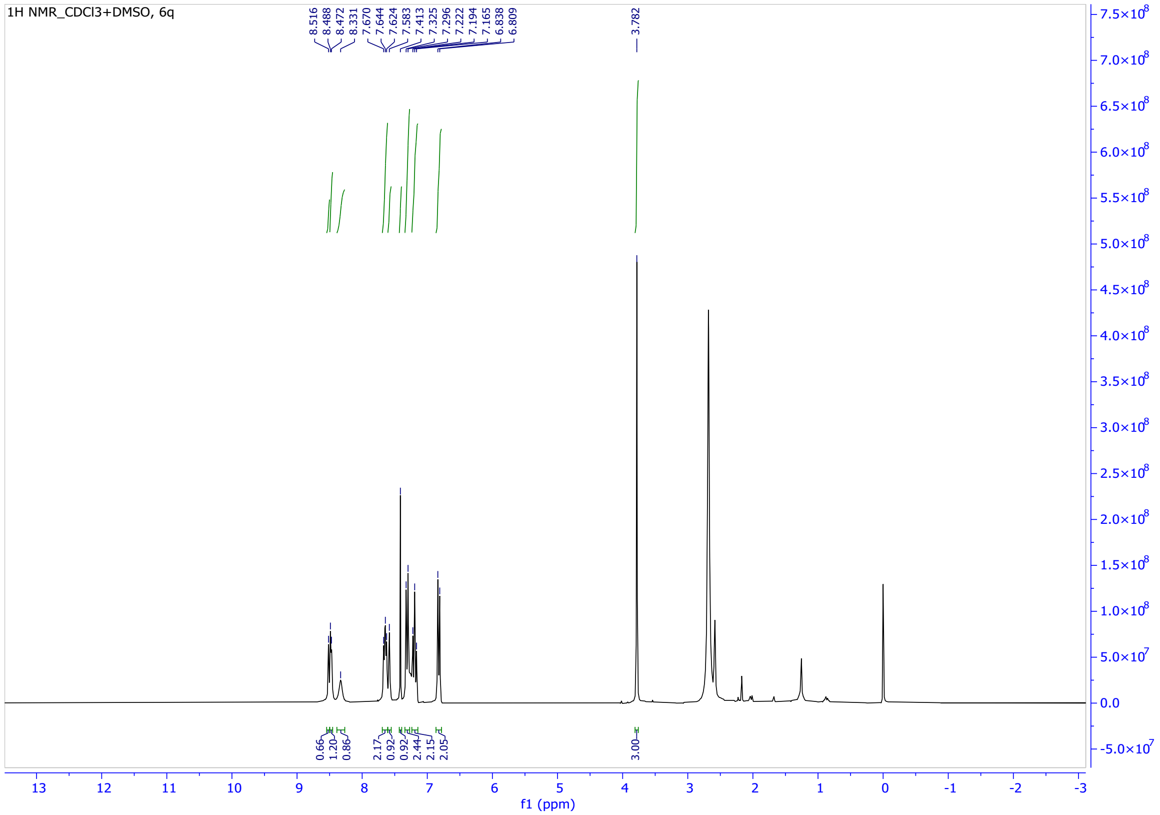
1H NMR_CDCl3+DMSO, 6q
8.516
8.488
8.472
8.331
7.670
7.644
7.624
7.583
7.413
7.325
7.296
7.222
7.194
7.165
6.838
6.809
3.782
0.66
1.20
0.86
2.17
0.92
0.92
2.44
2.15
2.05
3.00
f1 (ppm)

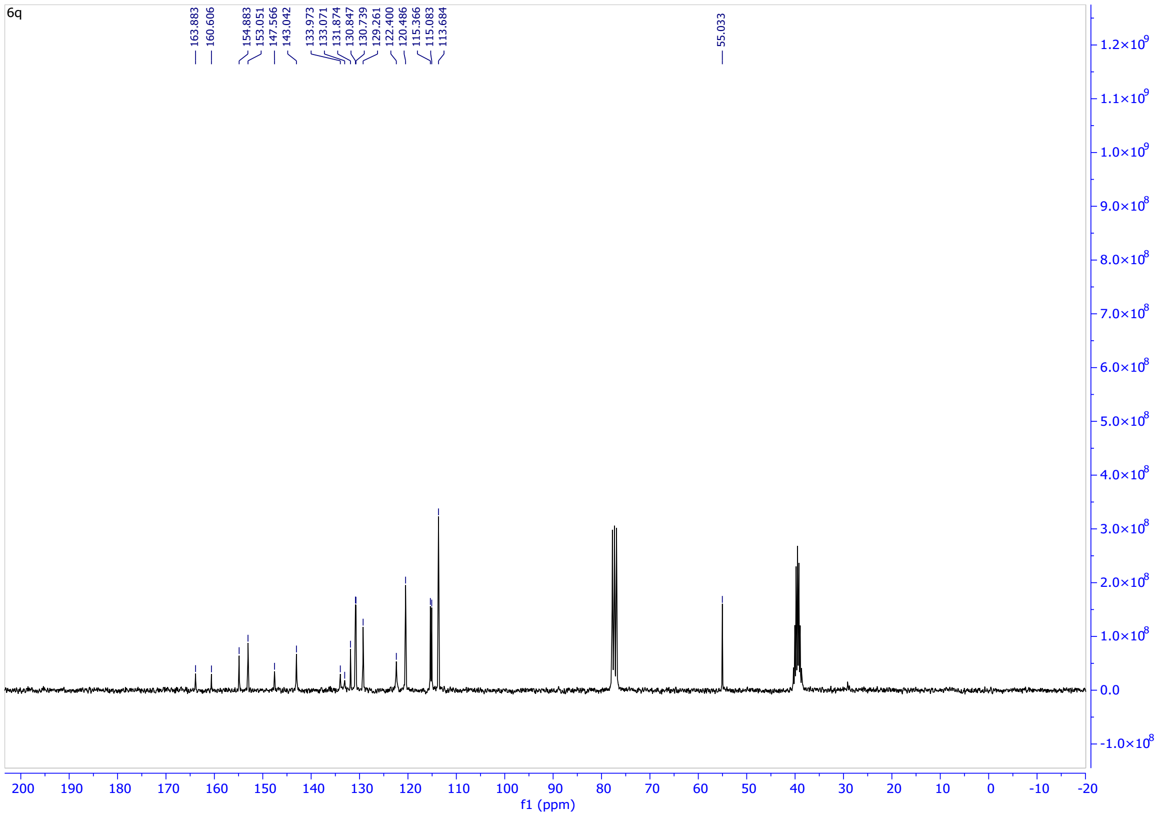
6q
163.883
160.606
154.883
153.051
147.566
143.042
133.973
133.071
131.874
130.847
130.739
129.261
122.400
120.486
115.366
115.083
113.684
55.033
f1 (ppm)

Compound **6r**

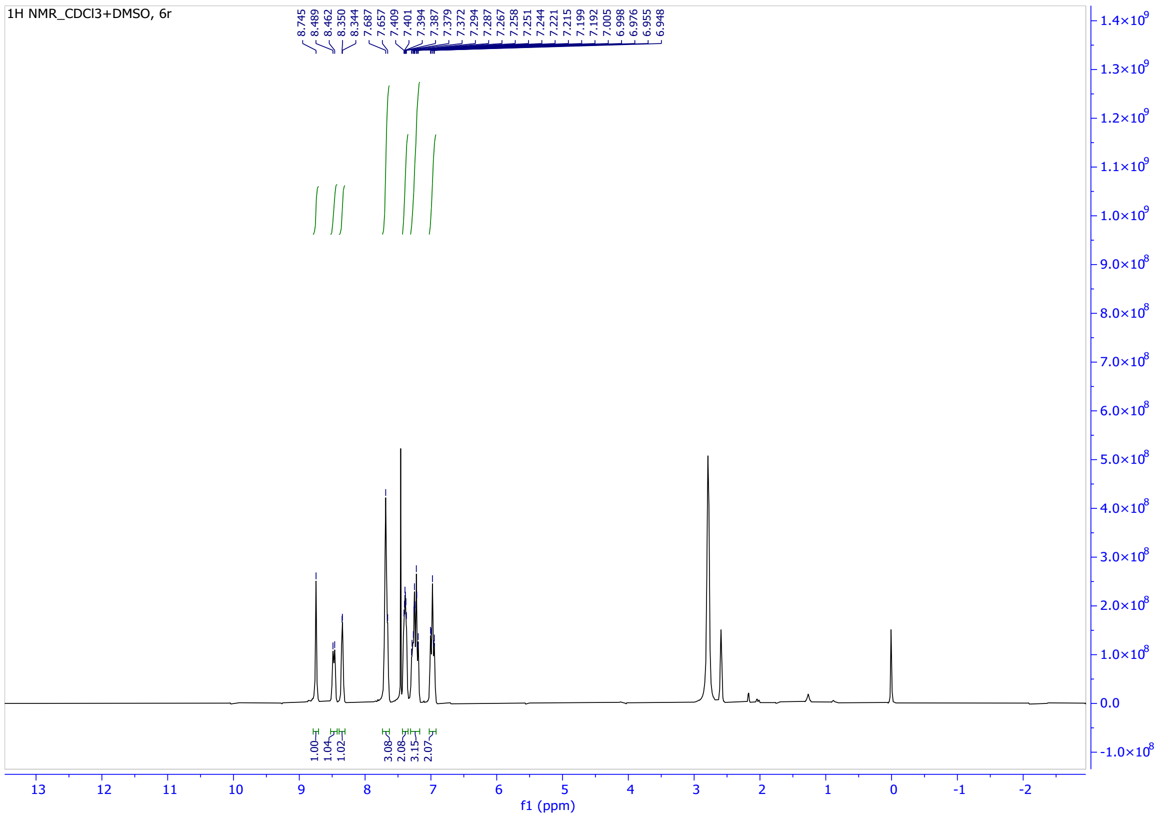

1H NMR_CDCl3+DMSO, 6r
8.745
8.489
8.462
8.350
8.344
7.687
7.657
7.409
7.401
7.394
7.387
7.379
7.372
7.294
7.287
7.267
7.258
7.251
7.244
7.221
7.215
7.199
7.192
7.005
6.998
6.976
6.955
6.948
1.00
1.04
1.02
3.08
2.08
3.15
2.07
f1 (ppm)

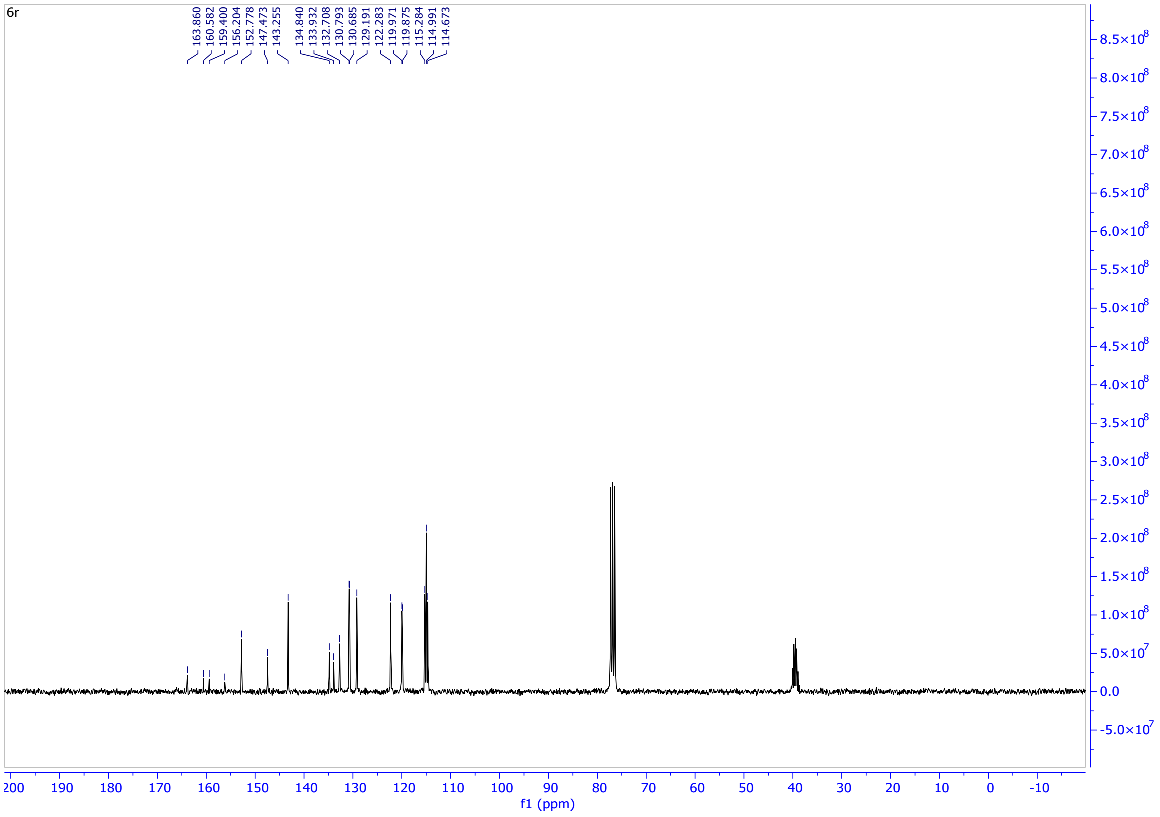

6r
163.860
160.582
159.400
156.204
152.778
147.473
143.255
134.840
133.932
132.708
130.793
130.685
129.191
122.283
119.971
119.875
115.284
114.991
114.673
f1 (ppm)

Compound **6s**

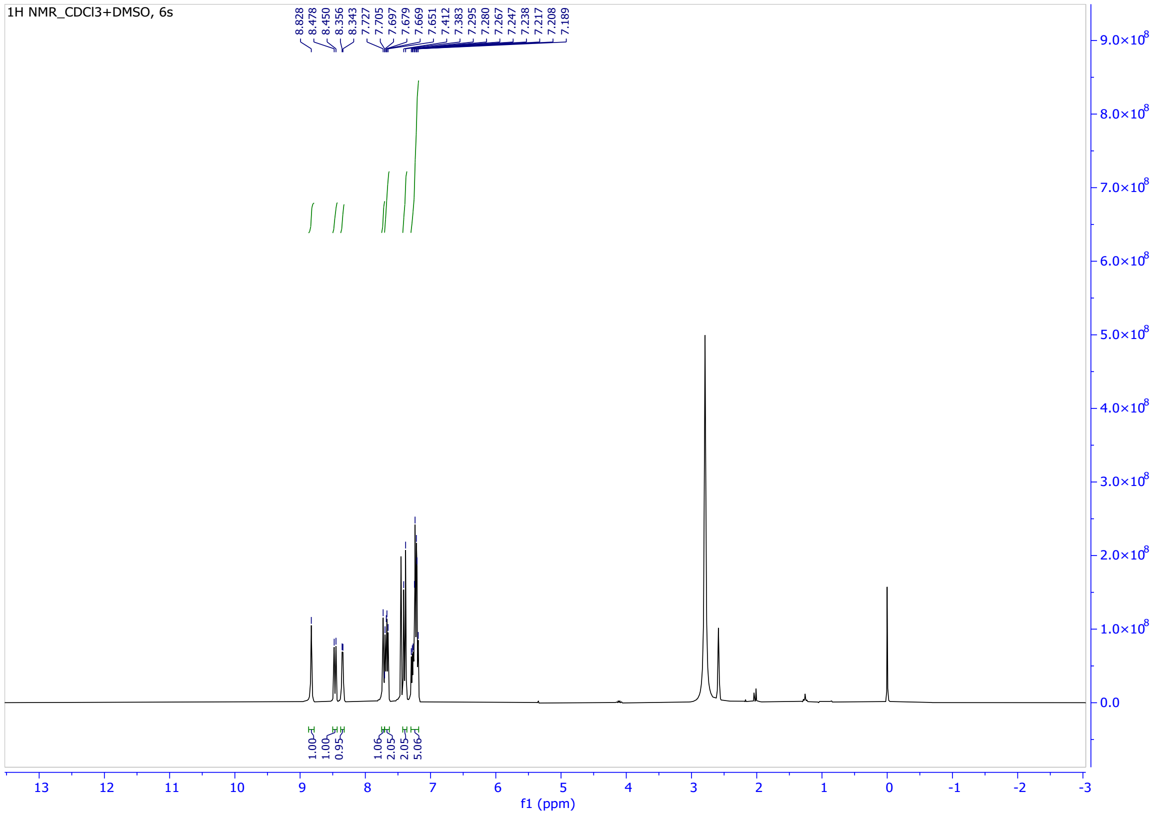
1H NMR_CDCl3+DMSO, 6s
8.828
8.478
8.450
8.356
8.343
7.727
7.705
7.697
7.679
7.669
7.651
7.412
7.383
7.295
7.280
7.267
7.247
7.238
7.217
7.208
7.189
1.00
1.00
0.95
1.06
2.05
2.05
5.06
f1 (ppm)

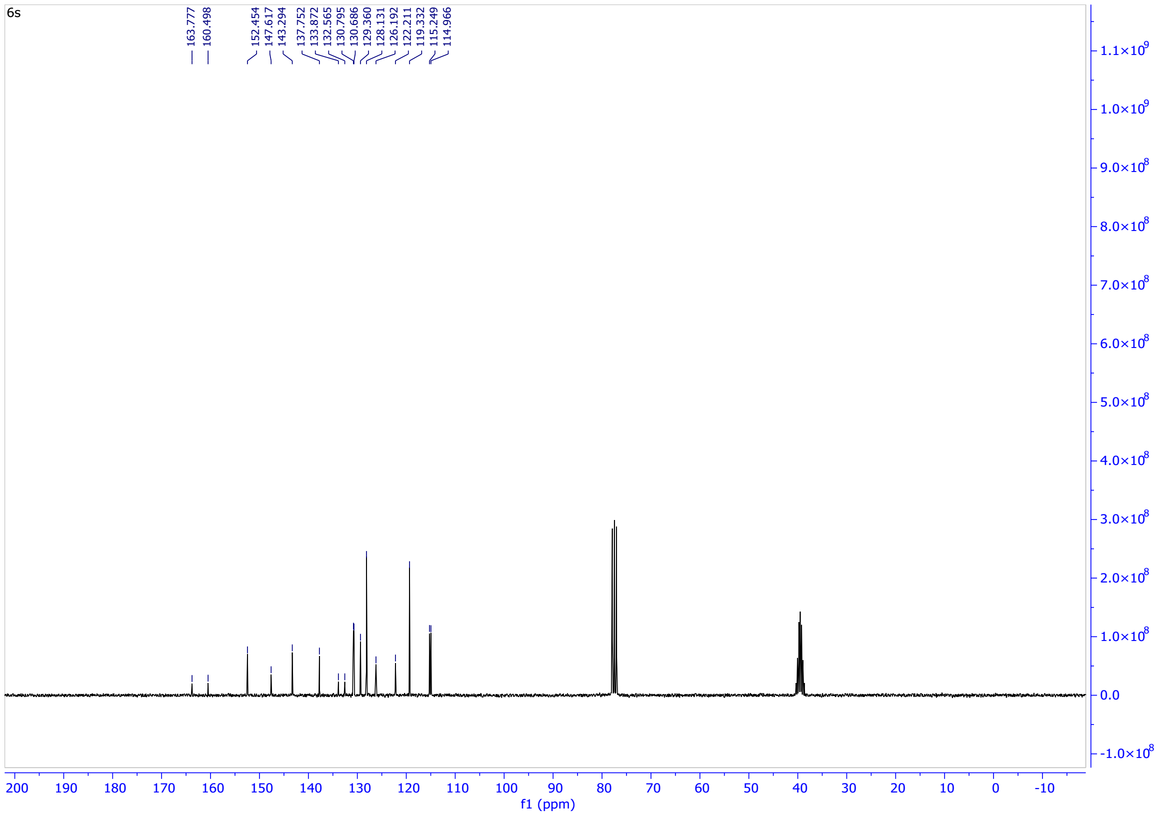
6s
163.777
160.498
152.454
147.617
143.294
137.752
133.872
132.565
130.795
130.686
129.360
128.131
126.192
122.211
119.332
115.249
114.966
f1 (ppm)

Compound **6t**

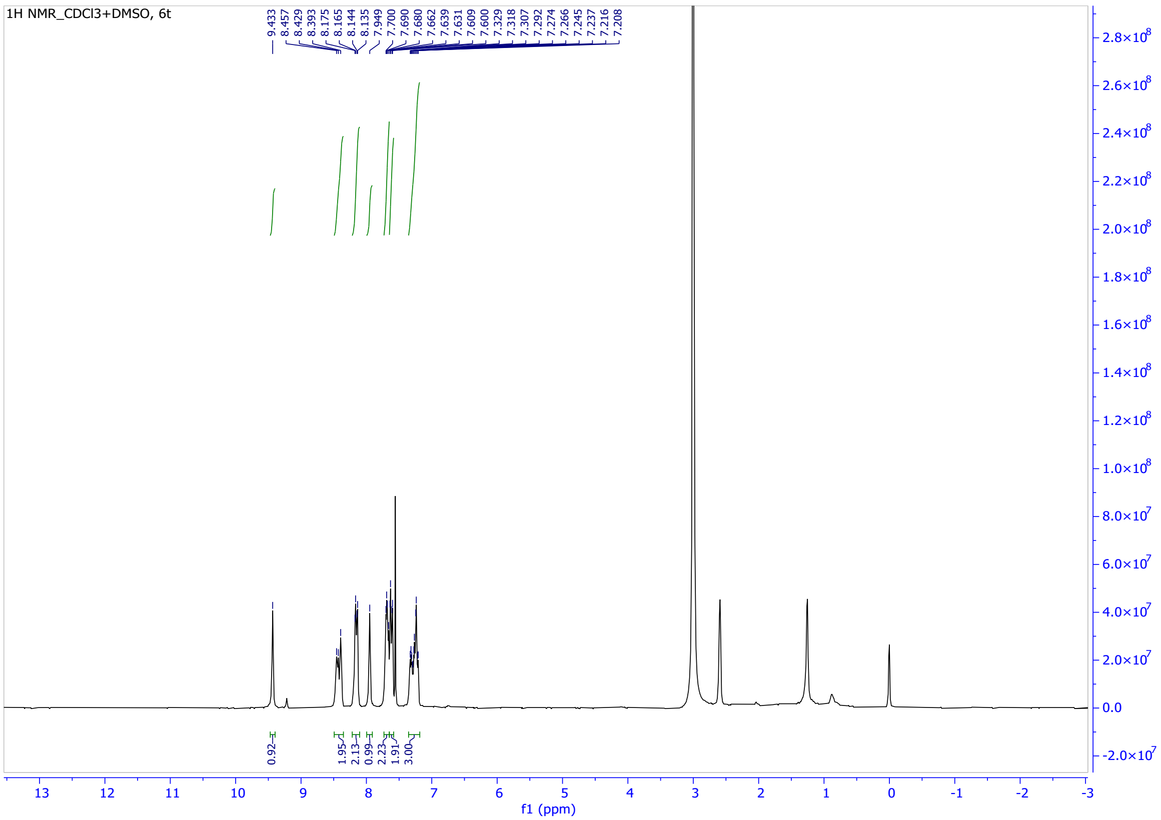
1H NMR_CDCl3+DMSO, 6t
9.433
8.457
8.429
8.393
8.175
8.165
8.144
8.135
7.949
7.700
7.690
7.680
7.662
7.639
7.631
7.609
7.600
7.329
7.318
7.307
7.292
7.274
7.266
7.245
7.237
7.216
7.208
0.92
1.95
2.13
0.99
2.23
1.91
3.00
f1 (ppm)

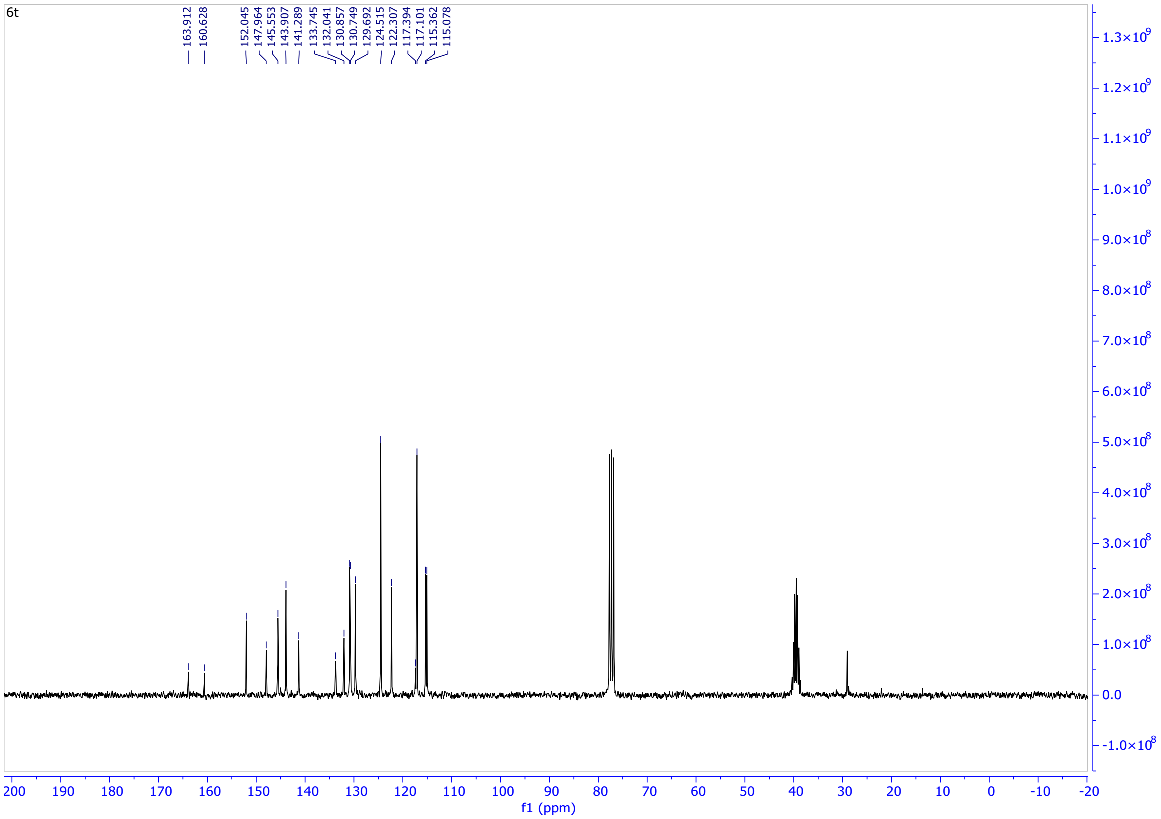
6t
163.912
160.628
152.045
147.964
145.553
143.907
141.289
133.745
132.041
130.857
130.749
129.692
124.515
122.307
117.394
117.101
115.362
115.078
f1 (ppm)

Compound **6u**

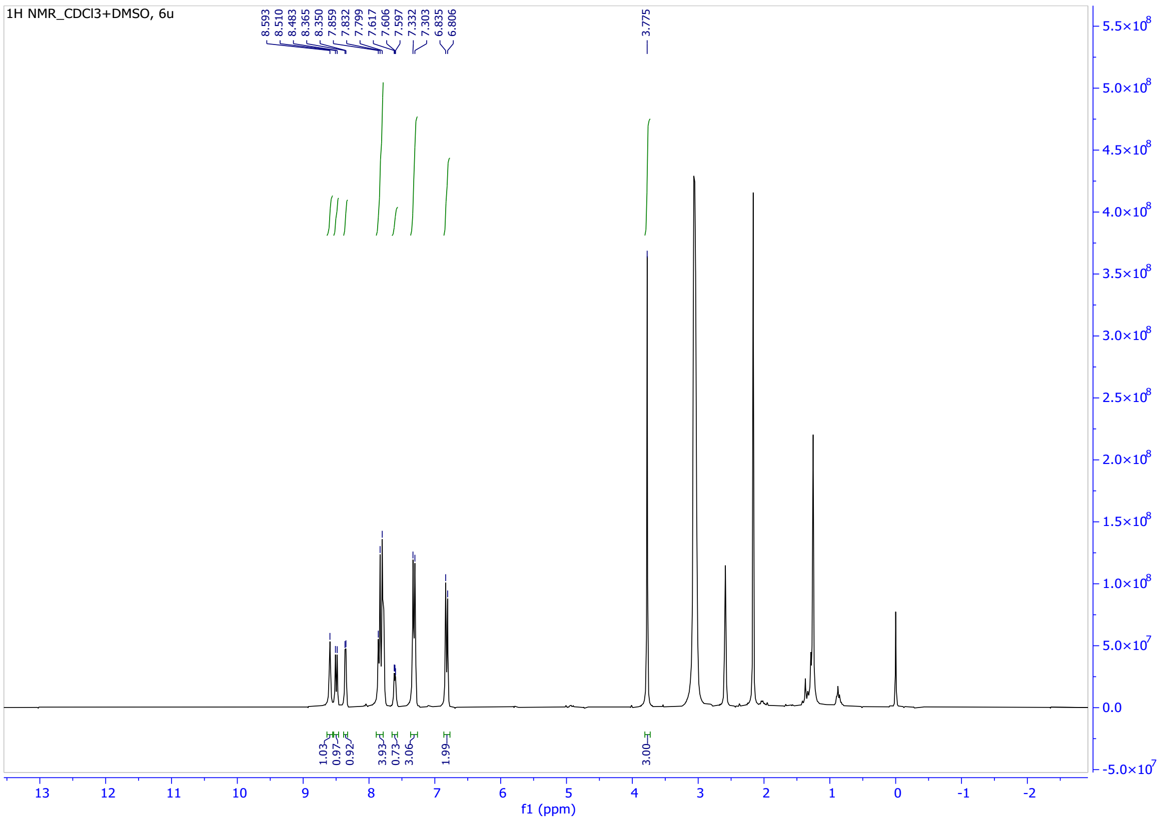
1H NMR_CDCl3+DMSO, 6u
8.593
8.510
8.483
8.365
8.350
7.859
7.832
7.799
7.617
7.606
7.597
7.332
7.303
6.835
6.806
3.775
1.03
0.97
0.92
3.93
0.73
3.06
1.99
3.00
f1 (ppm)

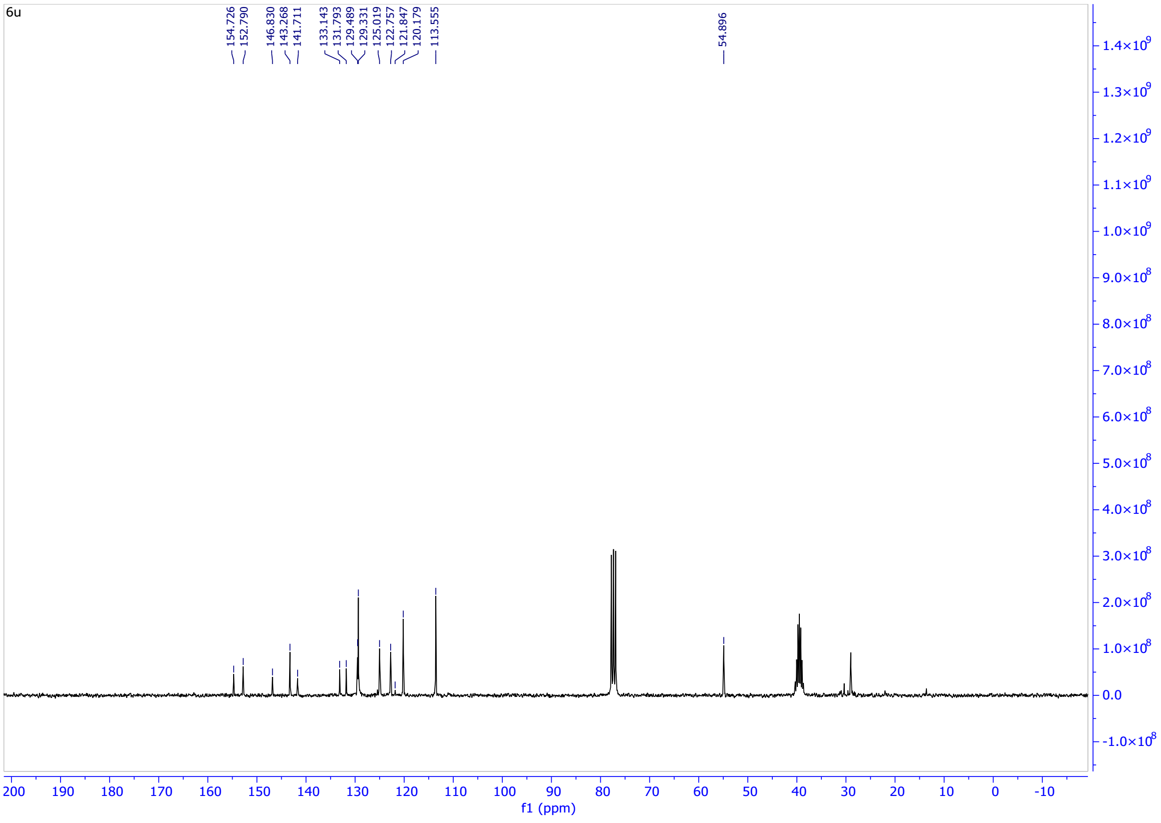
6u
154.726
152.790
146.830
143.268
141.711
133.143
131.793
129.489
129.331
125.019
122.757
121.847
120.179
113.555
54.896
f1 (ppm)

Compound **6v**

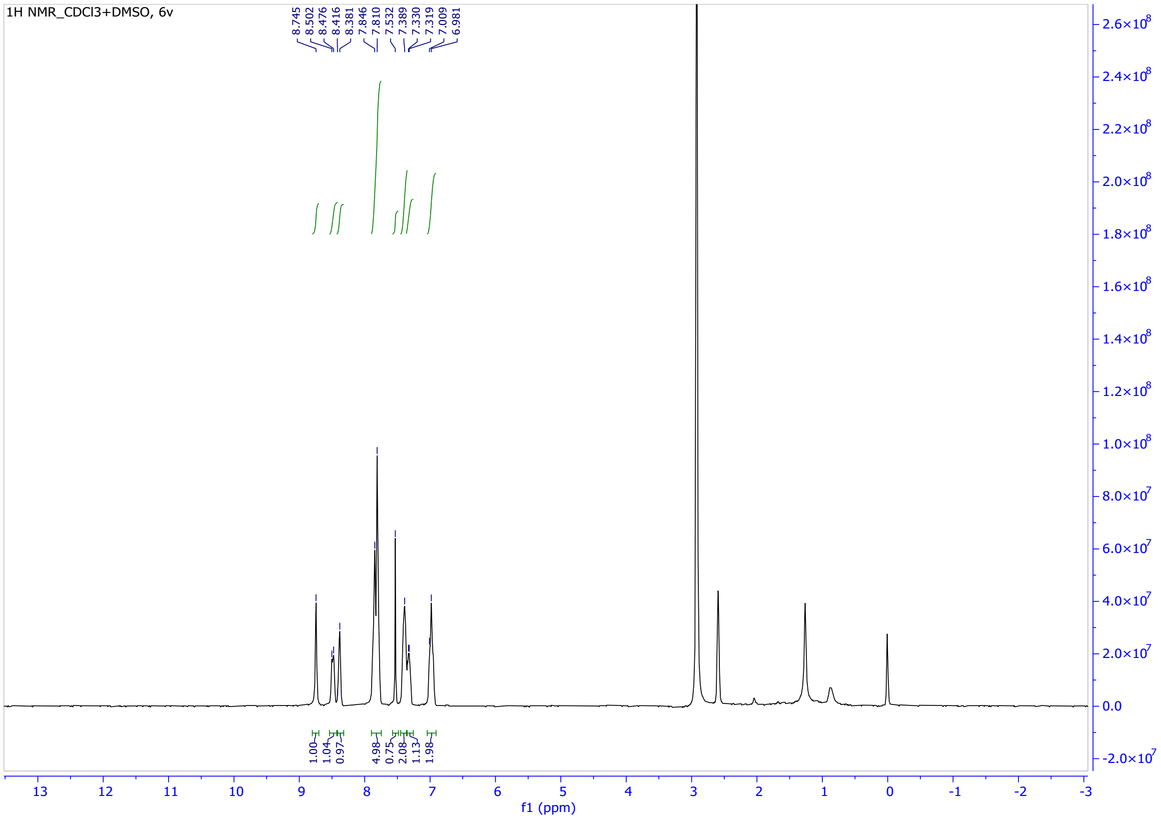
1H NMR_CDCl3+DMSO, 6v
8.745
8.502
8.476
8.416
8.381
7.846
7.810
7.532
7.389
7.330
7.319
7.009
6.981
1.00
1.04
0.97
4.98
0.75
2.08
1.13
1.98
f1 (ppm)

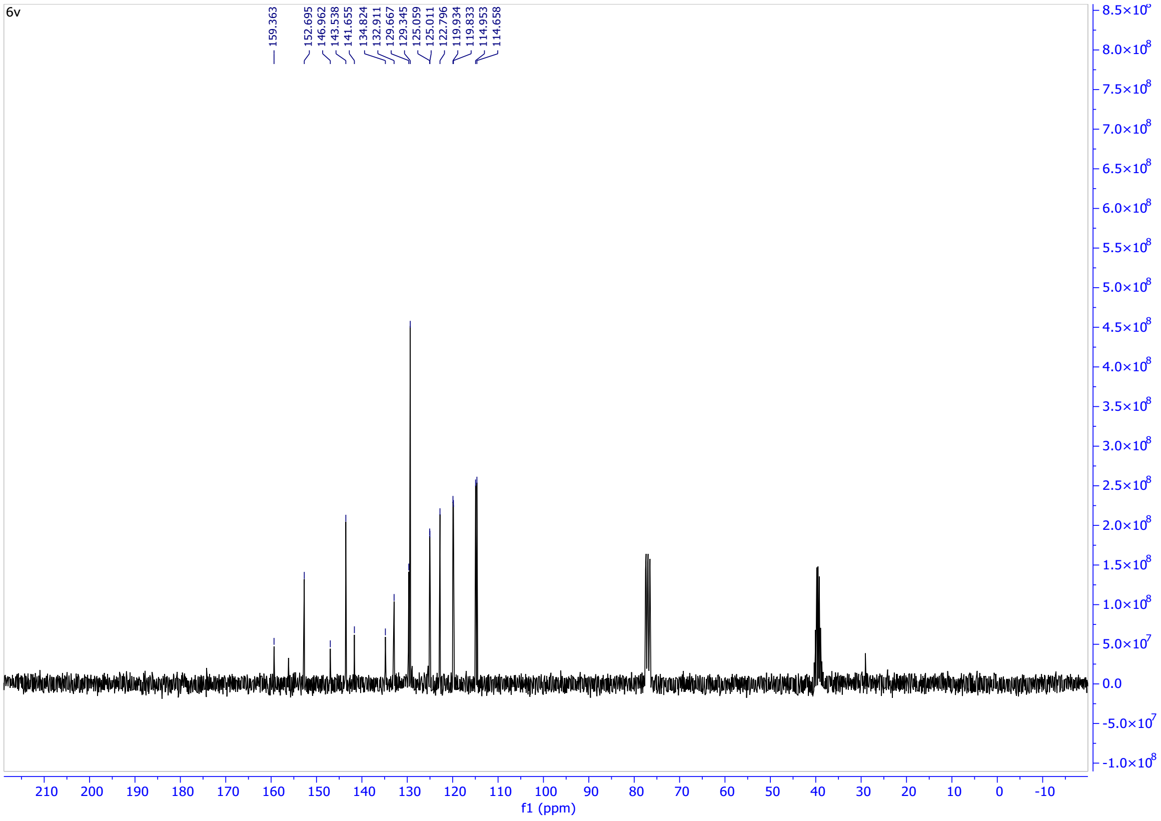
6v
159.363
152.695
146.962
143.538
141.655
134.824
132.911
129.667
129.345
125.059
125.011
122.796
119.934
119.833
114.953
114.658
f1 (ppm)

Compound **6w**

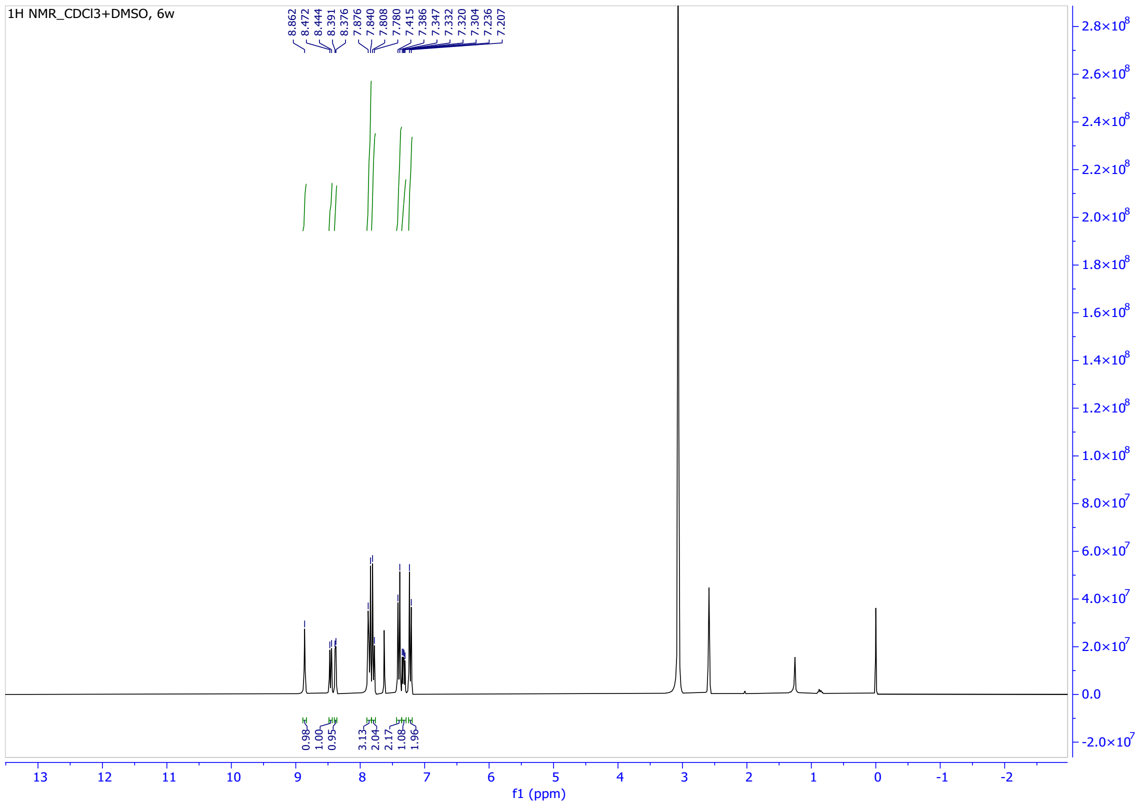

Compound **6x**

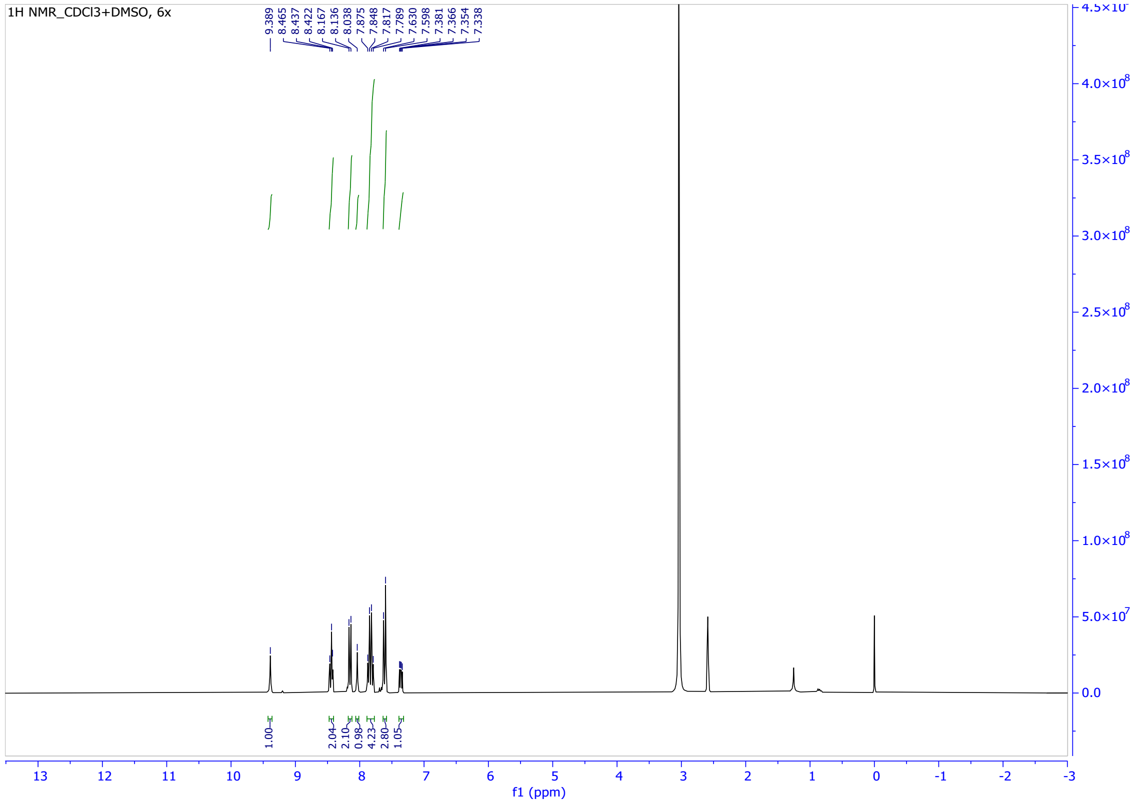

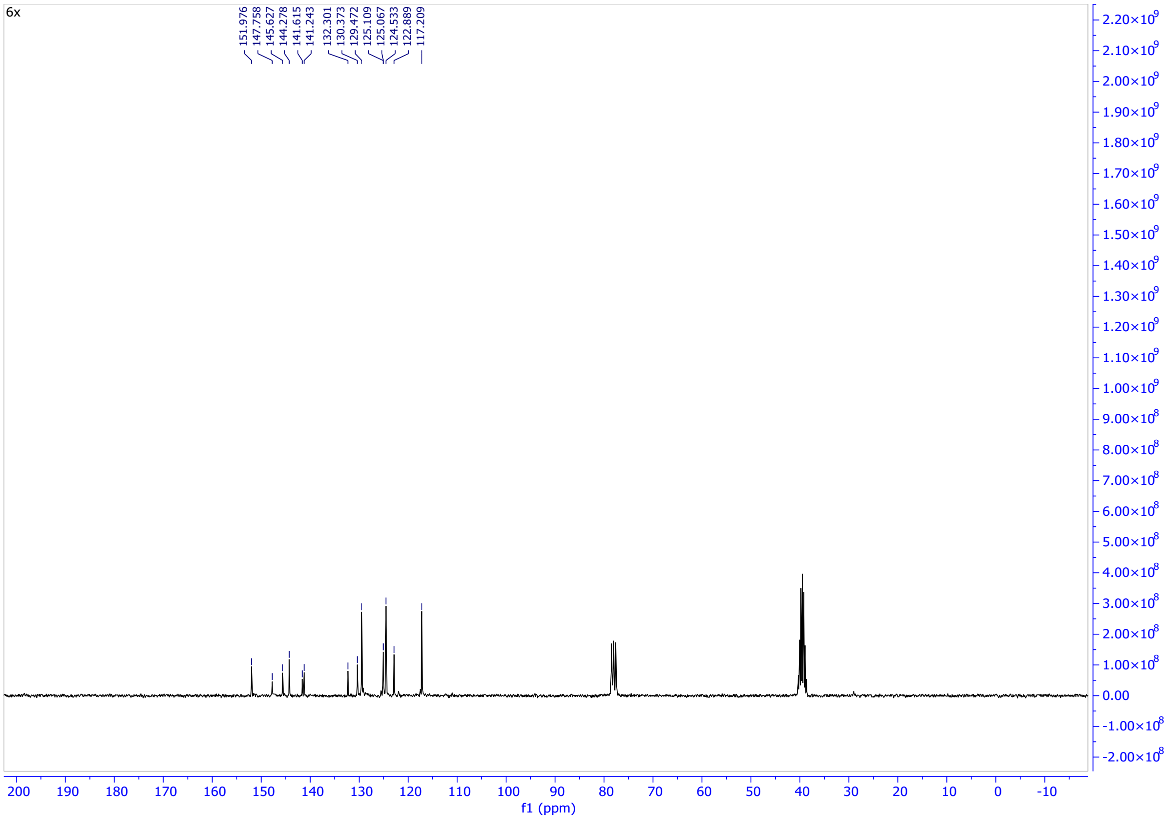
6x
151.976
147.758
145.627
144.278
141.615
141.243
132.301
130.373
129.472
125.109
125.067
124.533
122.889
117.209
f1 (ppm)

LC-MS Spectra

Compound **6a**

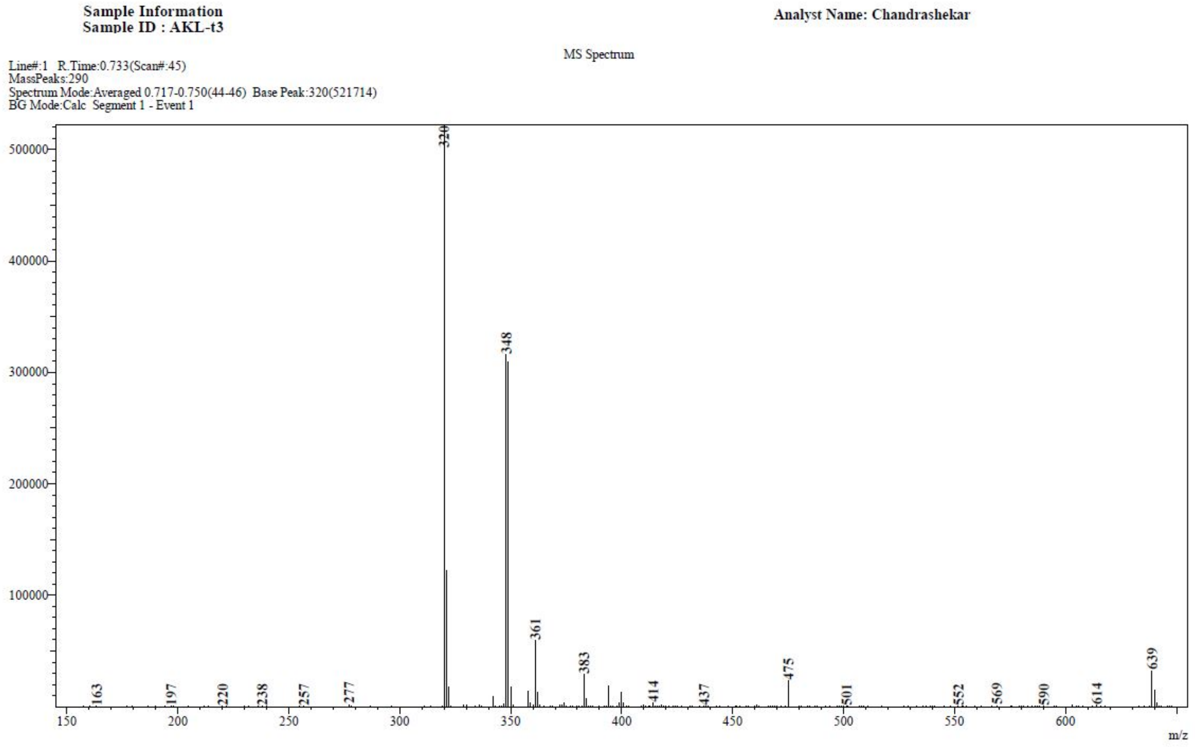

Compound **6b**

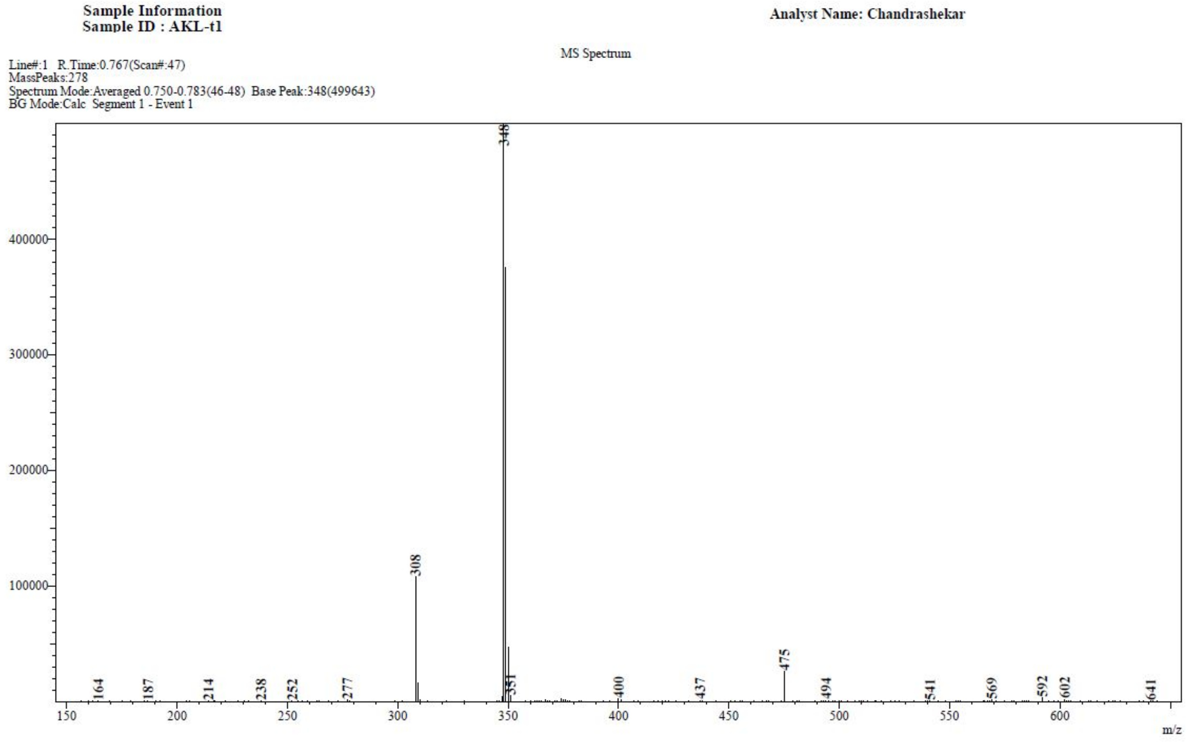

Compound **6c**

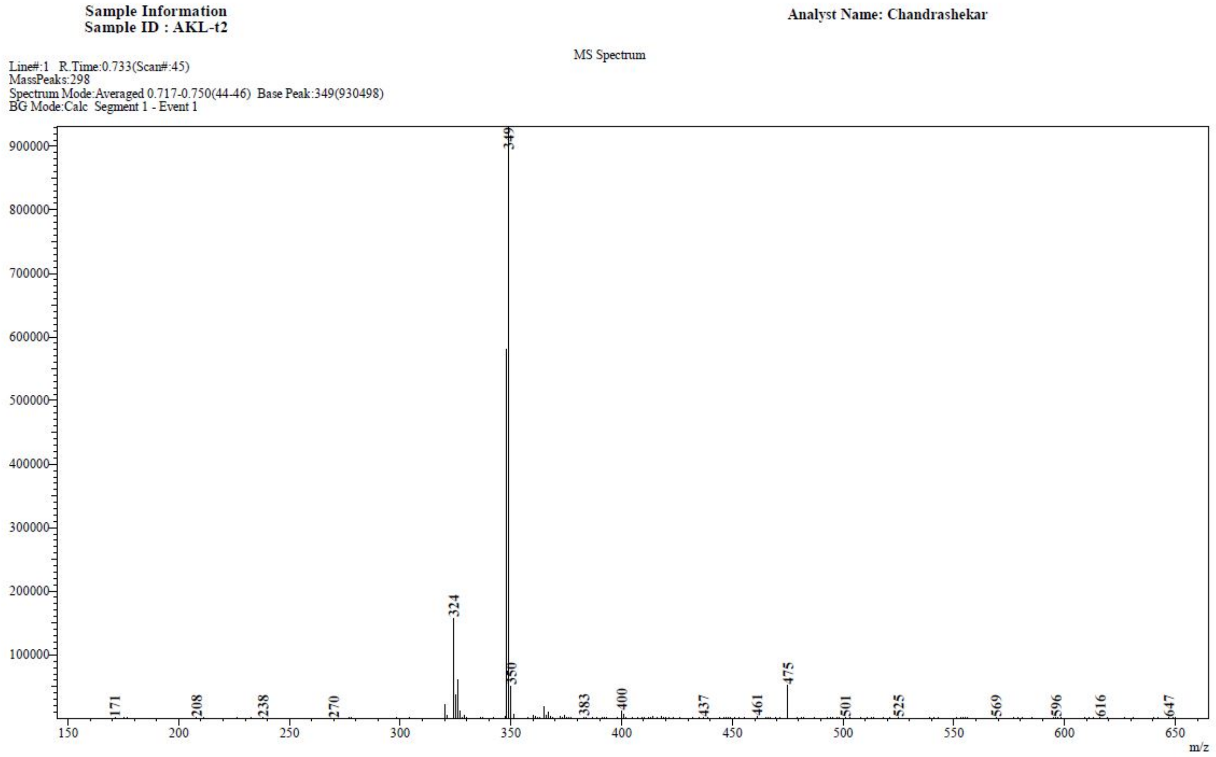

Compound **6d**

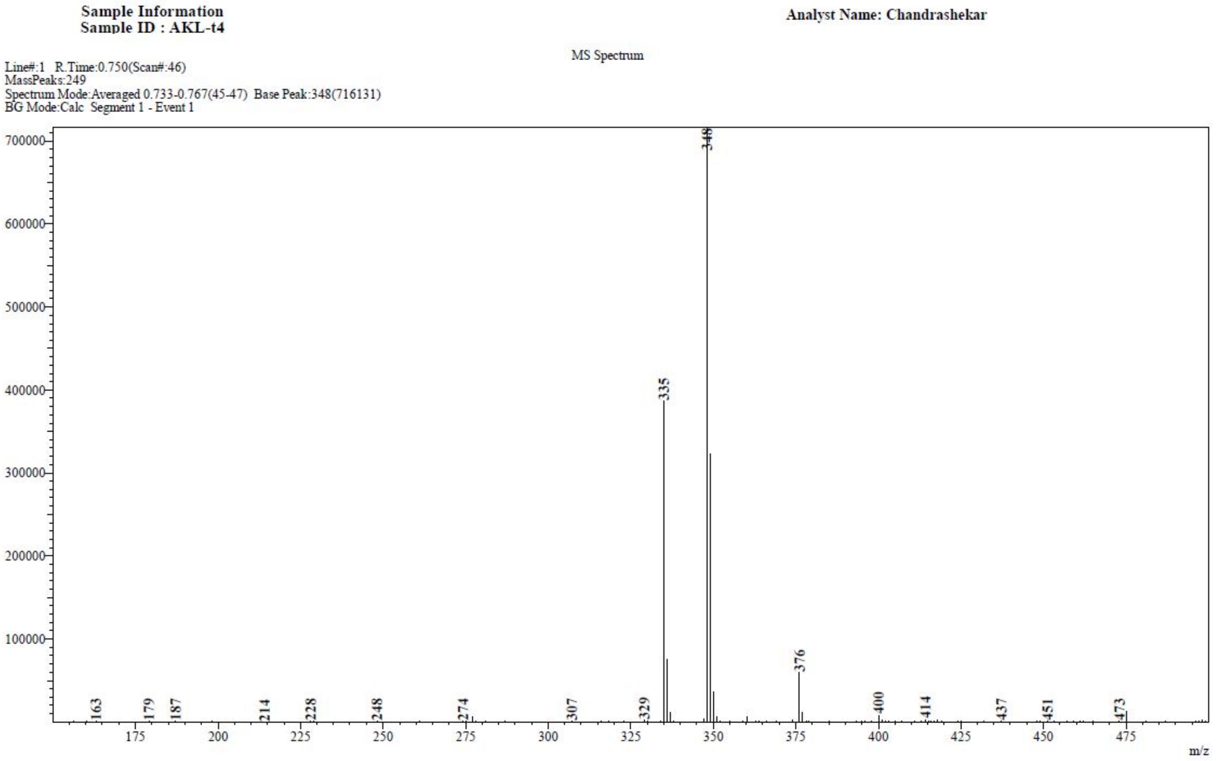

Compound **6e**

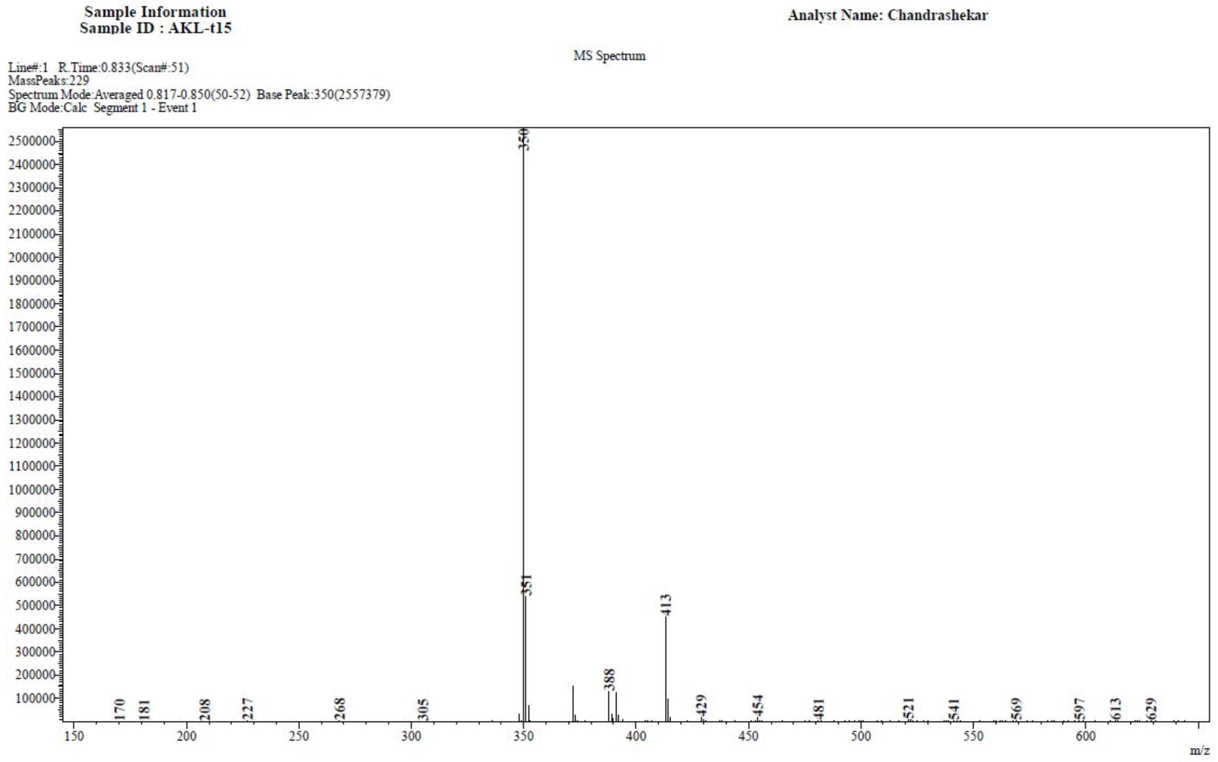

Compound **6f**

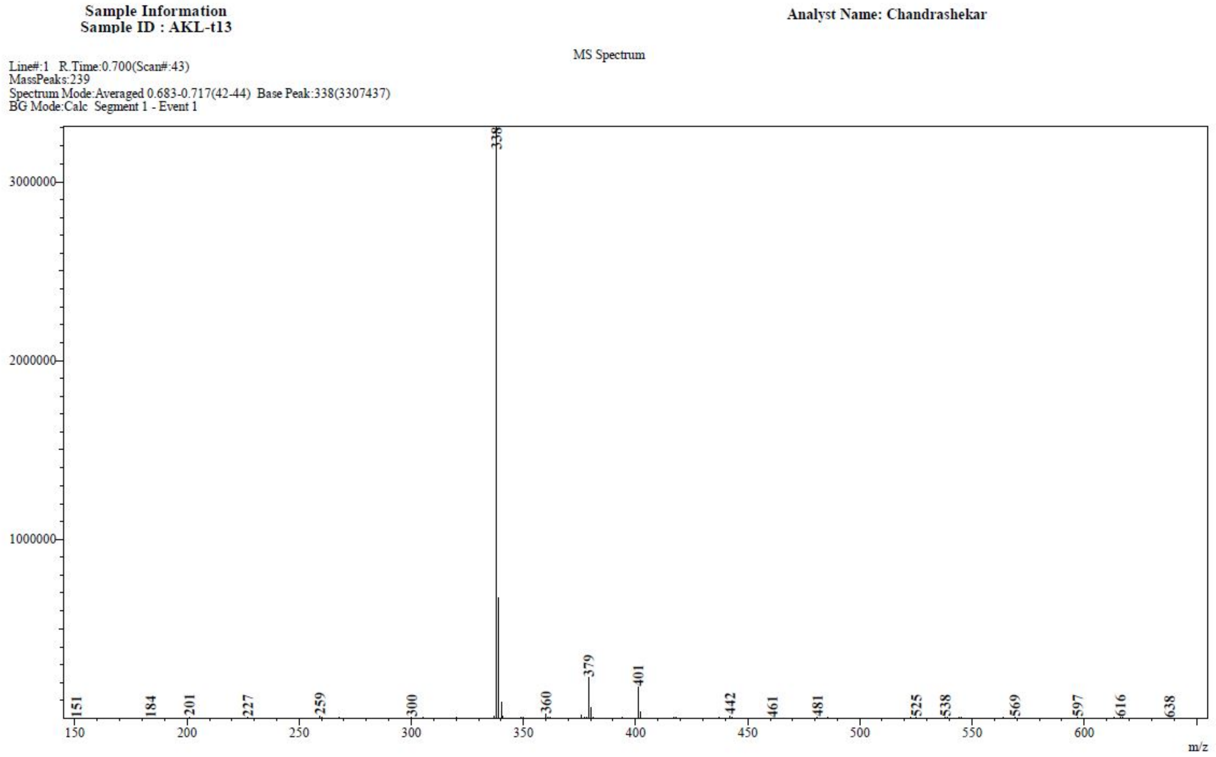

Compound **6g**

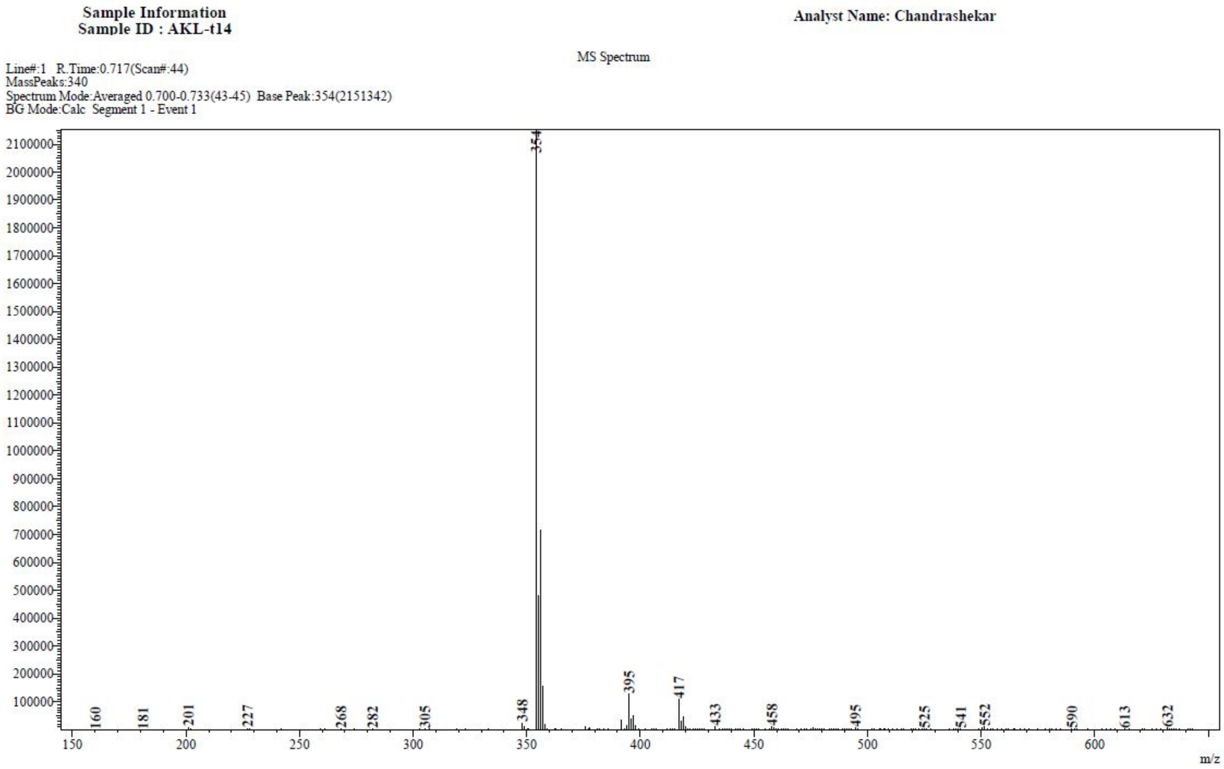

Compound **6h**

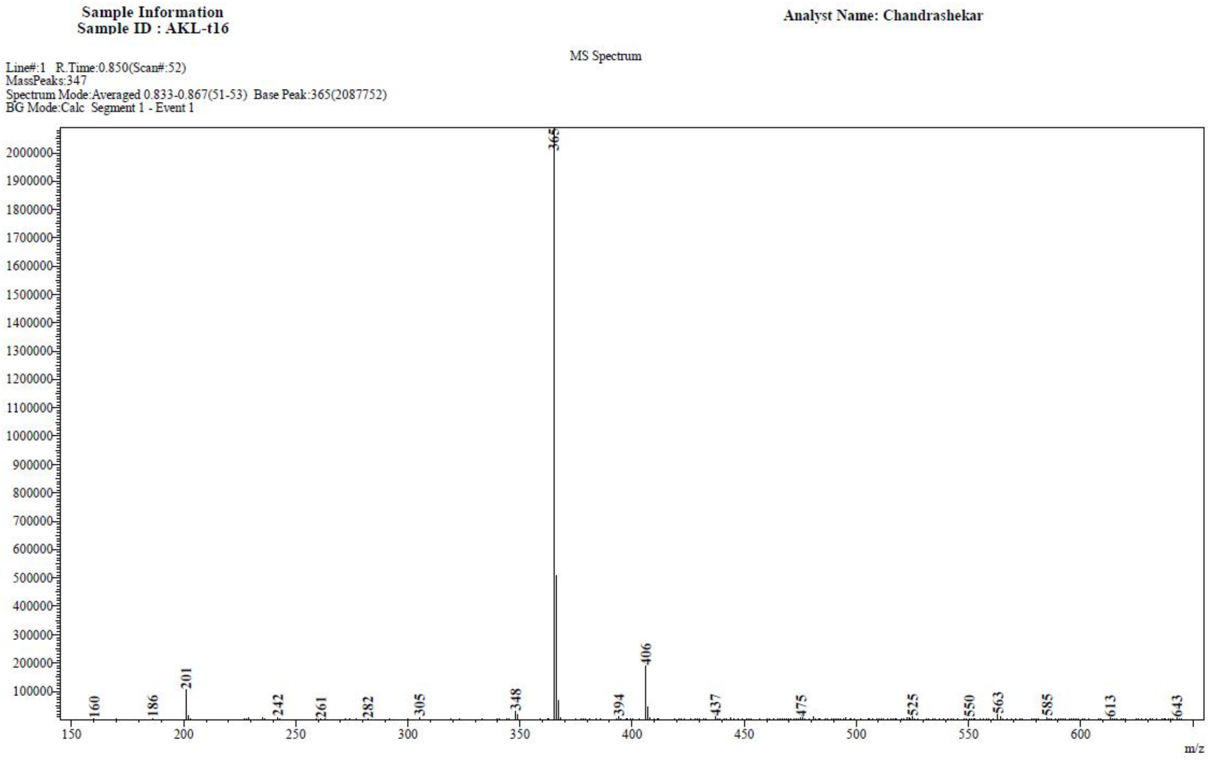

Compound **6i**

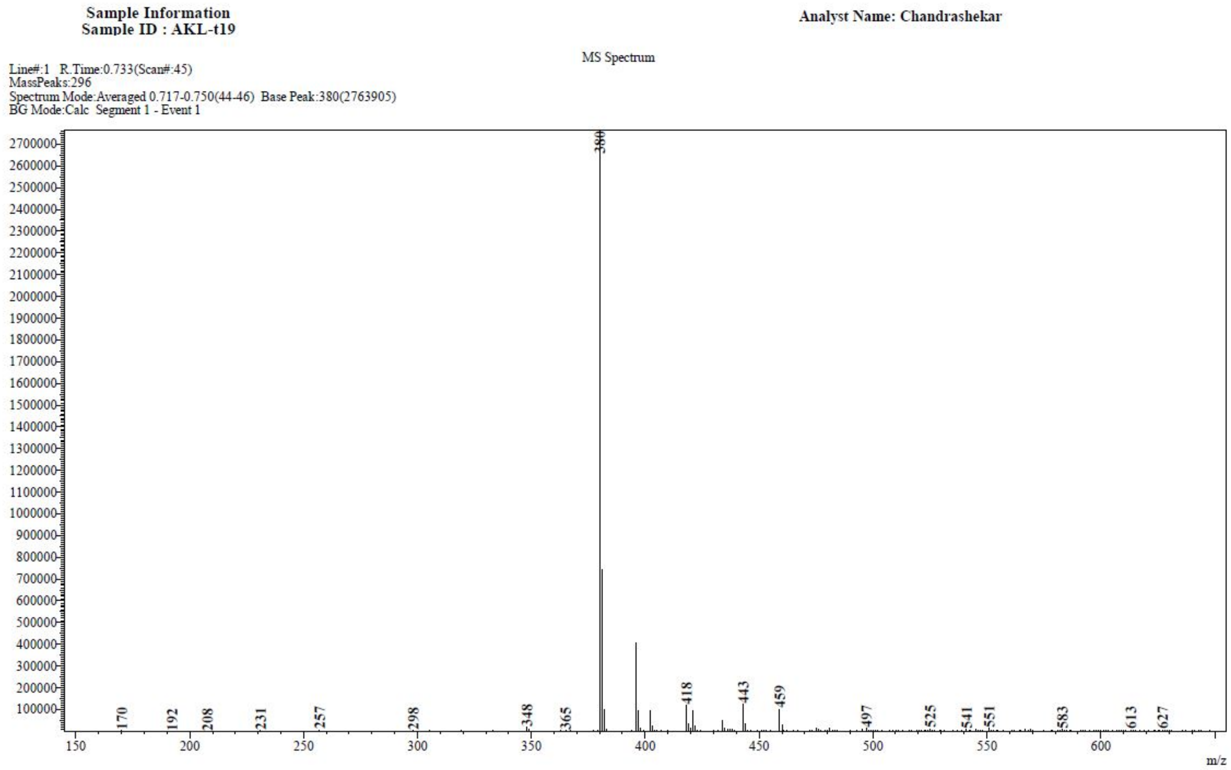

Compound **6j**

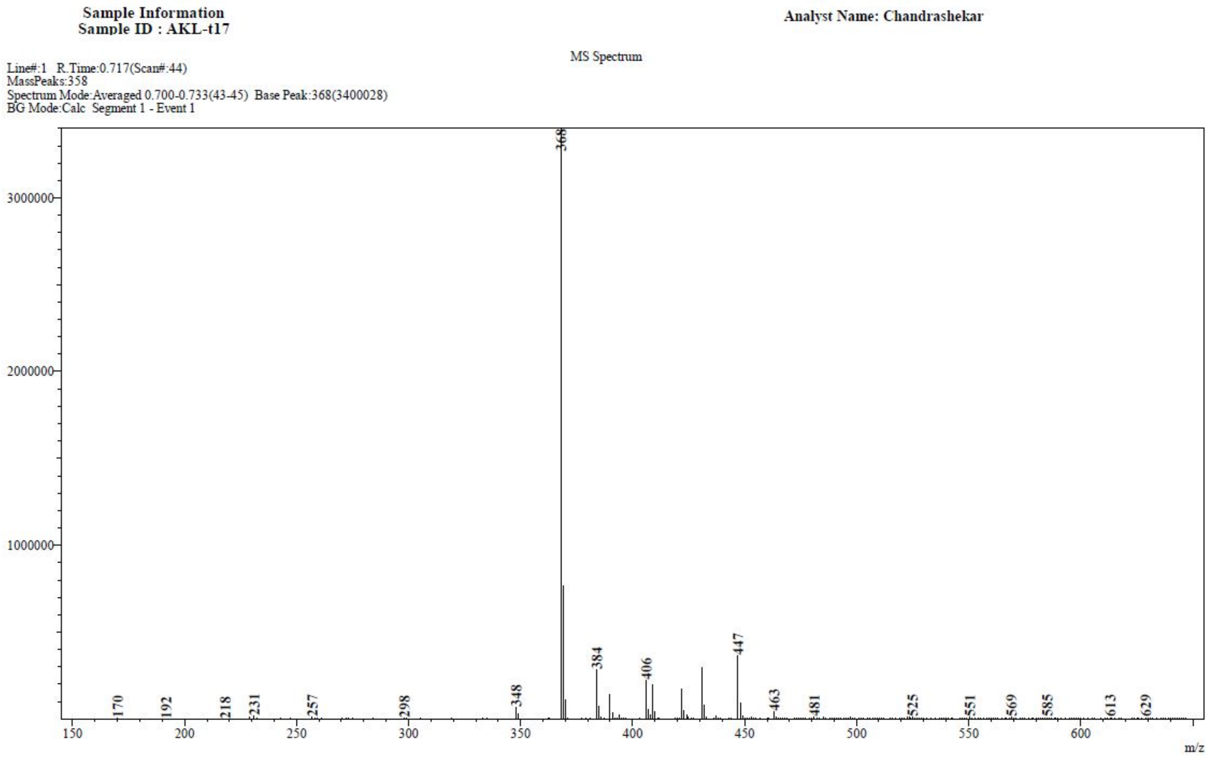

Compound **6k**

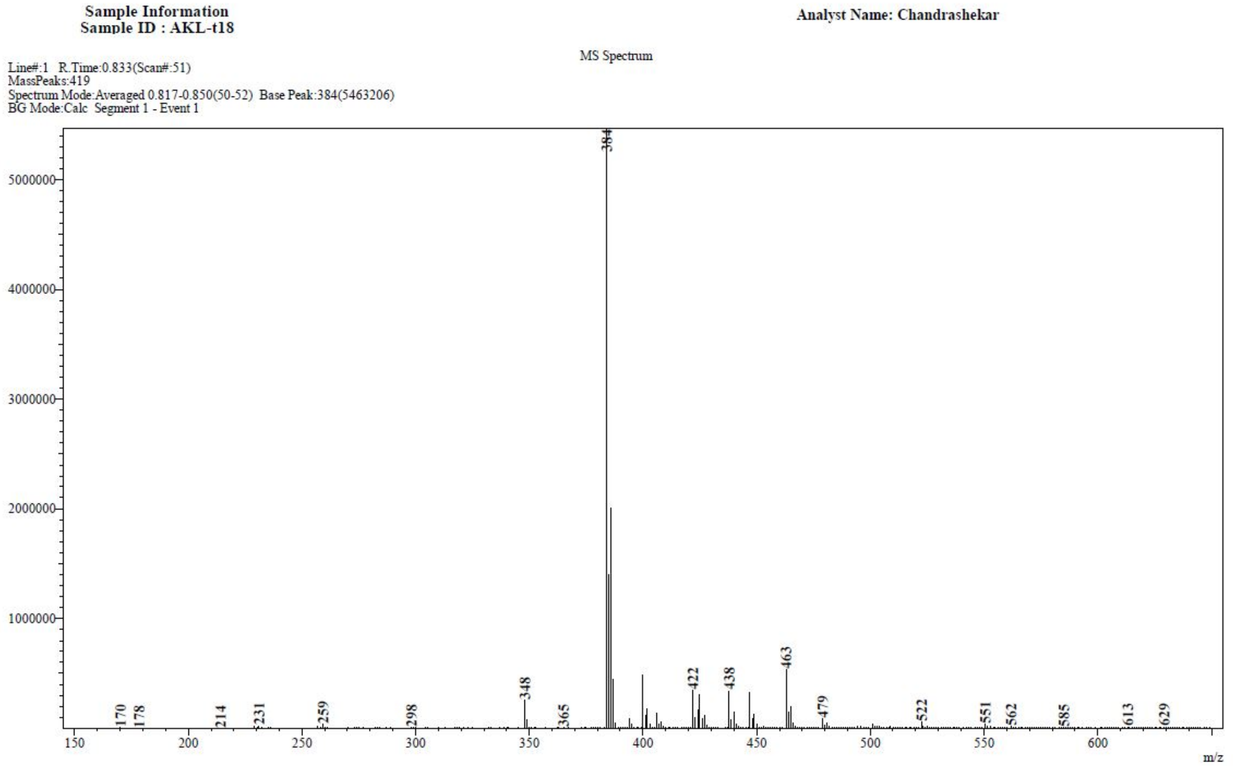

Compound **6l**

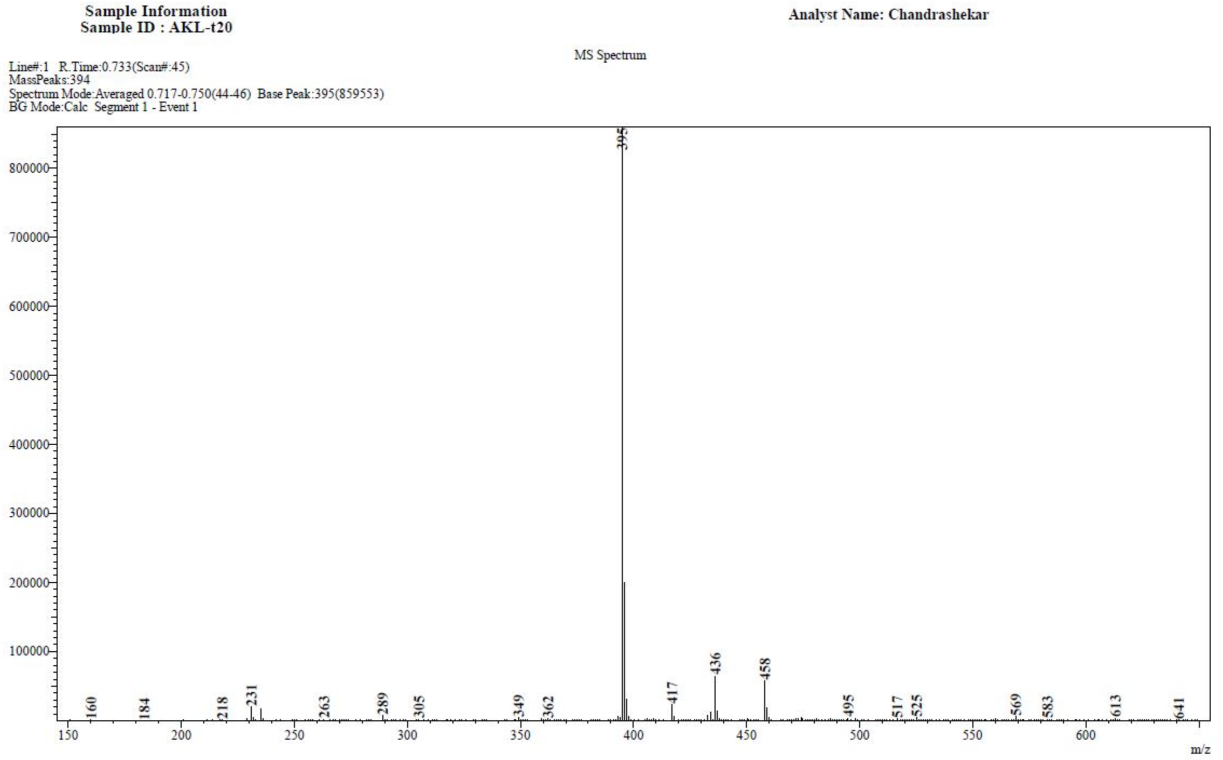

Compound **6m**

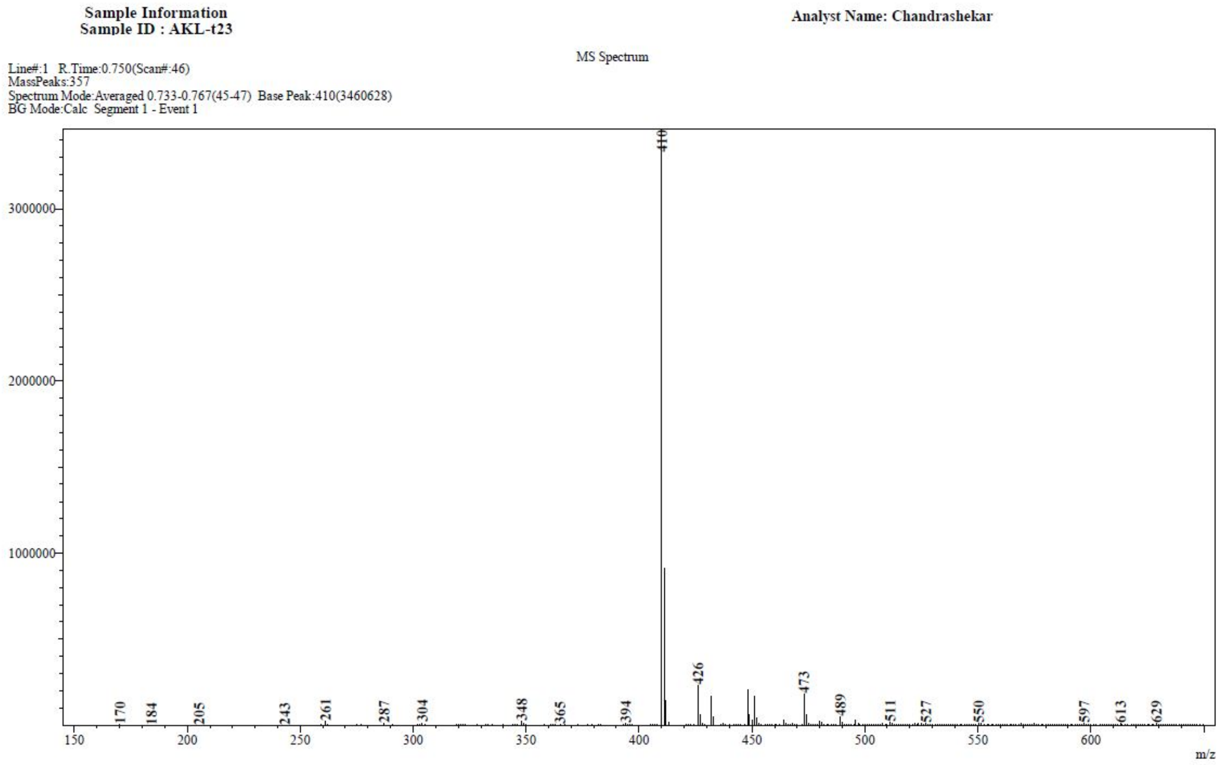

Compound **6n**

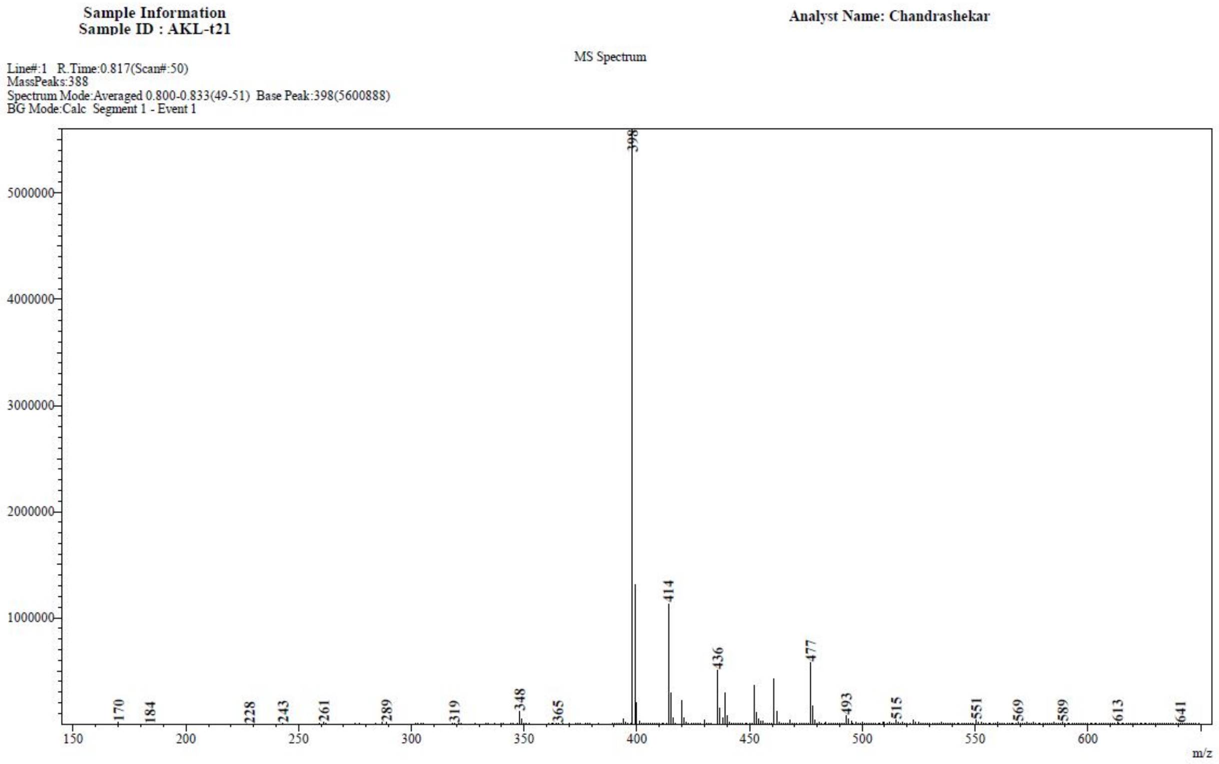

Compound **6o**

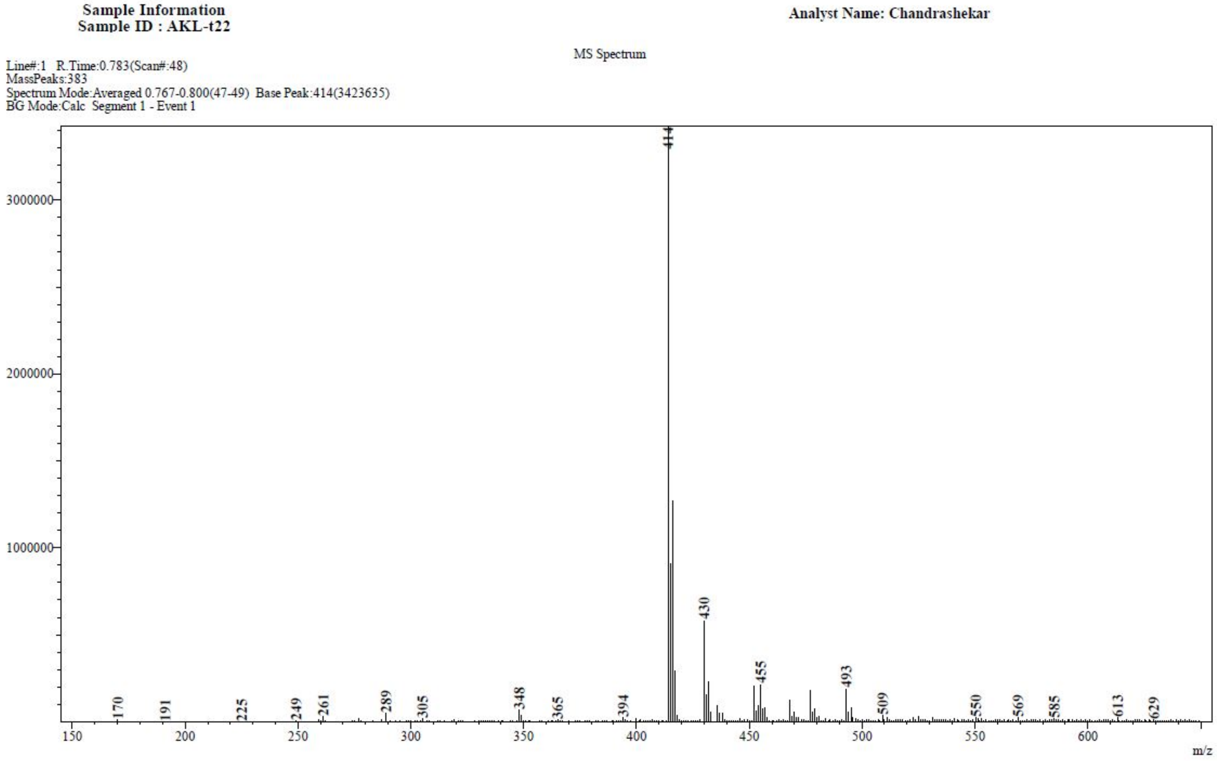

Compound **6p**

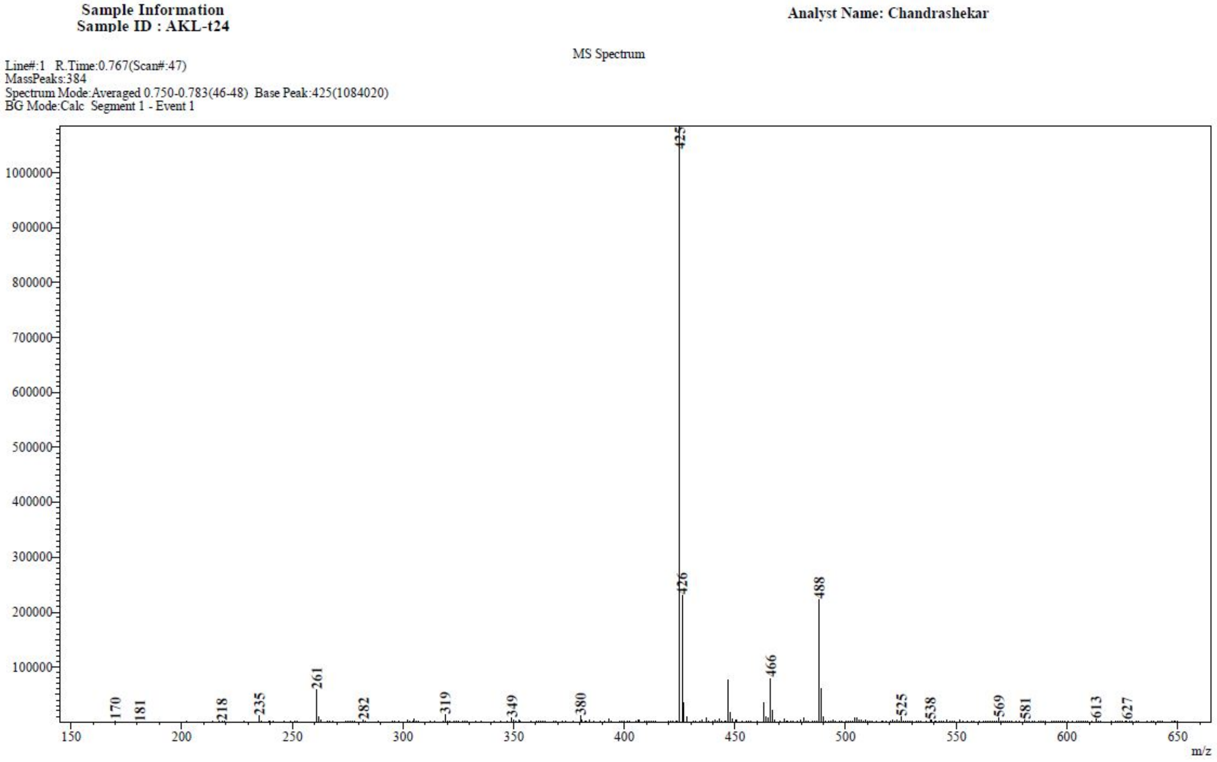

Compound **6q**

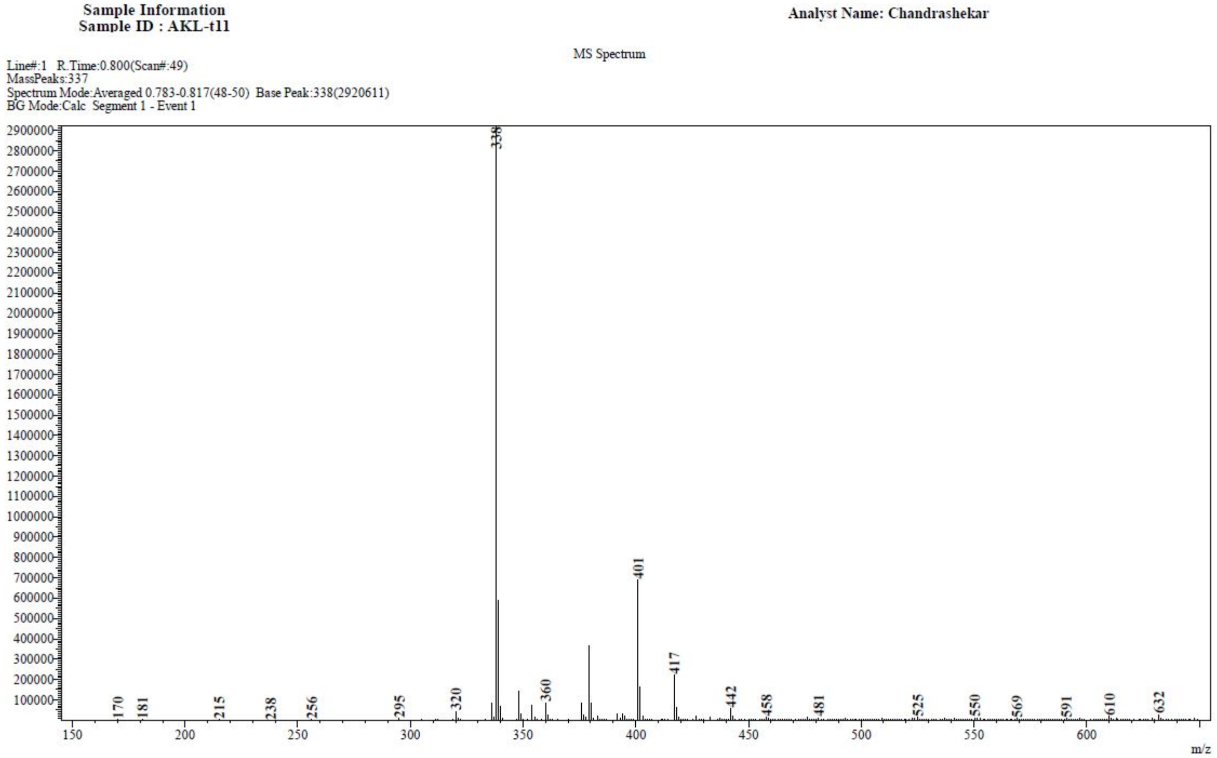

Compound **6r**

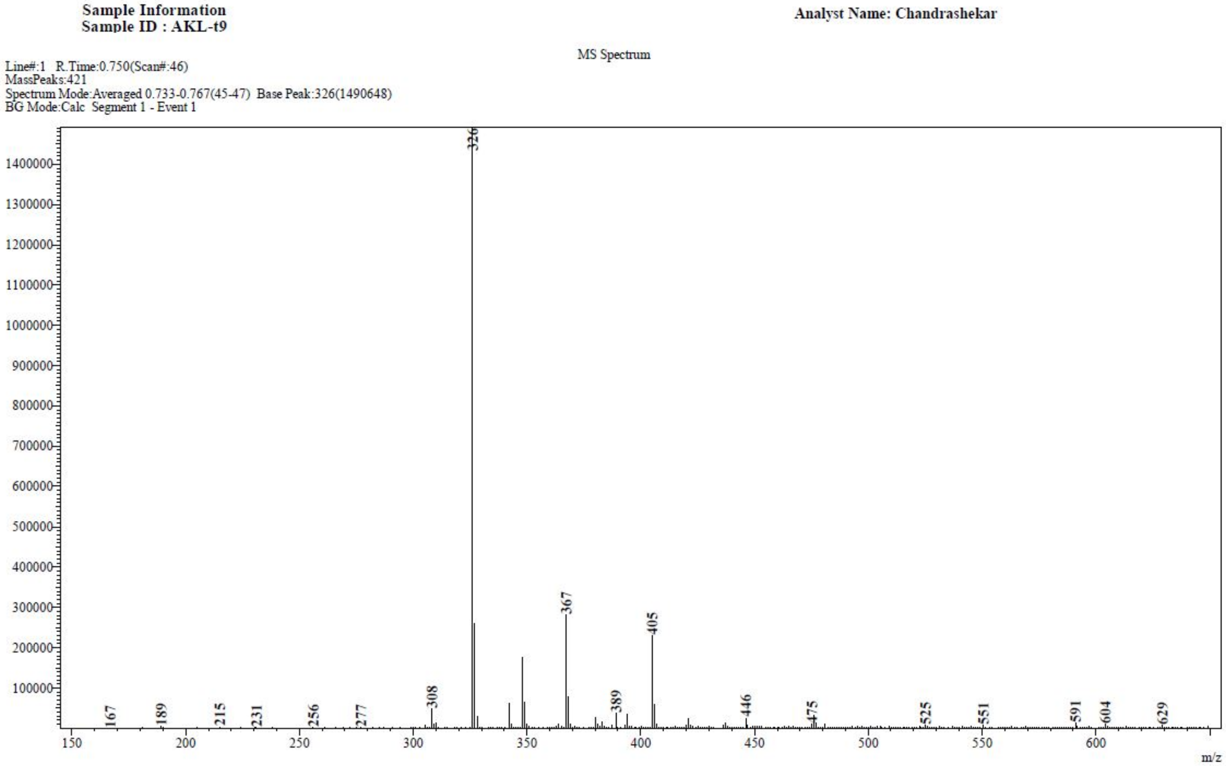

Compound **6s**

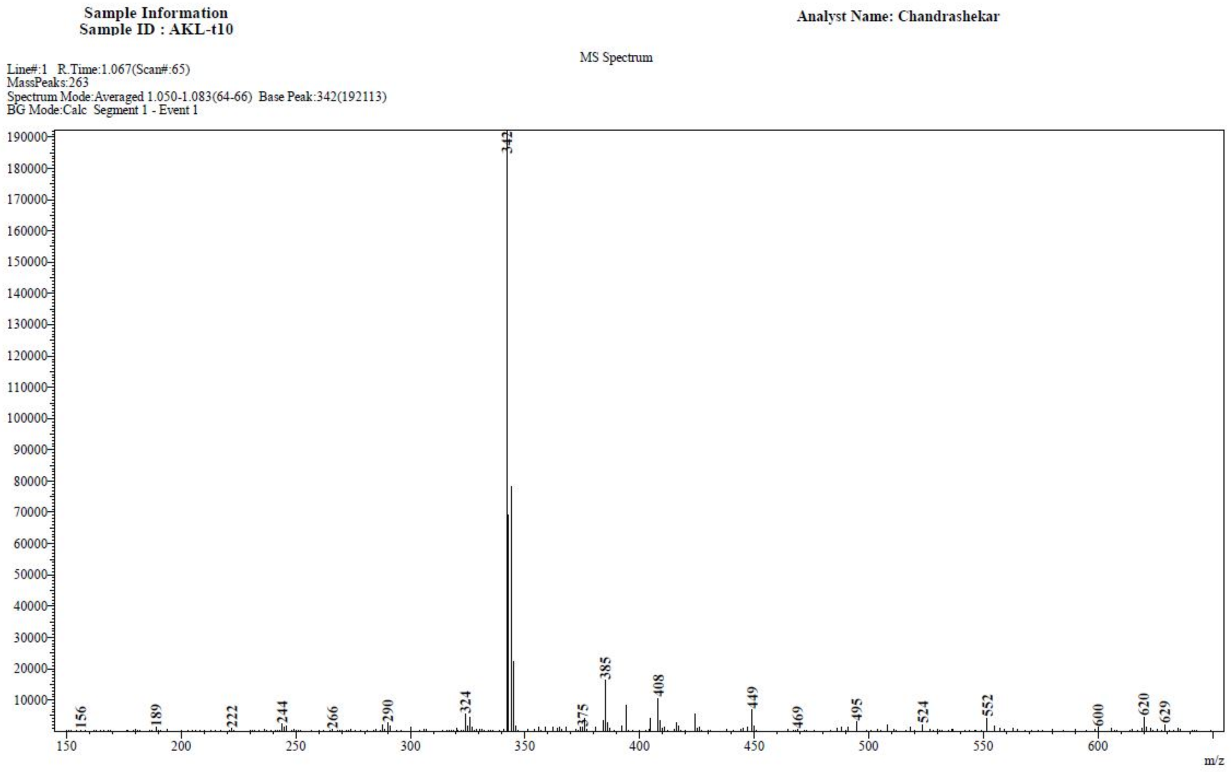

Compound **6t**

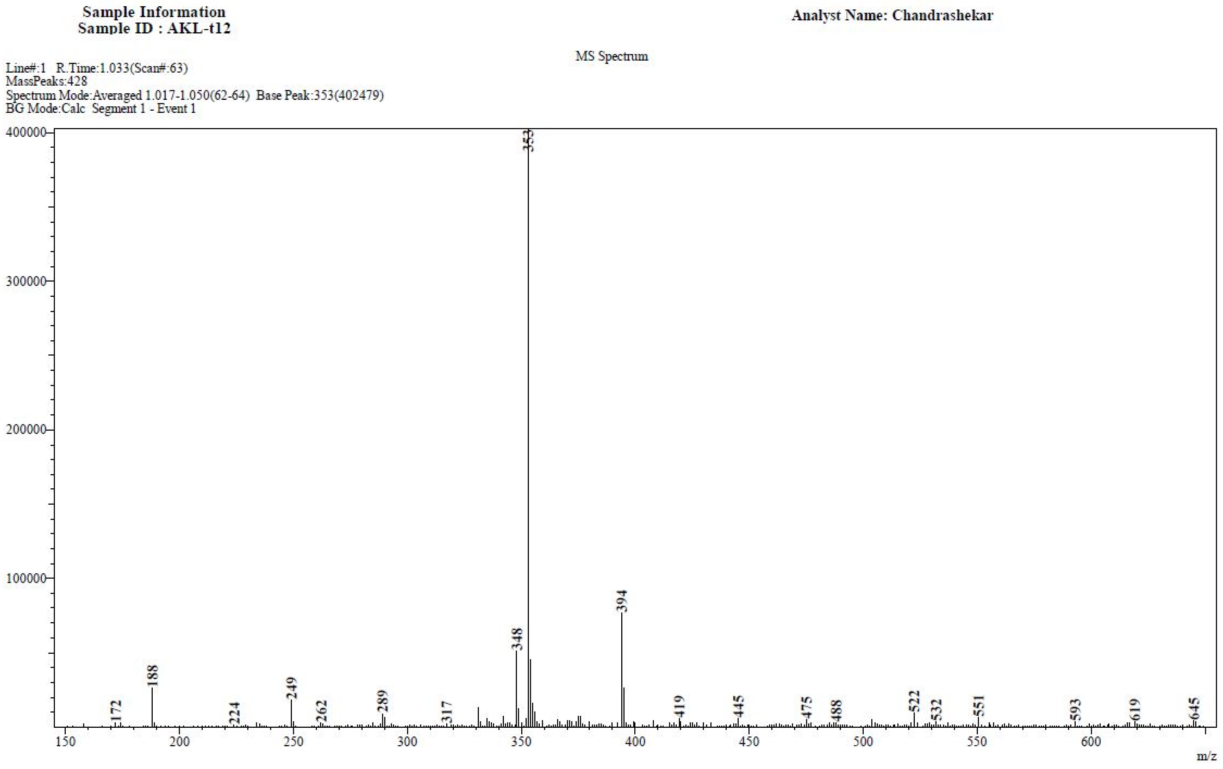

Compound **6u**

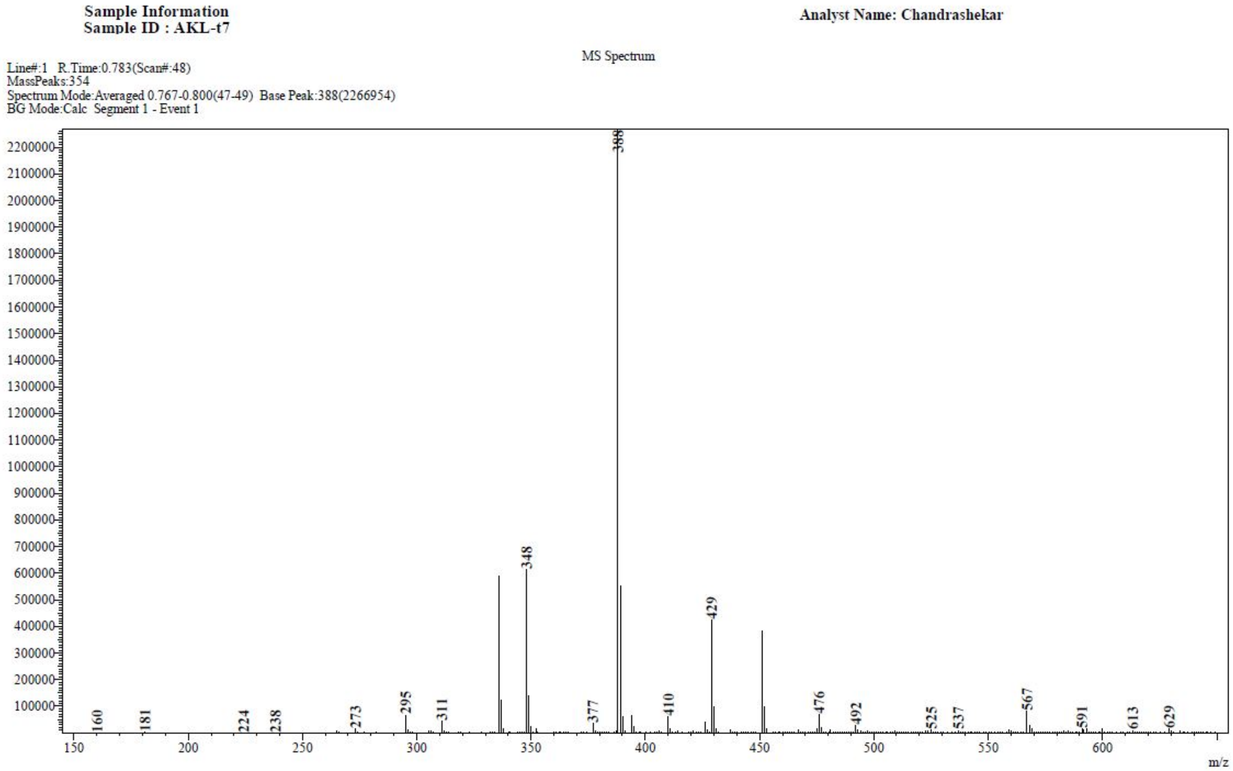

Compound **6v**

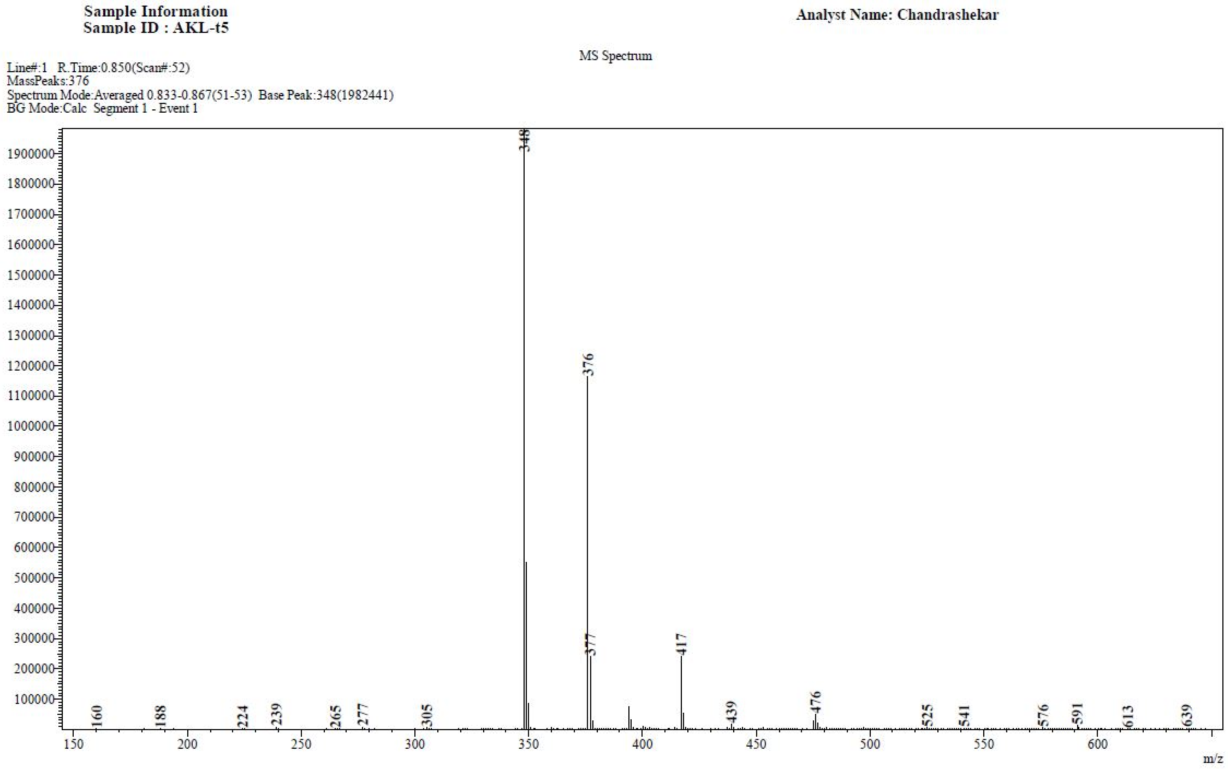

Compound **6w**

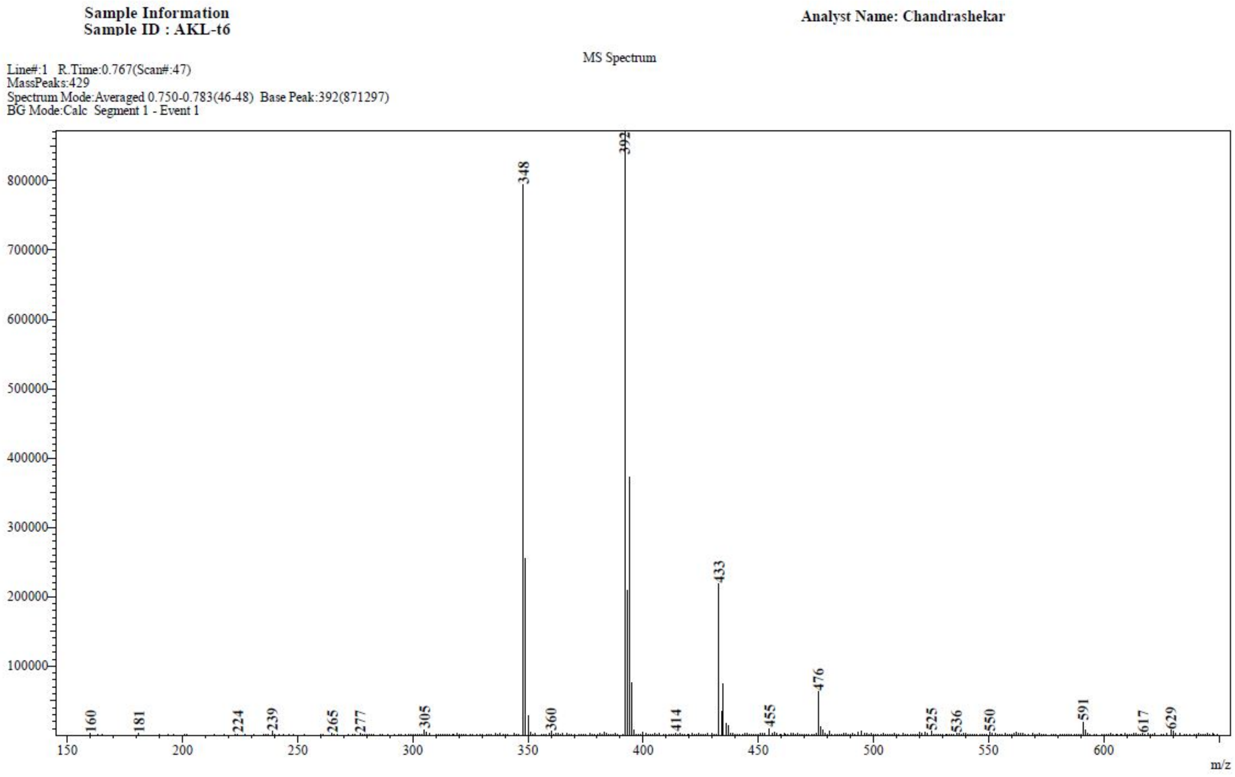

Compound **6x**

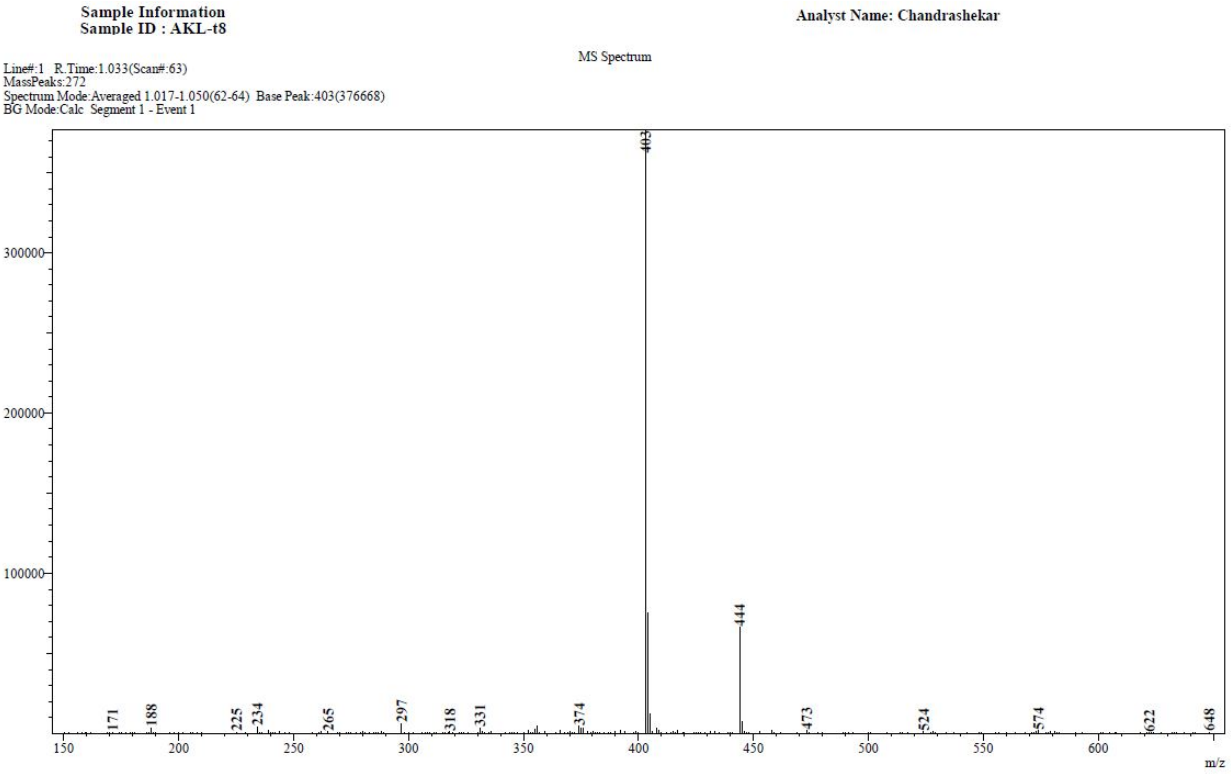

HRMS Reports

Compound **6a**

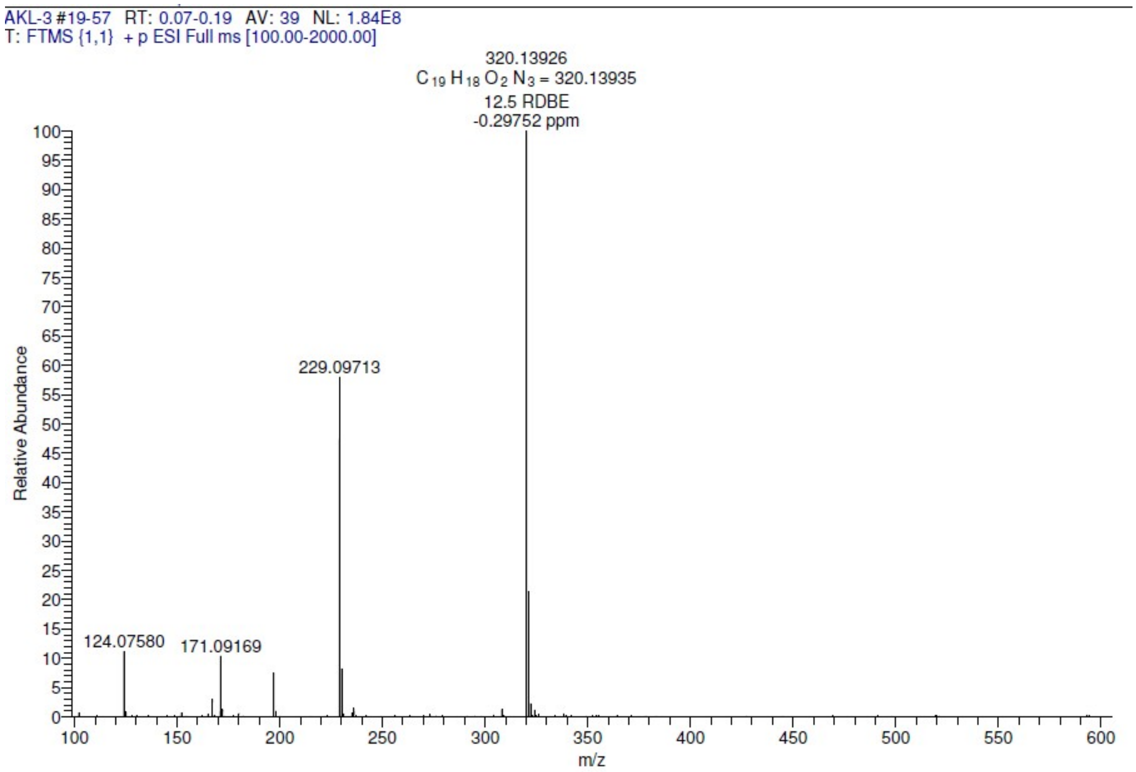

Compound **6b**

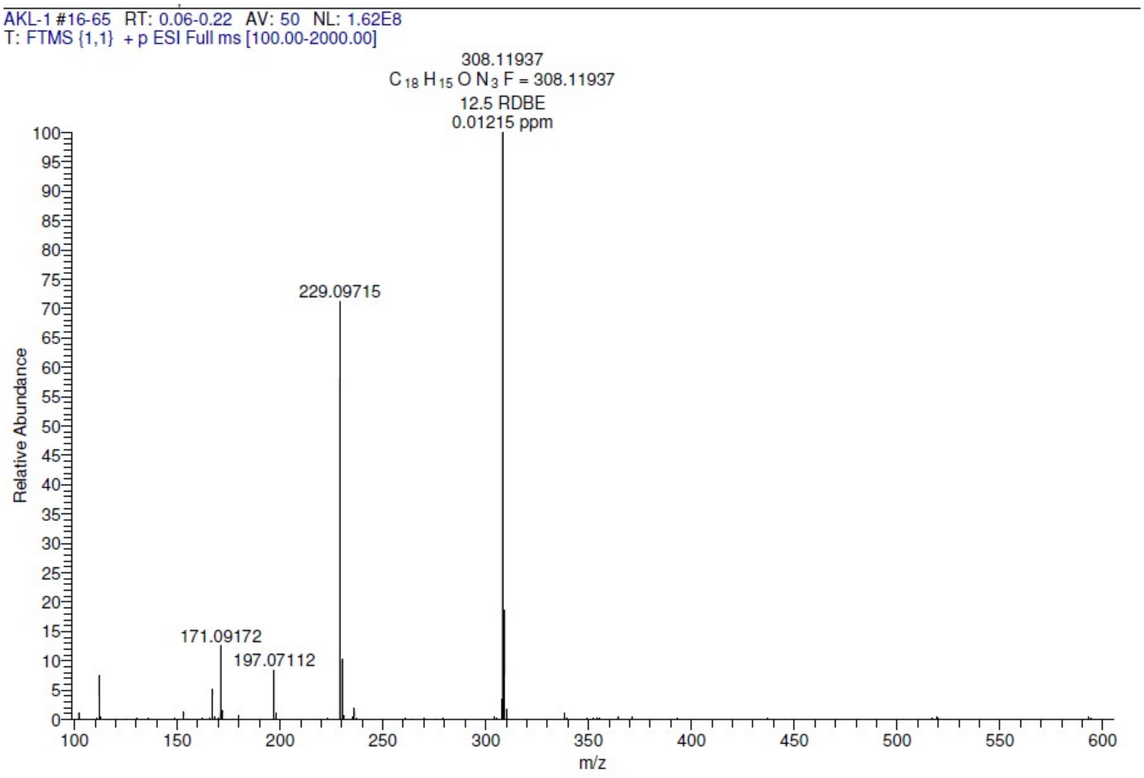

Compound **6c**

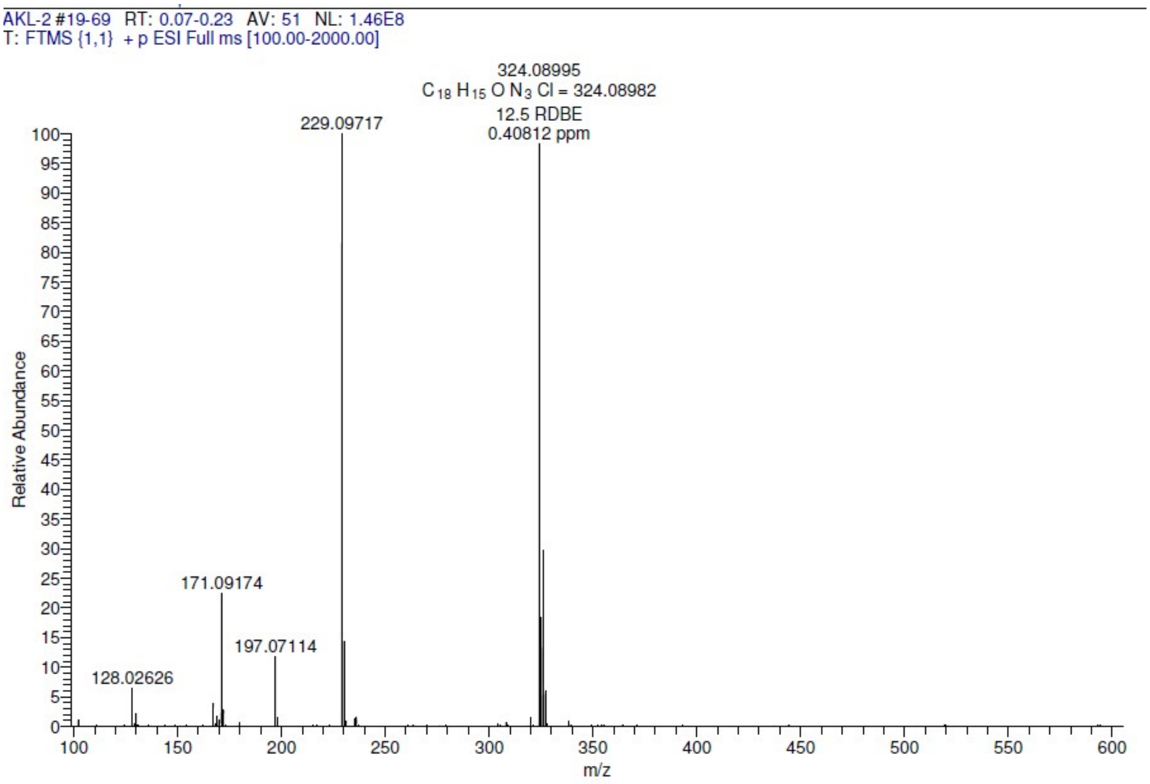

Compound **6d**

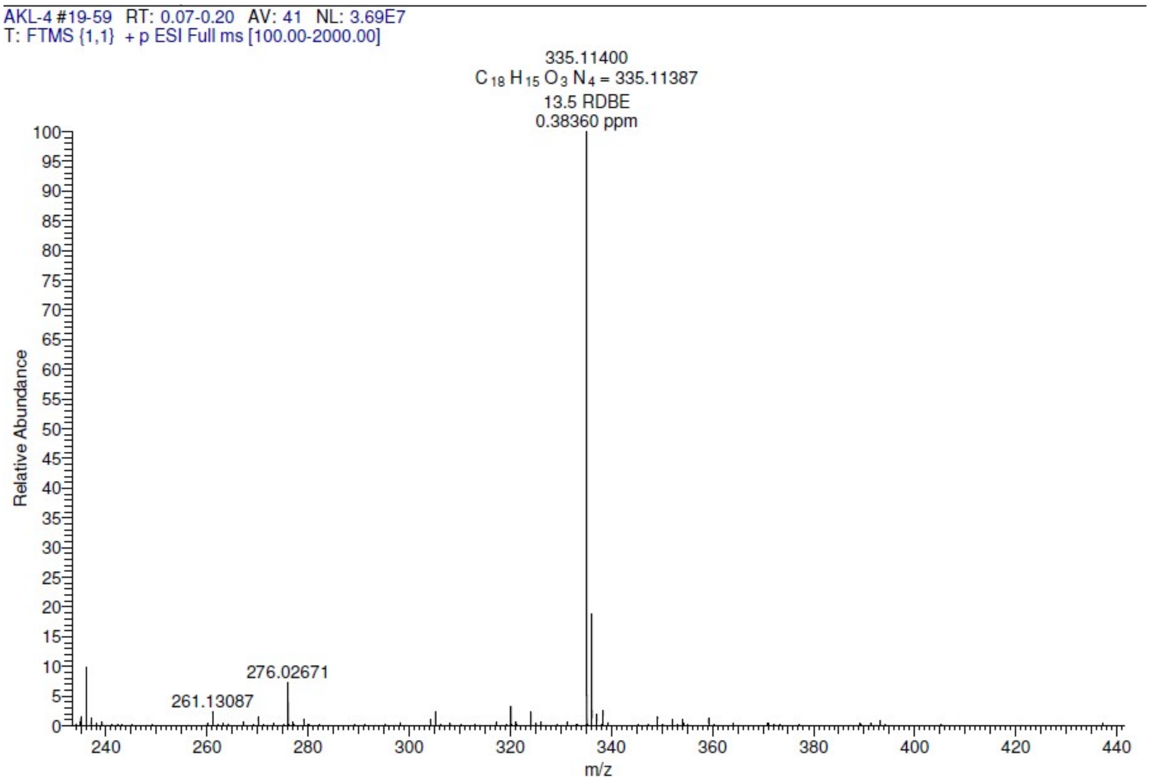

Compound **6e**

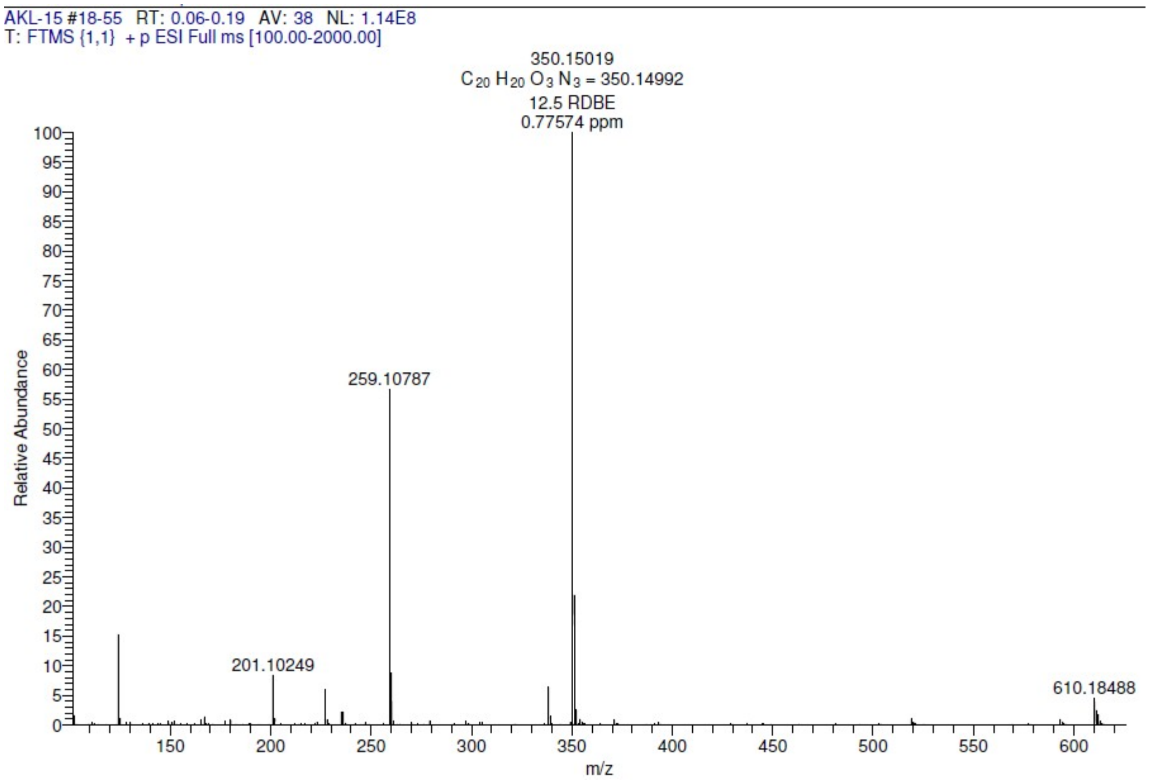

Compound **6f**

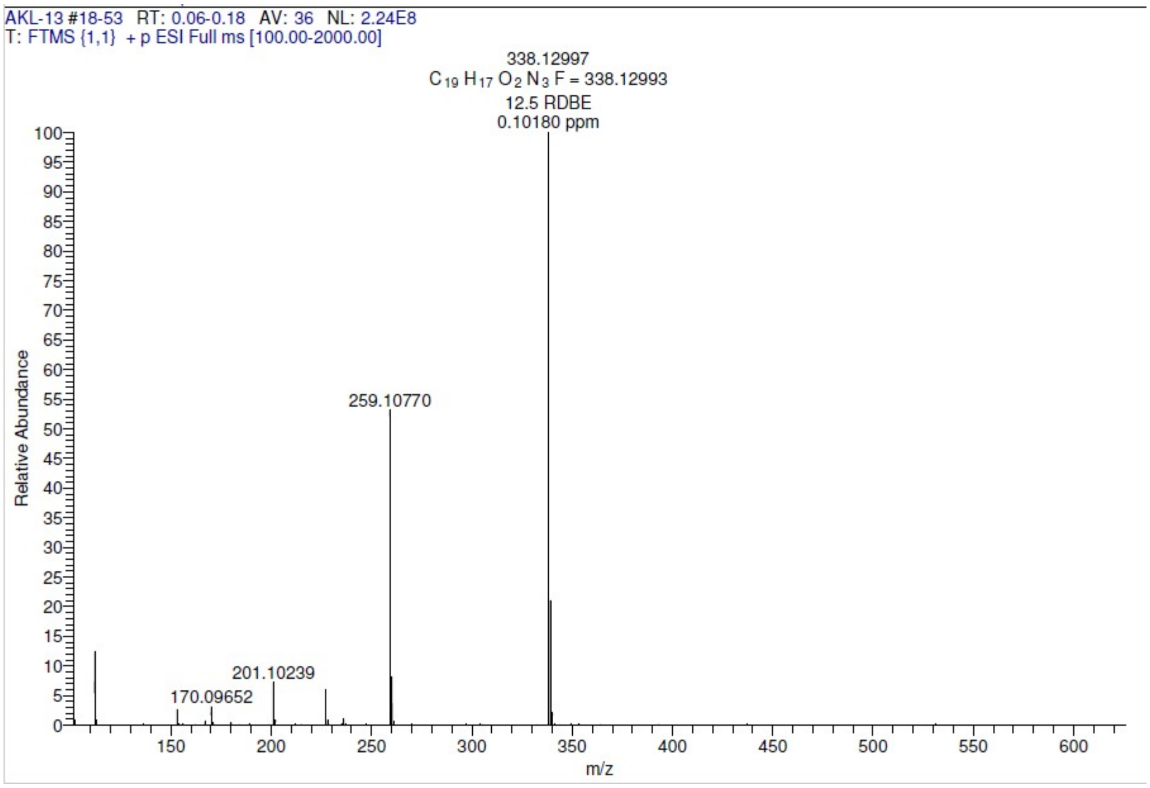

Compound **6g**

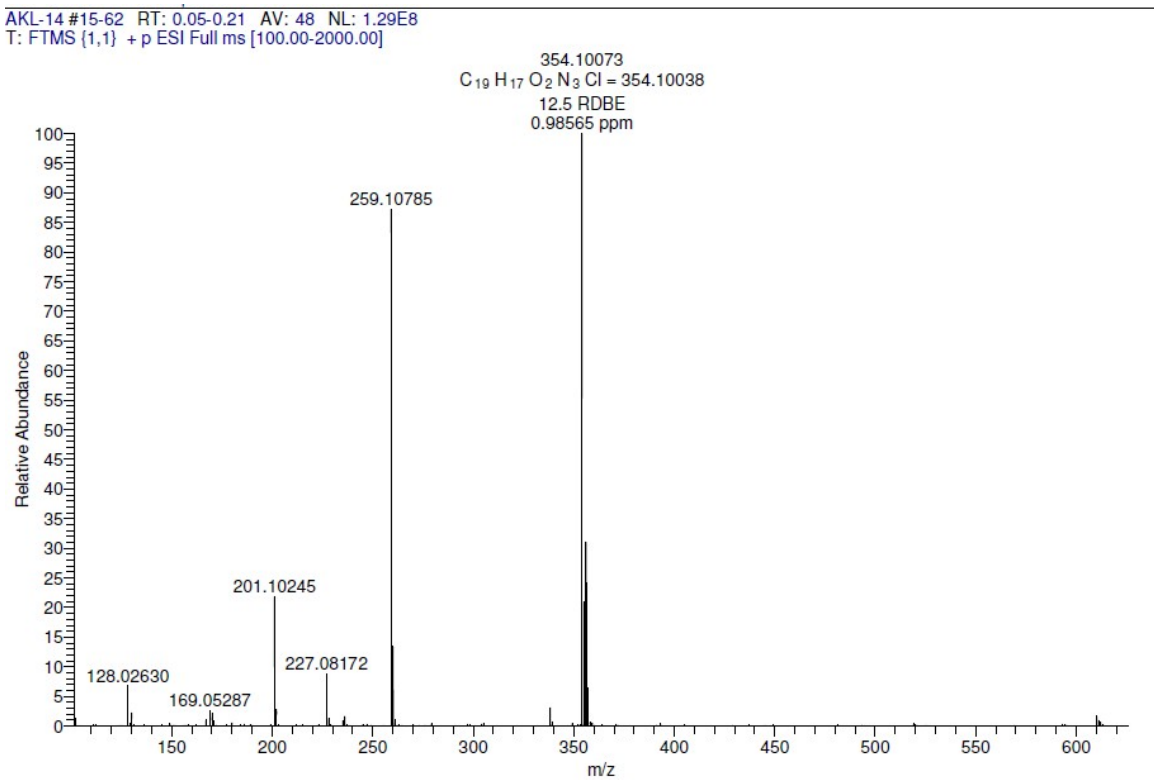

Compound **6h**

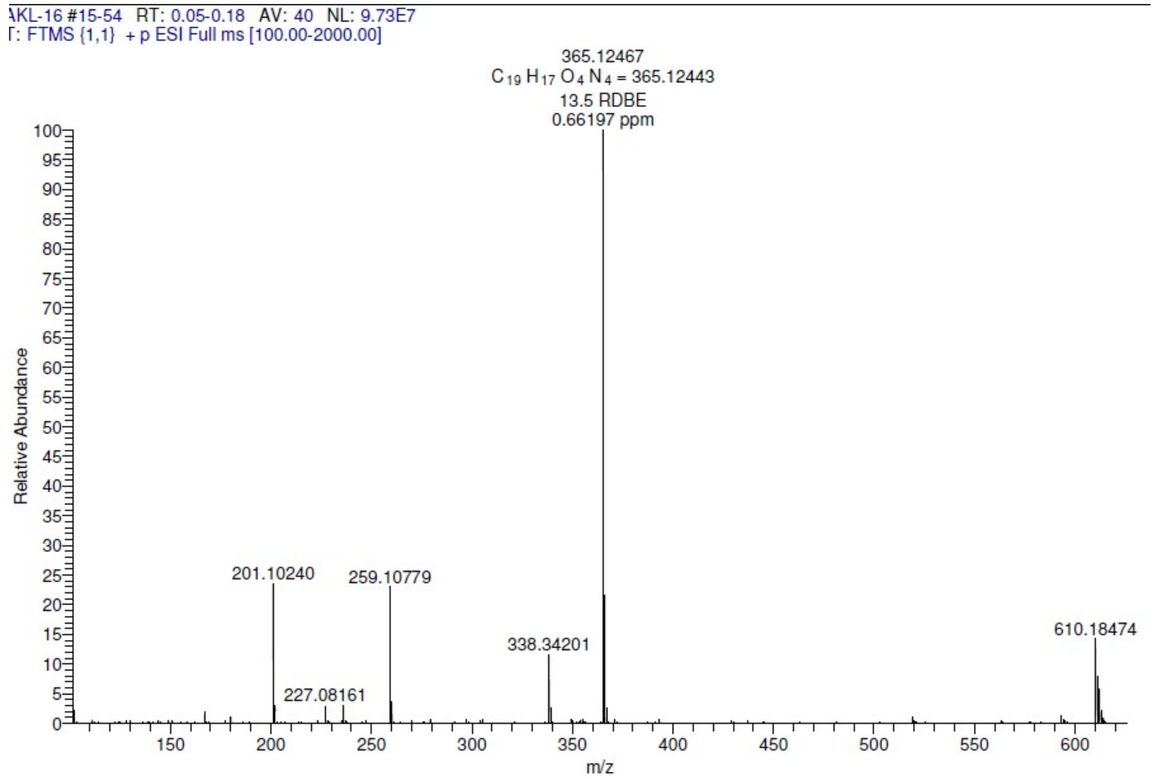

Compound **6i**

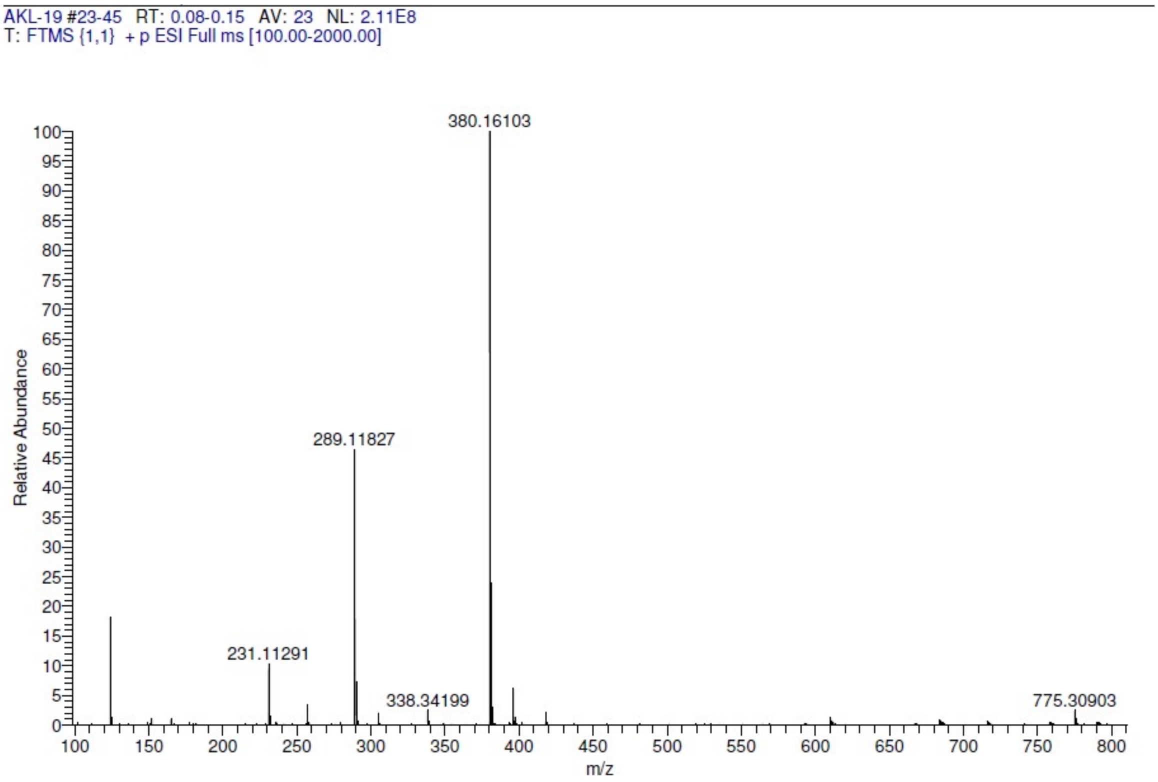

Compound **6j**

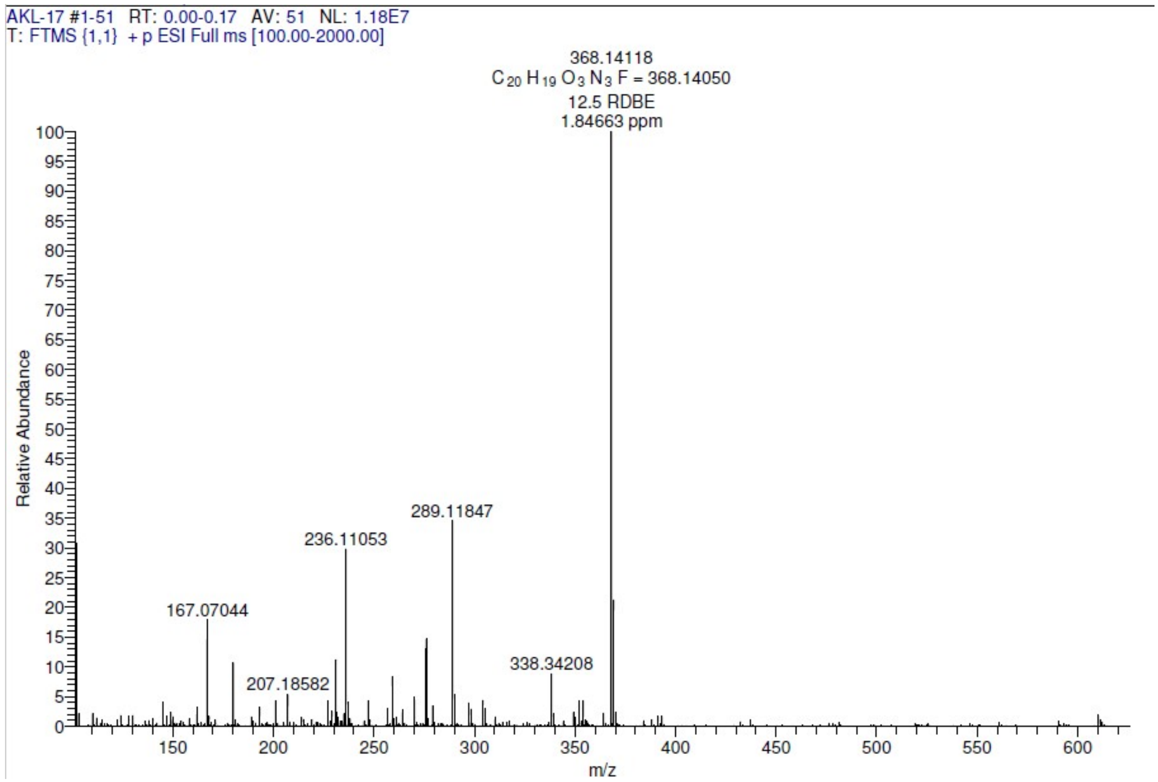

Compound **6k**

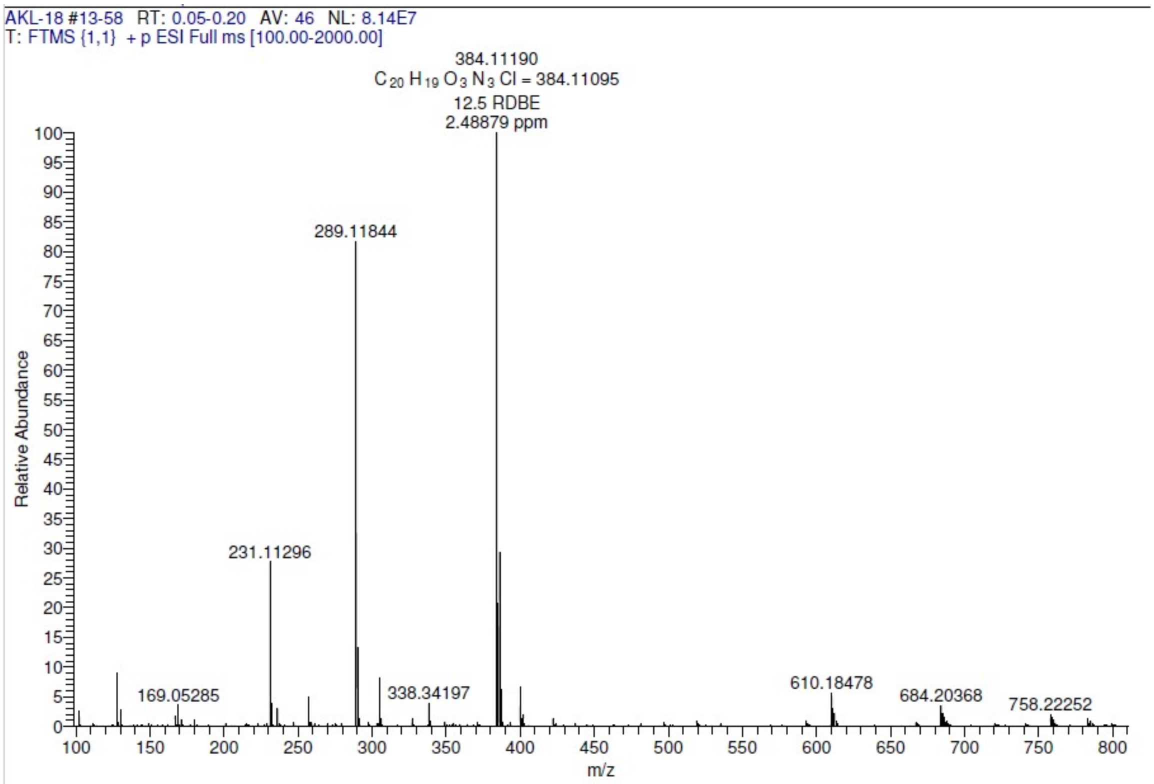

Compound **6l**

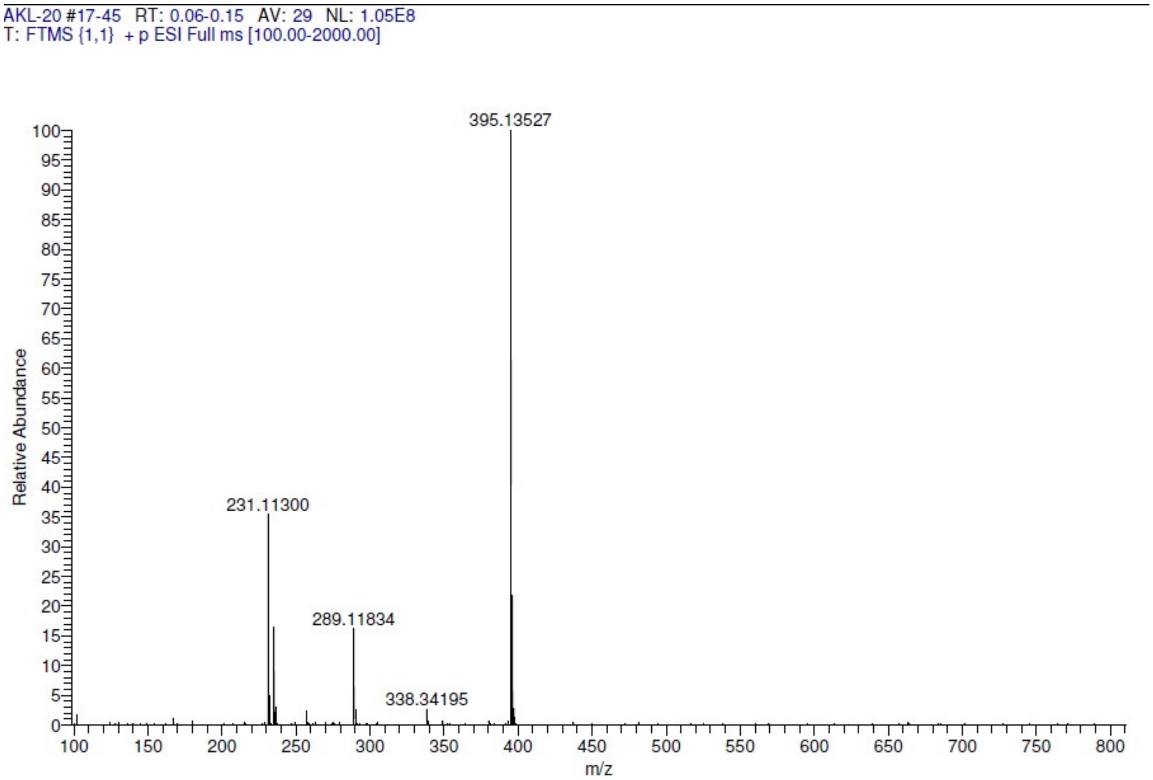

Compound **6m**

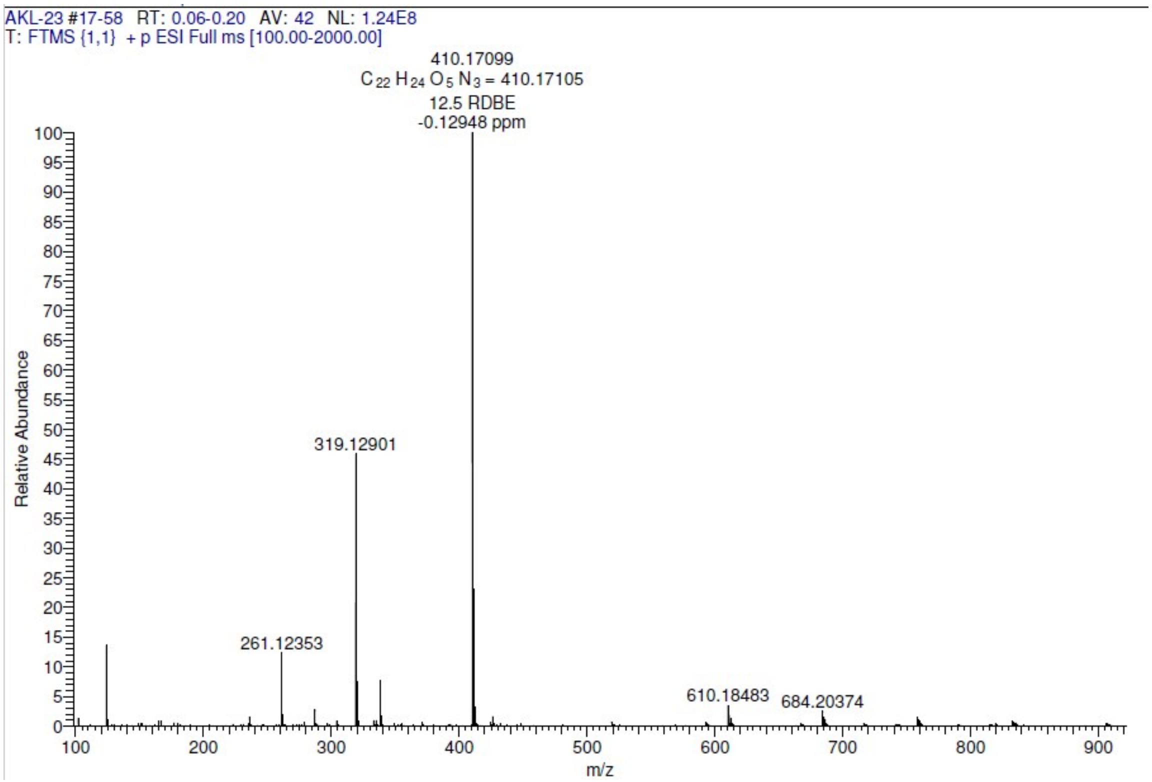

Compound **6n**

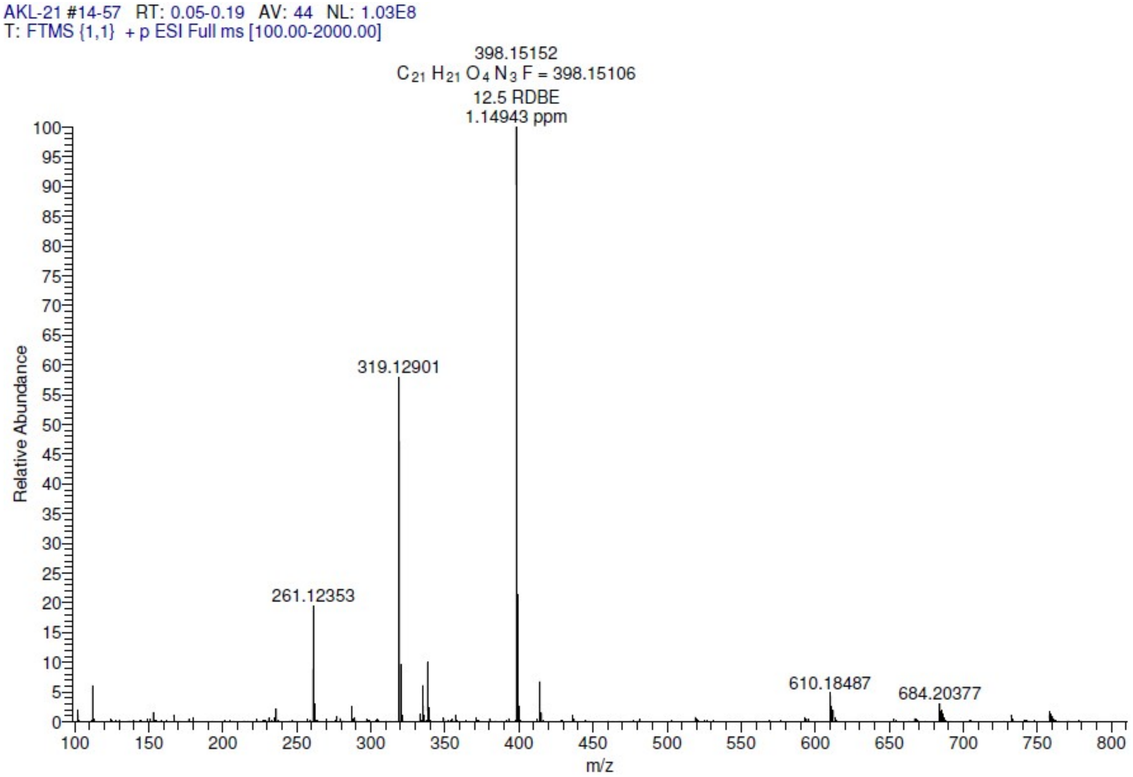

Compound **6o**

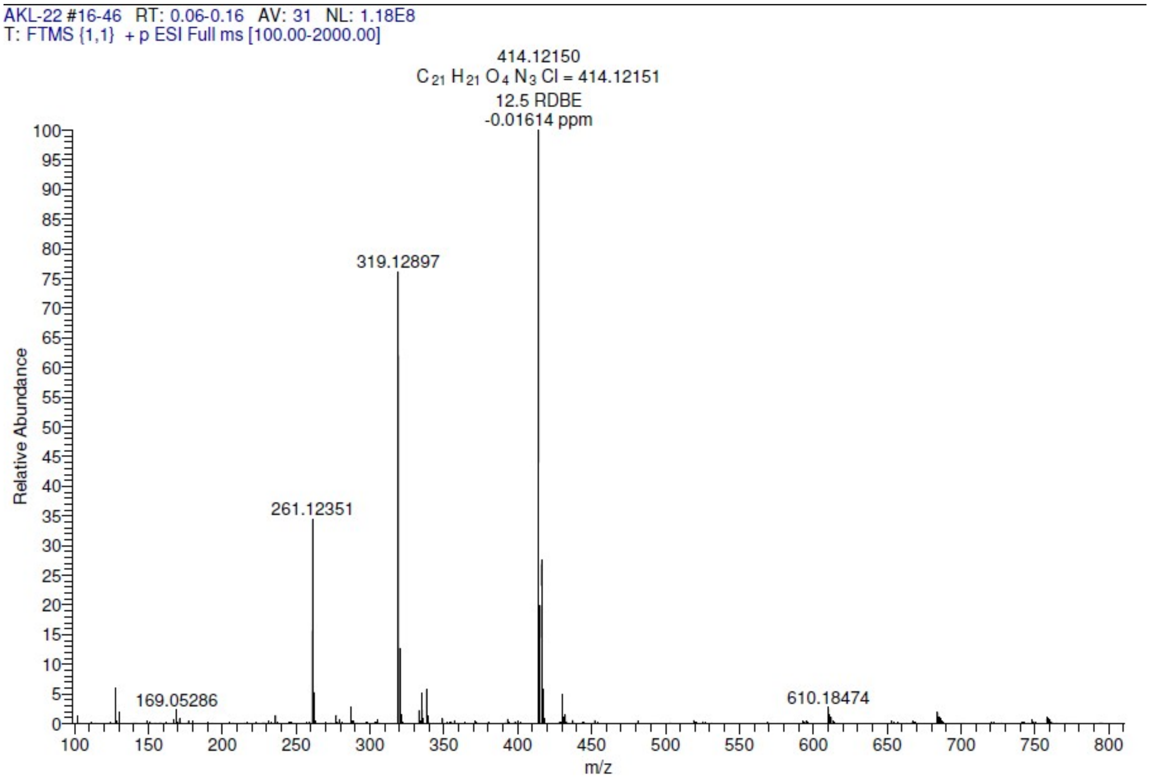

Compound **6p**

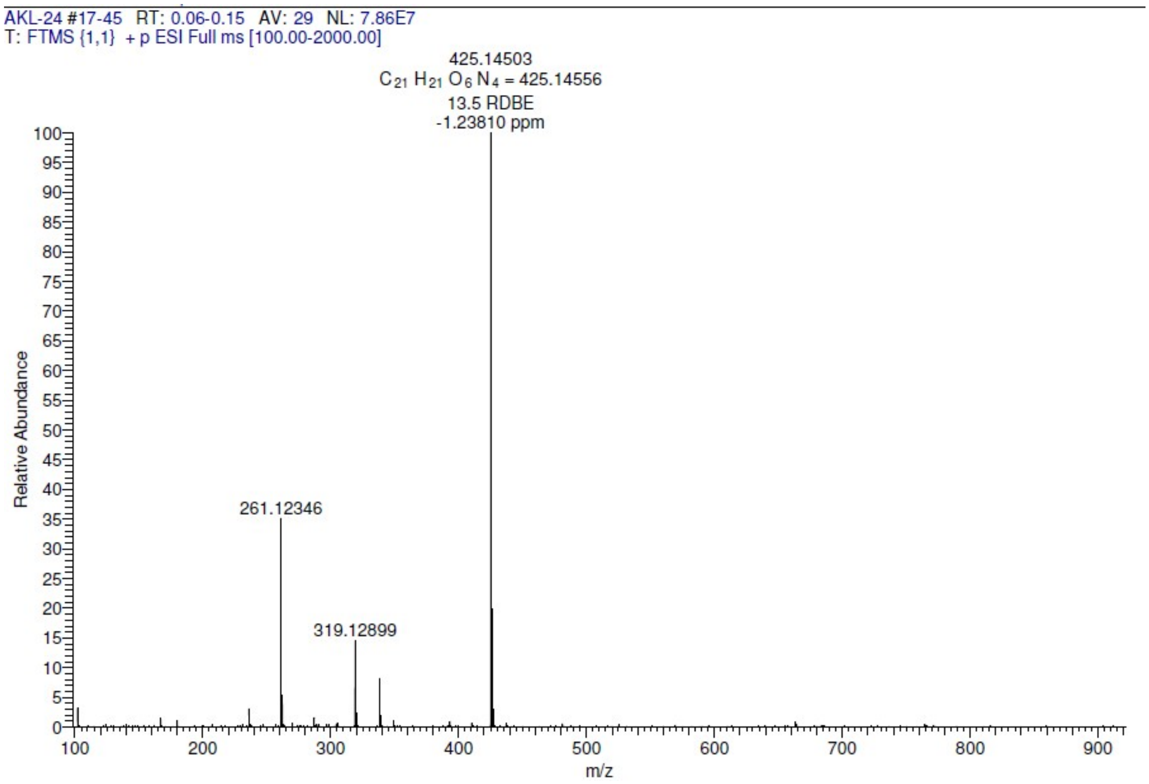

Compound **6q**

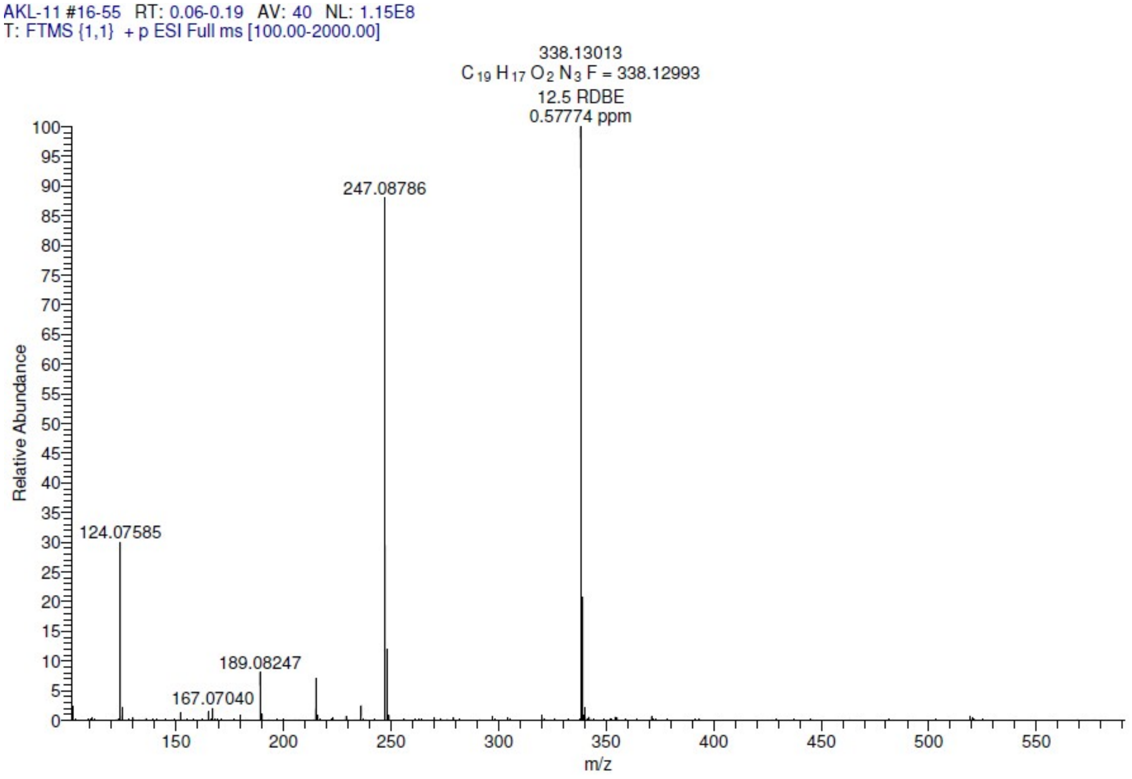

Compound **6r**

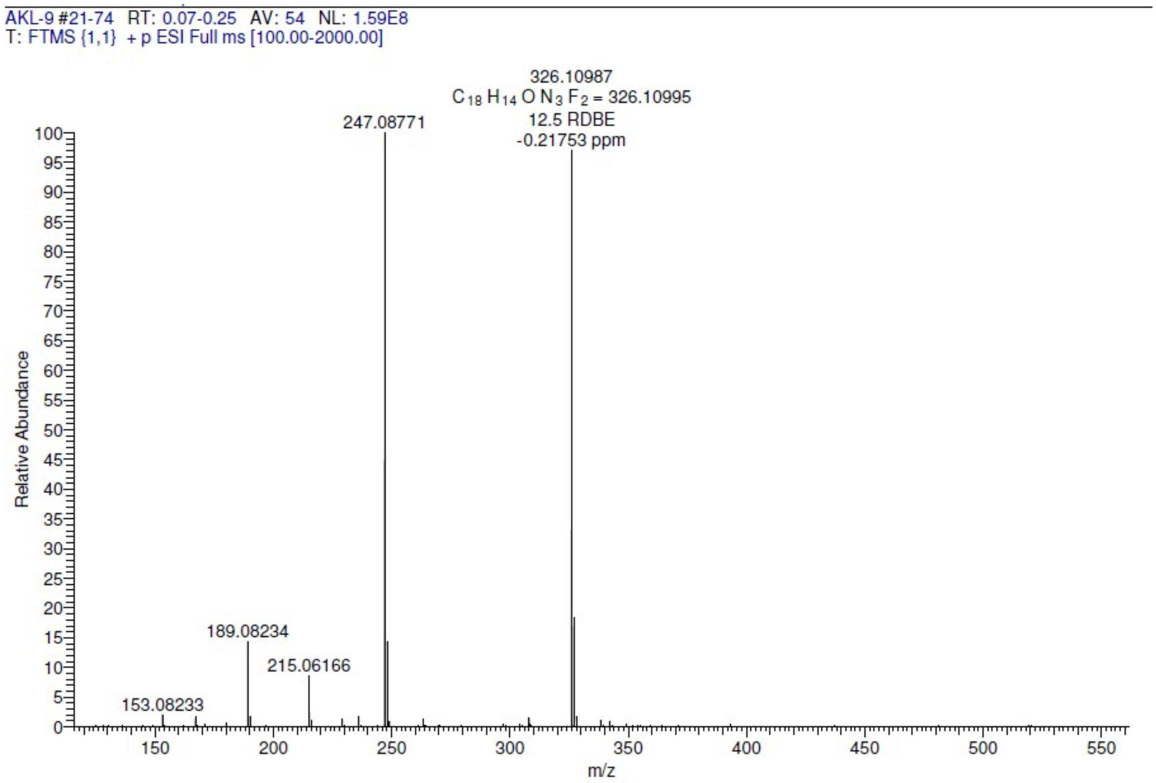

Compound **6s**

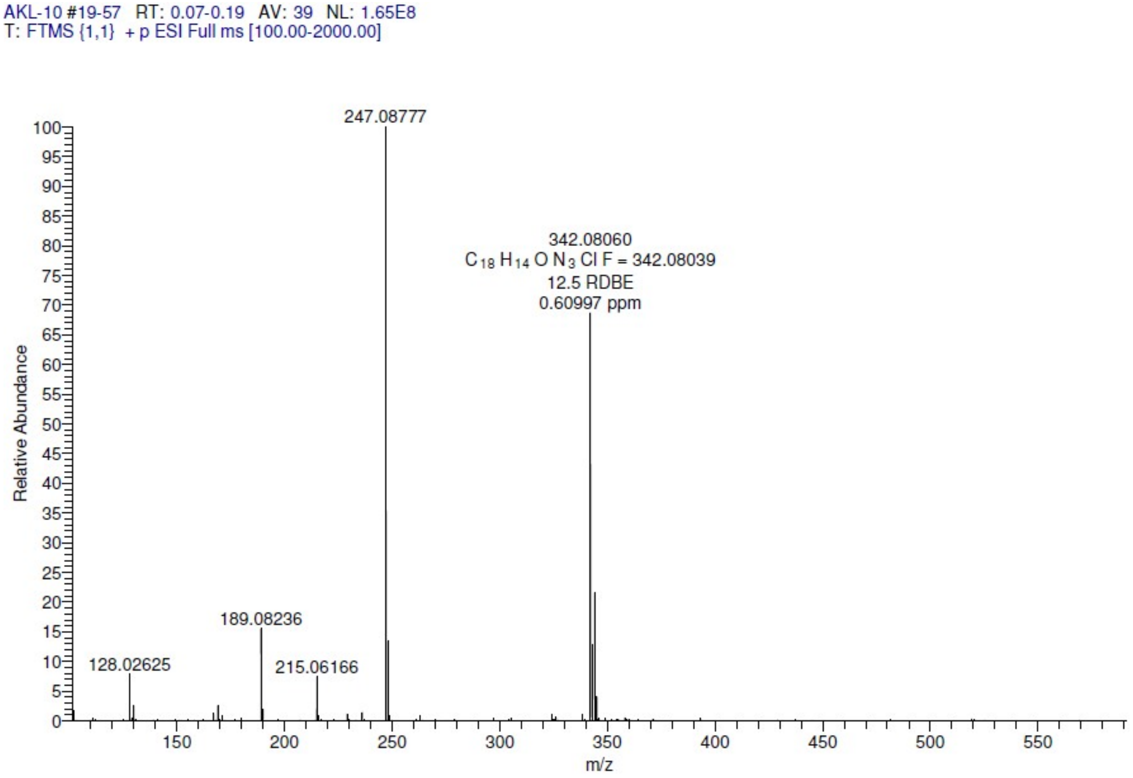

Compound **6t**

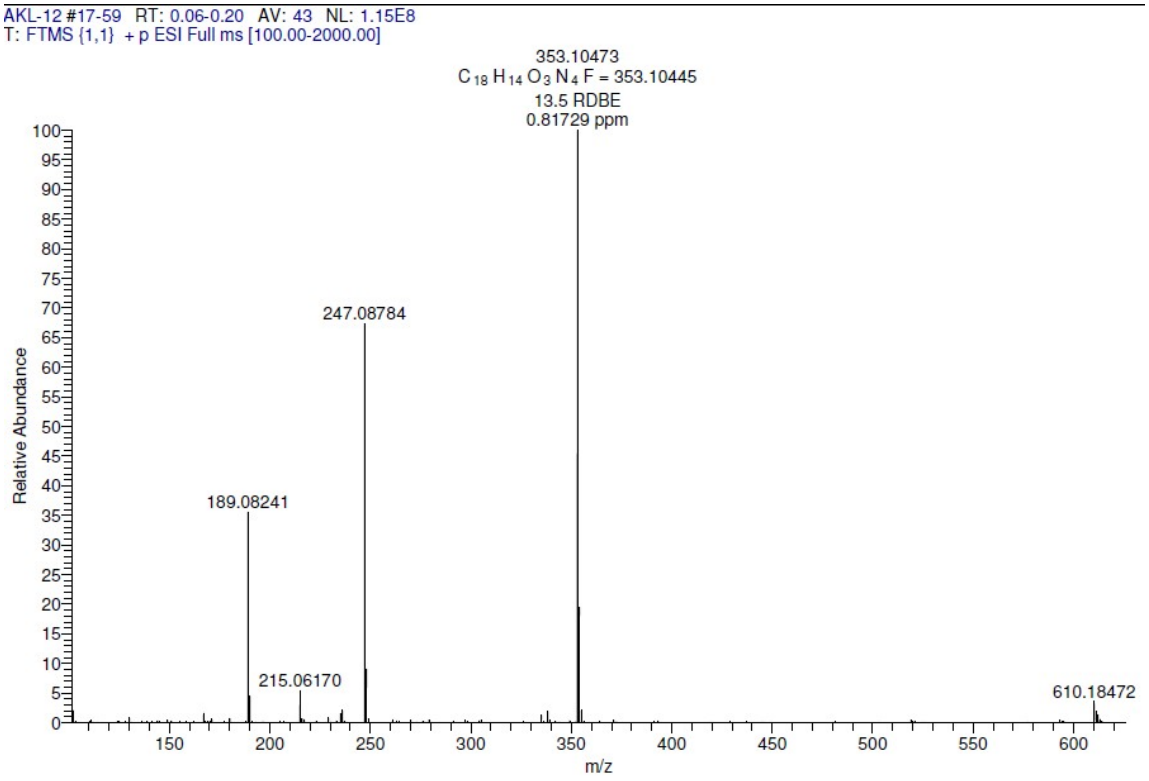

Compound **6u**

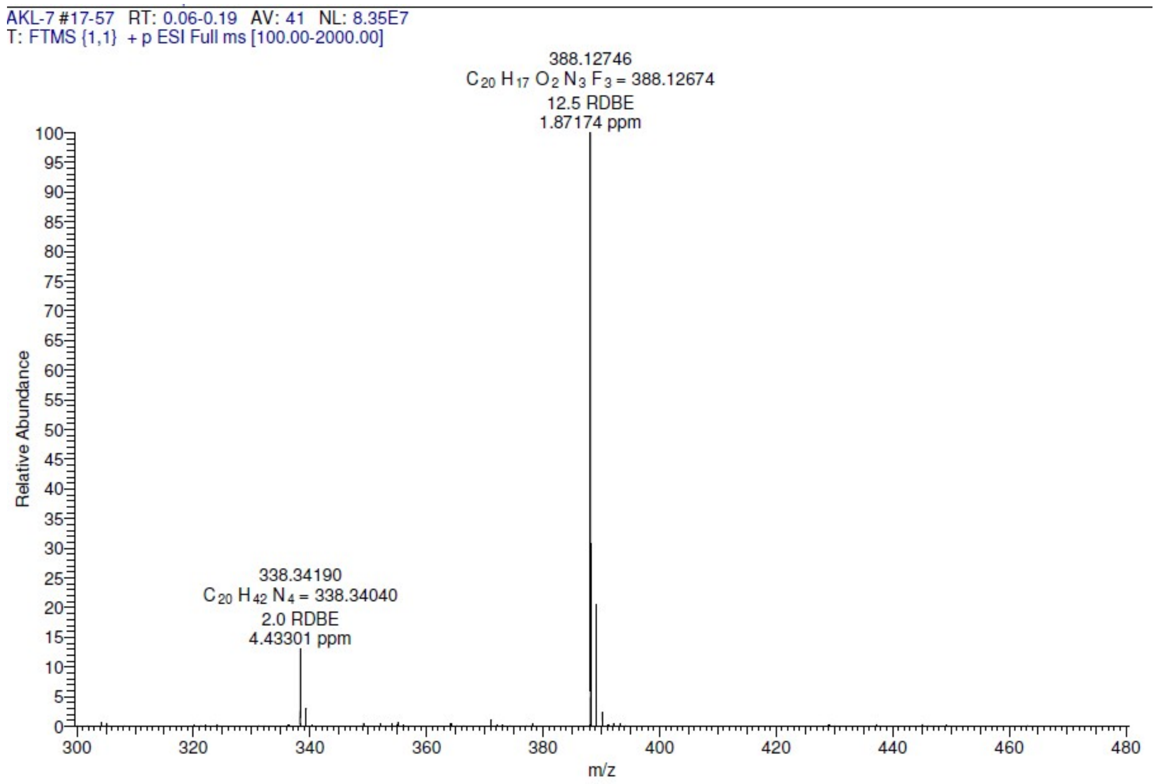

Compound **6v**

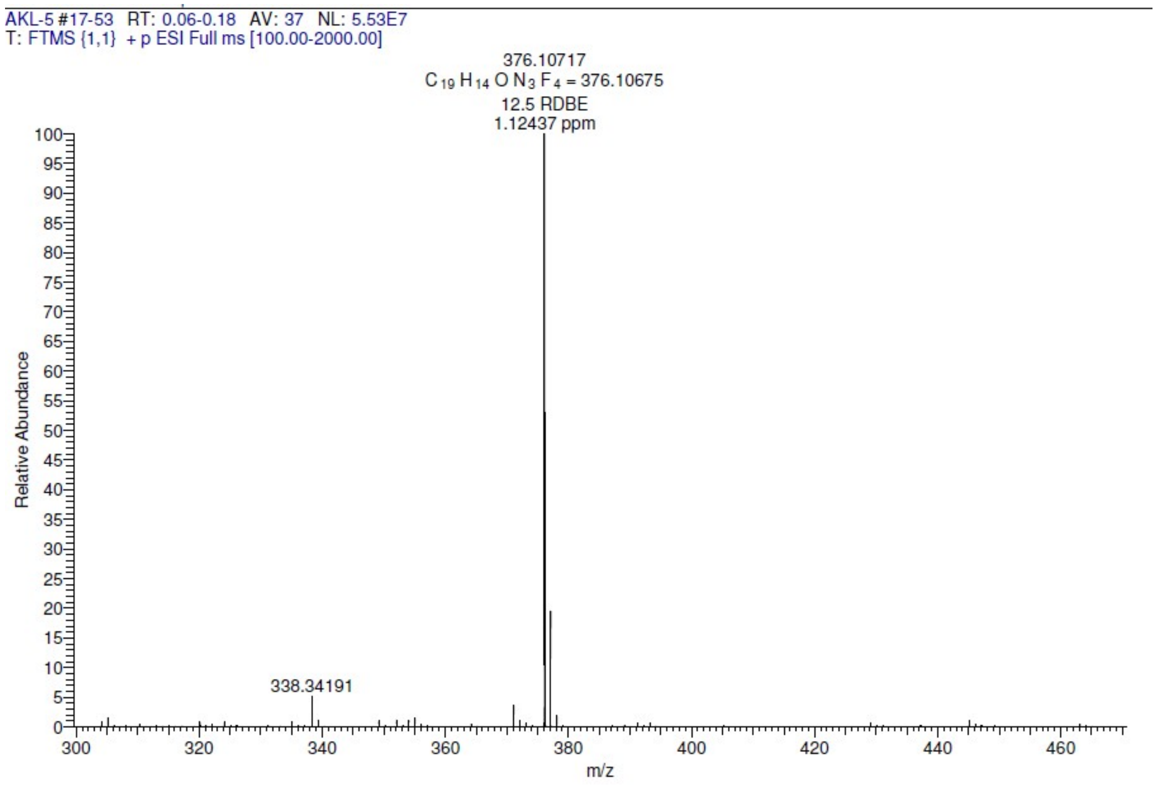

Compound **6w**

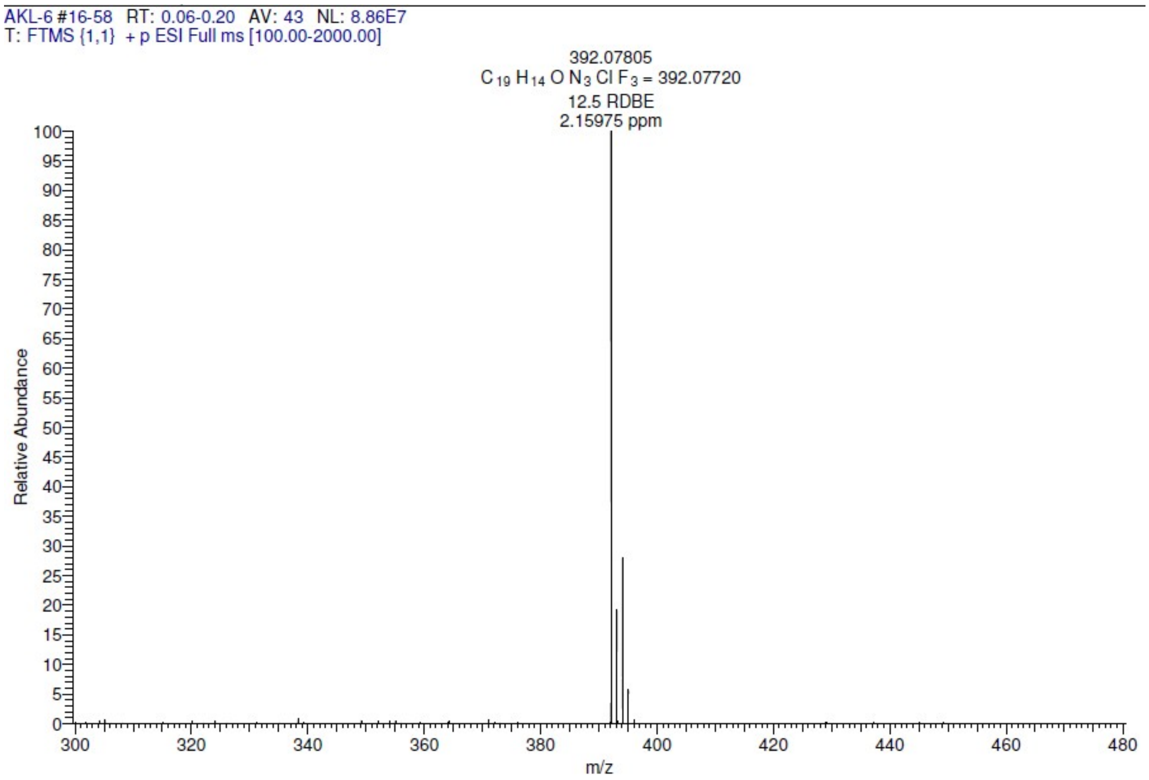

Compound **6x**

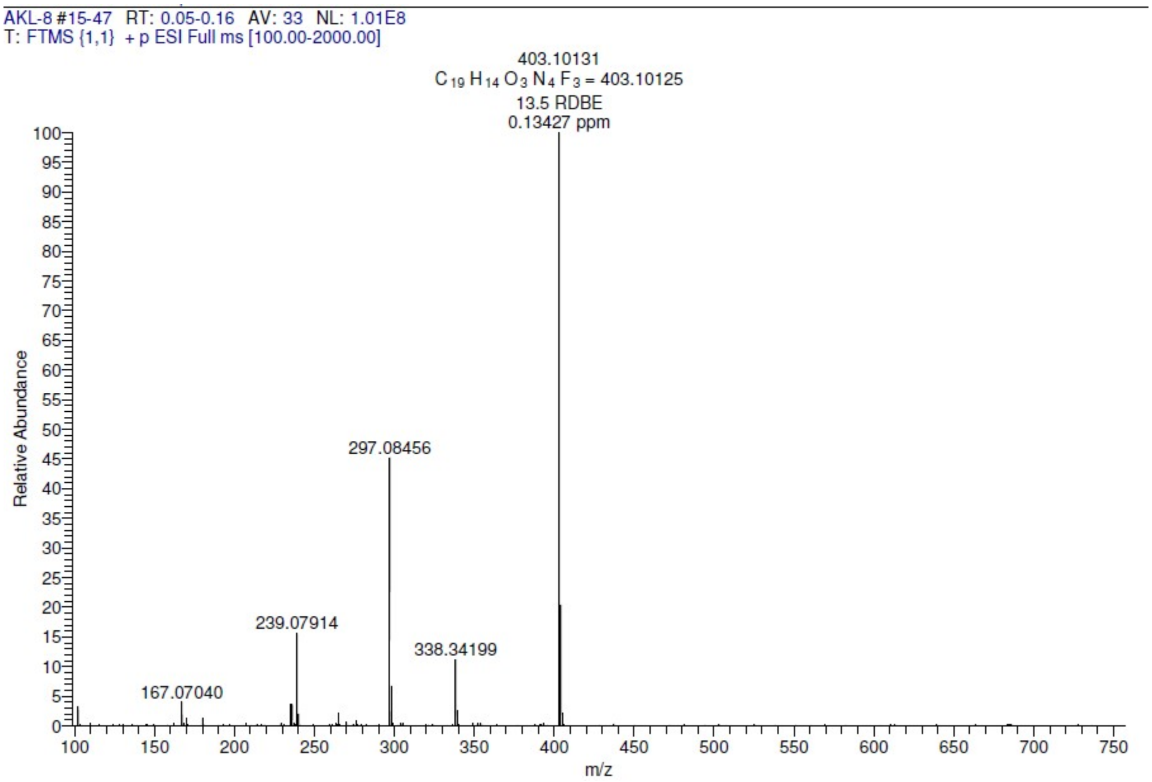

Supplement: RA-016-D5RA09372D-s001 [file RA-016-D5RA09372D-s001.pdf]
